# *Parvimonas micra* can translocate from the subgingival sulcus of the human oral cavity to colorectal adenocarcinoma

**DOI:** 10.1002/1878-0261.13506

**Published:** 2023-09-13

**Authors:** Kelly Conde‐Pérez, Elena Buetas, Pablo Aja‐Macaya, Elsa Martin‐De Arribas, Iago Iglesias‐Corrás, Noelia Trigo‐Tasende, Mohammed Nasser‐Ali, Lara S. Estévez, Soraya Rumbo‐Feal, Begoña Otero‐Alén, Jose F. Noguera, Ángel Concha, Simón Pardiñas‐López, Miguel Carda‐Diéguez, Igor Gómez‐Randulfe, Nieves Martínez‐Lago, Susana Ladra, Luis A. Aparicio, Germán Bou, Alex Mira, Juan A. Vallejo, Margarita Poza

**Affiliations:** ^1^ meiGAbiome, Microbiology Research Group, Servicio de Microbiología Center for Advanced Scientific Research (CICA), Institute of Biomedical Research (INIBIC), University Hospital of A Coruña (HUAC), University of A Coruña (UDC), CIBER of Infectious Diseases (CIBERINFEC‐ISCIII), Hospital Universitario Spain; ^2^ Genomic and Health Department FISABIO Foundation, Center for Advanced Research in Public Health Valencia Spain; ^3^ Database Laboratory Research Center for Information and Communication Technologies (CITIC), University of A Coruña (UDC), Campus de Elviña Spain; ^4^ Pathological Anatomy Service and Biobank University Hospital of A Coruña (HUAC), Institute of Biomedical Research (INIBIC), Hospital Universitario Spain; ^5^ General and Digestive Surgery Service University Hospital of A Coruña (HUAC), Hospital Universitario Spain; ^6^ Periodontology and Oral Surgery Pardiñas Medical Dental Clinic, Cell Therapy and Regenerative Medicine Group, Institute of Biomedical Research (INIBIC) A Coruña Spain; ^7^ Medical Oncology Department University Hospital of A Coruña (HUAC), Maternal and Child Hospital Spain; ^8^ Microbiome and Health Group, Faculty of Sciences University of A Coruña (UDC), Campus da Zapateira Spain

**Keywords:** colorectal cancer, metabarcoding, metatranscriptomics, microbiome, *Parvimonas micra*, periodontal disease

## Abstract

Oral and intestinal samples from a cohort of 93 colorectal cancer (CRC) patients and 30 healthy controls (non‐CRC) were collected for microbiome analysis. Saliva (28 non‐CRC and 94 CRC), feces (30 non‐CRC and 97 CRC), subgingival fluid (20 CRC), and tumor tissue samples (20 CRC) were used for 16S metabarcoding and/or RNA sequencing (RNAseq) approaches. A differential analysis of the abundance, performed with the ANCOM‐BC package, adjusting the *P*‐values by the Holm‐Bonferroni method, revealed that *Parvimonas* was significantly over‐represented in feces from CRC patients (*P*‐value < 0.001) compared to healthy controls. A total of 11 *Parvimonas micra* isolates were obtained from the oral cavity and adenocarcinoma of CRC patients. Genome analysis identified a pair of isolates from the same patient that shared 99.2% identity, demonstrating that *P. micra* can translocate from the subgingival cavity to the gut. The data suggest that *P. micra* could migrate in a synergistic consortium with other periodontal bacteria. Metatranscriptomics confirmed that oral bacteria were more active in tumor than in non‐neoplastic tissues. We suggest that *P. micra* could be considered as a CRC biomarker detected in non‐invasive samples such as feces.

## Graphical abstract

*Parvimonas micra* can translocate from the oral cavity to the intestinal lumen, through the circulatory or gastrointestinal system, where the anaerobic bacteria undergoes some genome and transcriptomic changes needed for the adaptation to its new niche. *P. micra* can be considered as an excellent CRC biomarker detected in non‐invasive samples.

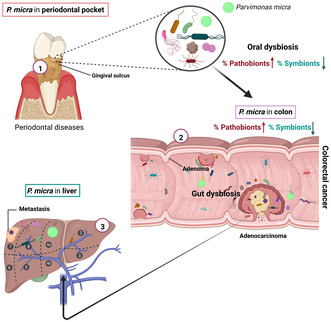

**Abbreviations**
ANI: Average Nucleotide Identity
ASVs: Amplicon Sequence Variants
CARD: Comprehensive Antibiotic Resistance Database
Cas: CRISPR associated proteins
CBCT: Cone Beam Computed Tomography
CHUAC: University Hospital Complex of A Coruña
CMS1: Consensus Molecular Subtype 1
CRC: colorectal cancer
CRISPR: Clustered Regularly Interspaced Short Palindromic Repeats
DAA: differential abundance analysis
EC: enzymatic cocktail
FFPE: formalin‐fixed paraffin‐embedded
gDNA: Genomic DNA
GI: Gastrointestinal
GPD: Gut Phage Database
HUAC: Hospital Universitario de A Coruña
KEGG: Kyoto Encyclopedia of Genes and Genomes
MALDI‐TOF MS: Matrix‐assisted laser desorption/ionization time‐of‐flight mass spectrometry
OrthoANI: Average Nucleotide Identity by Orthology
OVD: Oral Virus Database
Percentage of transcripts: (MTT: metatranscriptome)
RA: relative abundance
RPKM: Number of reads normalized by length of the gene (Kb) and size of the dataset (Mb)
rRNA: Ribosomal ribonucleic acid
UHGG: Unified Human Gastrointestinal Genome

## 1 Introduction

Cancer is a multifactorial disease linked to individual genetic predisposition and environmental factors such as epigenetics and personal lifestyle. In the last 5 years, the role of microbiome in onset, progress and prognosis of cancer disease has been established [1, 2, 3, 4, 5]. Previous studies have reported that gut microbiome dysbiosis could promote inflammation, tissue impairment, and disruption of gastrointestinal (GI) barrier, all of which could lead to carcinogenesis [5, 6]. Similarly, the dysbiosis of oral microbiota has also been proved to be associated not only to oral diseases but also to systemic ones such as colorectal cancer (CRC), the second most deadly type of cancer. In fact, oral microbiota composition differs between CRC patients and healthy individuals and it can be used for CRC prediction [1]. Understanding the correlation between oral microbiome and GI diseases is essential to develop strategies to prevent and treat large intestine cancer [1]. Previous studies identified different periodontal pathogens, such as *Parvimonas micra*, *Porphyromonas gingivalis* or *Fusobacterium nucleatum*, which were over‐represented in CRC tumor tissues, as part of the tumor microbiome (oncobiome) [7]. This consortium of oral microbes, detected in malignant tissues, has been proposed to migrate from the oral cavity to the gut and take part in the adenoma to carcinoma progression promoting polymicrobial and procarcinogenic biofilms, which protects tumor from the host immune system [8].

*Parvimonas micra* is an anaerobe, Gram‐positive commensal cocci normally presented in low‐abundance in the subgingival cavity, the respiratory system, the GI tract and sometimes in urogenital mucosa. Nevertheless, *P. micra* can also act as an opportunistic pathogen in periodontal disease [9, 10, 11], being one of the most predominant species in periodontitis lesions and infected root canals. This microbe has been found in high prevalence in patients suffering from periodontitis, leading to microbial oral dysbiosis and host‐bacteria homeostasis breakdown by disrupting the NOD2 signaling pathway into the host cells [10]. In the subgingival microenvironment, *P. micra* could carry out a “cross‐talk” process with other red and orange complex periodontal pathogens, such as *P. gingivalis*, driving inflammation, gingival bleeding, breakdown of periodontal tissues and, if the disease progresses, tooth loss [11]. In apical chronic periodontitis, *P. micra* and *F. nucleatum* exhibit synergistic biofilm formation [9]. However, virulence mechanisms of *P. micra* and its interactions with other pathogenic bacteria, which may contribute to these diseases, remain unknown.

In previous studies, 16S ribosomal ribonucleic acid (rRNA) gene amplicon sequencing and quantitative PCR showed that *P. micra* was enriched in feces of CRC patients, when compared to feces of healthy individuals, being this bacteria a possible non‐invasive fecal biomarker for CRC early diagnosis [7]. Furthermore, a recent genomic study indicates that *P. micra*, enterotoxigenic *Bacteroides fragilis* and *P. stomatis* could be accurate biomarkers for the presence of laterally spreading tumors [12]. *P. micra* appears in high abundance in tumors specifically characterized by a clear immunological response or Consensus Molecular Subtype 1 (CMS1) [13] and moreover, a recent study demonstrated that high levels of *P. micra* and *F. nucleatum* into CRC tumors were associated with decreased 5‐year cancer survival [14]. Moreover, *P. micra* showed protumorigenic ability in colon cell lines, *Apc* ^*min/+*^ mice and germ‐free mice by an altered Th17 immune response and enhanced inflammatory eukaryotic pathways [15]. In addition, another research indicated that *P. micra* promotes tumor development and cell proliferation by triggering alterations in the immune system, modifications in human DNA methylation status and consequently promoting colon inflammation [16].

Besides all the aforementioned studies, there is still no evidence of how *P. micra* migrates from the oral cavity to the colon. Recent works reported that some *F. nucleatum* isolates from the oral cavity and tumor can be genetically identical, suggesting that this anaerobic microbe could migrate from the oral cavity to the tumor via a transient bacteremia [17]. Mapping the possible routes of these oncobacteria traced from the hypothetical original region to the tumor could be essential to target pathogens before inflammation and damage occurs in colon mucosa.

Most microbiome and GI cancer studies have focused on the microbiome analysis in fecal samples [5], which is a convenient and non‐invasive approximation of the patient intestinal microbial biodiversity, although it is well known that fecal samples do not represent exactly the microbial communities of colon tissues [18]. Last year's works support the correlation between oral and tumor microbiota [1, 17, 19], underlining that further microbiome analyses of oral fluids and colon mucosa tissues in CRC patients are needed in order to study the potential role of anaerobe oral pathobionts in initiation and/or aggravation of CRC. Bacterial RNA sequencing represents a valuable approach, since it allows studying the activity of only the viable microbes presented in different‐nature tissues. Only a few metatranscriptomic analyses of the oncobiome have been developed [20, 21], which calls for an in‐depth characterization of the activity and functions of the tumor microbiome in CRC in order to detect possible protumorigenic bacterial factors.

In this context, the main objective of the present work was to clarify the origin of *P. micra*, as well as quantifying its presence and activity within the colonic tissues. For that, different samples from the oral cavity and colon, including gingival crevicular fluid, saliva, feces, non‐neoplastic colon mucosa, colorectal adenocarcinoma tissues and metastatic tissues, were analyzed using culturomics, genomics and metatranscriptomics as well as 16S rRNA metabarcoding approaches.

## 2 Materials and methods

### 2.1 Sample collection

A total of 93 CRC patients diagnosed after positive CRC colonoscopy from the University Hospital of A Coruña (CHUAC) were enrolled in this study (median age: 68; 61.29% males; 38.71% females) from October 2019 to May 2022. For selection of the correct CRC diagnosed patients, the following exclusion criteria were established: (1) no antibiotics intake in less than 1 month and/or no infectious disease, (2) no chemotherapy and/or radiotherapy treatments prior to colon laparoscopy resection, (3) no genomic predisposition to develop CRC (family history of CRC, Lynch syndrome among others) and/or other malignant lesions, (4) no presence of other gut disorders (such as inflammatory bowel disease), (5) no immunological diseases and (6) no transplants and/or any inmunosupresor treatment. As non‐CRC controls, a cohort of 30 patients (median age: 63; 23.33% males; 76.67% females), mostly cohabitants of the CRC patients and of similar age, who did not present CRC or any other relevant disease, were chosen for the study. An informed consent was signed by all patients. A previous personal interview to all patients and healthy controls involved in the study was conducted for controlling factors such as diet, life habits, other diseases or antibiotics consumption. Besides, medical personnel involved in this study checked the clinical data available in the repository of the Health Service (SERGAS).

Both ~ 20 mL of stool (*n* = 97) and ~ 5 mL of unstimulated saliva (*n* = 94) samples were collected at home by the CRC patients before any treatment or diet and by control participants (28 saliva and 30 fecal samples). Feces were kept in presence of 10 mL of RNA*later* reagent (Thermo Fisher Scientific, Waltham, MA, USA). Both samples were stored in the laboratory at −80 °C until further analysis. Gingival crevicular fluid samples were collected from 20 CRC patients that attended a dental check‐up using sterile paper points ISO 30 (Henry Schein, Melville, NY, USA) that were inserted in the subgingival sulcus of different teeth for 10 s. Eight paper points were placed in an eppendorf tube containing 1 mL of RNAlater (Thermo Fisher Scientific) and frozen at −80 °C until use. Another four sterile endodontic strips were collected and preserved using eSwab™ liquid tubes (Copan Diagnostics, Murrieta, CA, USA) for culturomics procedures. Primary tumor tissue sample (*n* = 20), non‐neoplastic tissue samples from distant areas (colon, *n* = 20 and liver, *n* = 1), colon transition area (interface between non‐neoplastic and adenocarcinoma region, *n* = 1) and metastatic visceral lesions in liver (*n* = 1) were collected from CRC patients through surgical resections at CHUAC. It should be pointed out that at the beginning of both laparoscopic surgeries 2 g of amoxicillin/clavulanic acid were administered as well as during postoperatory (a total of 3 doses every 8 h). All tissue samples were immediately stored in GutAlive collection devices (MicroViable Therapeutics, Gijón, Spain). A total of 20 mg of each type of tissue sample were stored at −80 °C, in presence of 500 μL of RNA*later* reagent (Thermo Fisher Scientific). The remaining tissues were used for immediate culture under anaerobic conditions, as explained below. Also, formalin‐fixed paraffin‐embedded (*FFPE*) tissue samples (non‐neoplastic colon, *n* = 1; adenomas, *n* = 3 and adenocarcinoma tissues, *n* = 2) from the surgical specimens of one patient were selected and studied in the Pathological Anatomy Service of CHUAC and kept at 4 °C until 16S rRNA gene analysis.

### 2.2 Culture media and conditions for *P. micra*

First, a total of 20 tissue samples from different CRC patients were cut into small fragments and immersed in Thioglycollate Fluid Medium (Becton‐Dickinson, Franklin Lakes, NJ, USA) using a sterile scalpel. Afterward, small fragments were homogenized using a glass sterile mortar and pestle until solid tissue fragments were reduced to fine particles. Immediately, the final viscous fluid was vortexed at high speed (2 min). For gingival fluid samples, eSwab™ liquid tubes (Copan Diagnostics) containing four paper points were vortexed directly at maximum speed for 2 min. A few drops of tissues and/or gingival samples were spread on Brucella blood agar plates supplemented with Hemin and Vitamin K_1_ plates (Becton‐Dickinson) and incubated into an anaerobic jar with an atmosphere of 85% N_2_, 5% CO_2_ and 10% H_2_ at 37 °C during at least 2 weeks. Bacteria grown in plates were identified using Matrix‐Assisted Laser Desorption/Ionization Time‐Of‐Flight Mass Spectrometry (MALDI‐TOF MS) at the Microbiology Service (CHUAC). Each colony isolated was spotted into the MALDI plate (Bruker‐Daltonik, Billerica, MA, USA) and treated with formic acid and α‐Cyano‐4‐hydroxycinnamic acid matrix (Bruker‐Daltonik), both prepared following manufacturer's instructions. A protein standard was used (Bruker‐Daltonik) to allow matrix and system calibration. Finally, the plate was introduced in a MALDI Biotyper® Smart instrument (Bruker‐Daltonik). A mean spectrum was constructed and comparison with the spectra contained in the Bruker Biotyper® database (2021) was generated for microbial identification.

### 2.3 Bacterial nucleic acids extraction

#### 2.3.1 DNA extraction from stool and saliva samples

Defrosted stool and saliva samples at room temperature were well‐vortexed and then centrifuged 2 min at 4500 ***g*** and 4 °C. Afterward, 2 mL of supernatants were centrifuged again 10 min at 21 000 ***g*** and 4 °C. Remaining supernatants were stored at −80 °C until use. Final pellets were resuspended in 100 μL of nuclease‐free water. To ensure cellular wall lysis of all different type of bacteria, samples were incubated at 37 °C and 400 rpm during 1 h in the presence of 5 μL of an enzymatic cocktail (EC), containing 20 mg·mL^−1^ of lysozyme (Sigma‐Aldrich, St. Louis, MO, USA), 1.25 KU·mL^−1^ of lysostaphin (Sigma‐Aldrich) and 0.625 KU·mL^−1^ of mutanolysin (Sigma‐Aldrich). DNA from stool and saliva samples was extracted using the MasterPure™ Complete DNA & RNA Purification Kit (Epicentre, Madison, WI, USA).

#### 2.3.2 DNA and RNA extraction from tissue samples and gingival crevicular fluids

DNA and RNA were extracted in parallel from 20 mg of each tissue sample (primary tumor, non‐neoplastic intestinal and liver tissues, transition zone between tumor and normal tissue and metastatic lesions) using the AllPrep® DNA/RNA Mini kit (Qiagen, Hilden, Germany). Tissue homogenization was performed using Lysing Matrix E tubes (MP Biomedicals, Santa Ana, CA, USA) and a 1600 MiniG system (SPEX SamplePrep, Metuchen, NJ, USA) at 2400 ***g*** during 10 min. A total of 30 μL of EC was added to samples after homogenization.

The same procedure was done for DNA and RNA isolation from the gingival fluid sample but without the homogenization step. In these samples, 2 min‐vortexing at high speed was used to remove bacteria from endodontic absorbent strips. After that, papers were discarded carefully and 500 μL of sterile phosphate‐buffered saline were added to the sample. A long centrifugation (21 000 ***g*** during 30 min at 4 °C) was conducted discarding supernatant. The bacteria pellet was used to follow the AllPrep® DNA/RNA Mini kit manufacturer's instructions with the additional enzymatic lysis step (30 μL of EC).

#### 2.3.3 DNA extraction from FFPE samples

From FFPE samples, a total of five histological cuts of 10 μm *per* type of sample were extracted using GeneRead™ DNA FFPE kit (Qiagen) following manufacturer's instructions. After deparaffinization and tissue lysis, the enzymatic treatment was performed adding 17 μL of EC to each sample.

Negative controls (without any type of sample) were done for all nucleic acid extraction procedures explained above. In the case of DNA extraction from FFPE samples, negative controls were performed using a slice of paraffin selected from the margins of FFPE blocks free of any remaining tissue.

#### 2.3.4 Genomic DNA purification from *P. micra*

The Wizard® Genomic DNA Purification kit (Promega, Madison, WI, USA) was used for genomic DNA (gDNA) extraction from *P. micra*. Isolated colonies were transferred to an eppendorf tube containing 500 μL of nuclease‐free water. After a brief centrifugation (22 000 ***g***, 2 min), the pellet was used to start the genomic purification protocol following manufacturer's instructions for Gram positive bacteria. Vortex was not used after cellular lysis to avoid DNA fragmentation.

In all cases, extracted DNA was eluted in EB buffer (Qiagen). RNA from freeze tissues was eluted in RNase‐free water (Thermo Fisher) and stored at −20 °C and RNA at −80 °C until analysis.

### 2.4 16S rRNA sequencing

#### 2.4.1 16S rRNA sequencing for saliva, stool, tissues, and gingival crevicular fluid samples

Two hypervariable regions of the 16S rRNA gene (V3‐V4) were PCR amplified using 5′TCGTCGGCAGCGTCAGATGTGTATAAGAGACAGCC TACGGGNGGCWGCAG as primer forward and 5′GTCTCGTGGGCTCGGAGATGT GTATAAGAGACAGGACTACHVGGGTATCTAATCC as primer reverse using 5 ng·μL^−1^ od DNA template extracted from saliva, stool, tissues, and gingival crevicular fluid samples. Libraries were prepared following the Illumina 16S Metagenomic Sequencing Library Preparation protocol (Illumina, San Diego, CA, USA). Pooled final libraries were diluted to a final concentration of 10 pm and, after, 20% of 10 pm PhiX (Illumina) was added for sequencing using an Illumina MiSeq v3 reagent kit 2 × 300 paired end (Illumina). Quantification of DNA was performed using the Qubit dsDNA HS Assay Kit (Invitrogen, Waltham, MA, USA) and library size was checked using a 2100 Bioanalyzer Instrument (Agilent Technologies, Santa Clara, CA, USA). Negative controls were done in all cases to discard possible contaminations.

#### 2.4.2 16S rRNA sequencing for FFPE samples

For FFPE samples, microbiome analysis was performed by amplifying five hypervariable regions (V2, V3, V5, V6, and V8) following the method previously described by Nejman et al. [3] using 100 ng of DNA as template. Pooled final libraries were diluted to a final concentration of 10 pm and, after, 15% of 10 pm PhiX (Illumina) was added for sequencing using a MiSeq v2 reagent Illumina kit, 2 × 150 paired end (Illumina). In all cases, clean‐up steps were done using AMPure XP beads (Beckman Coulter, Pasadena, CA, USA). Quantification of DNA and library size analyses was checked likewise in the case of the 16S rRNA V3‐V4 gene sequencing. Negative PCR controls were done in all cases to discard possible contamination.

### 2.5 Whole genome sequencing of *P. micra* isolates

Complete genome sequencing of the different isolates of *P. micra*, obtained from tumor tissue and gingival crevicular fluid samples and identified by MALDI–TOF MS, was conducted using two different sequencing platforms; MiSeq (Illumina) and MinION (Oxford Nanopore, Oxford, UK).

An input of 100 ng of gDNA was used to prepare libraries using the Illumina DNA Prep kit (Illumina) and the Nextera® XT Library Preparation kit (Illumina), following in all cases the manufacturer's instructions. Final tagged libraries (12 pm) supplemented with 10% of 12 pm PhiX (Illumina) were 2 × 150 base paired end‐sequenced.

MinION library preparation was performed using the Rapid Barcoding Sequencing kit (Oxford Nanopore) and the Flow Cell Priming kit (Oxford Nanopore) using 60 ng·μL^−1^ of gDNA.

In both cases, DNA quantification and quality determination were conducted as described above for 16S rRNA gene sequencing.

### 2.6 Bacterial transcriptomic analysis in colon tissues

RNA isolated from tissue samples (30 μL) was treated twice with Invitrogen™ Kit TURBO DNA‐free™ DNAse (Thermo Fisher Scientific) for 30 min at 37 °C to eliminate the possible remaining DNA. RNA concentration was measured by the Qubit RNA HS Assay Kit (Thermo Fisher Scientific). Samples were treated with Illumina Ribo‐Zero Plus rRNA Depletion Kit (Illumina) and libraries were obtained and sequenced using NextSeq Illumina Technology (Illumina) (single ends, mid‐output 1 × 150 bp) at the FISABIO sequencing platform (Valencia, Spain).

### 2.7 Bioinformatic analysis

#### 2.7.1 Bioinformatics for microbiome analysis

The quality of all FASTQ files generated from 16S rRNA gene sequencing was checked using fastqc [22]. High‐quality sequences were analyzed using qiime2 (version 2021.11) [23]. First paired‐end reads were trimmed, removing the primers and, in the case of FFPE samples, reads were also demultiplexed into regions by cutadapt tool [24]. In order to correct the Illumina reads errors, remove chimeras and output the Amplicon Sequence Variants (ASVs), DADA2 was used [25]. Taxonomy was generated using SILVA 138 99% reference database [26]. In the case of sequences obtained from FFPE samples, reconstruction of the five small fragments resulting from the sequencing method described by Nejman et al. [3] was conducted using Short MUltiple Regions Framework implementation [27] in qiime2 (version 2021.11) [23] called Sidle [28]. The Sidle pipeline was used, adapting the steps to the characteristics of the dataset obtained in this study by trimming the sequences to a 100 nt length.

In both cases of 16S rRNA sequencing data (sequences obtained from fresh tissues or FFPE samples), each of the ASVs hits of negative controls were subtracted to each corresponding sample. Only bacteria kingdom ASVs were included. After that, typical genus, involved in reagent contamination, were removed from analysis according to the literature [115] as well as two extra contaminant elements that were also detected and excluded (*Dermacoccaceae* and *Acidocella*). In addition, for FFPE samples bioanalysis, a list of common environmental and paraffin contaminants were ousted [3]. Furthermore, ASVs were filtered by an abundance of < 0.01% in 16S V3‐V4 rRNA gene sequencing samples or by a relative abundance (RA) of < 0.0001% in 16S V2, V3, V5, V6 and V8 rRNA sequencing samples. Then ASVs were filtered by intra‐group prevalence (species or genus at least in 50% of samples in V3‐V4 in contrast with a filter of 20% in FFPE samples). Elements not classified at the selected taxon are named with the last known taxon for that ASV and annotated with an extra ‘NA’. The resultant ASVs were grouped and RAs for each sample type was calculated. Barplots were constructed with packages phyloseq (version 1.36.0) [29] and ggplot2 [30] in r (version 4.1) [31]. Moreover, the Venn diagram was constructed using the venndiagram package [32] in r (version 4.1) [31]. Differential abundance analysis (DAA) was performed using r package ANCOM‐BC (version 2.0.1) [33] at genus level with a prevalence cut of 0.1 and adjusting the *P*‐values by the Holm‐Bonferroni method [34].

#### 2.7.2 Bioinformatics for whole *P. micra* genome analysis

A pipeline was created to perform read cleaning and hybrid assembly. Illumina reads were first processed using bbduk (version 38.94) [35] to remove PhiX contamination, compressed losslessly with clumpify (version 38.94) [35] to minify space on disk and trimmed with trimmomatic (version 0.39) [36] for adapter removal and quality control. Read quality was assessed before and after this cleaning process with fastqc (version 0.11.9) [22].

Oxford Nanopore reads were basecalled and demultiplexed using guppy (version 6.0.1) with the high accuracy model, barcoding kit SQK‐RBK004, configuration file dna_r9.4.1_450bps_hac.cfg, doing read splitting and trimming adapters and barcodes. The resulting reads were further cleaned with porechop (version 0.2.4) [37], processed by filtlong (v. 0.2.1) [38] and quality assessed with nanoplot (version 1.38.1) [39] before and after.

The two sets of reads (Illumina and Oxford Nanopore) were then assembled using unicycler (version 0.4.9) [40]. Those that were closed had their start fixed with circlator (version 1.5.5) [41] to facilitate posterior comparisons. They were annotated with PGAP (version 2022‐02‐10.build5872) [42]. Searches against various databases were also performed: Virulence Factor Database (version 2021‐10‐04, considering significant hits those with a nucleotide identity > 40%) [43], Oral Virus Database (OVD, version 2022‐06‐08) [44], Gut Phage Database (GPD, version 2020‐10‐29) [45] and the NCBI database using DIAMOND [46] and BLAST [47]. The different Clustered Regularly Interspaced Short Palindromic Repeats (CRISPR) systems types and its components were identified using CRISPRMiner [48].

#### 2.7.3 Bioinformatics for comparative genomics

Several analyses were performed in order to find the phylogenomic relationship among strains. Twelve non‐metagenomic sequences belonging to *P. micra* and one to *Parvimonas parva* deposited in NCBI database were also included in these series of tests, having their start fixed with Circlator and annotated with PGAP.

Average Nucleotide Identity by Orthology (OrthoANI) was calculated using orthoaniu (version 1.2) [49], whereas the number of variations between the samples and the reference genome was calculated using snippy (version 4.6.0) [50]. Pangenome gene clustering was performed with PIRATE (version 1.0.4) [51]. The produced whole genome alignments were converted to PHYLIP format and then used by raxml (version 8.2.12) [52] to create a phylogenetic tree. Finally, pairwise synteny analyses of closed genomes were performed using synima [53] and sibelia (version 3.0.7) [54] in order to locate large‐scale genome movements and loss or gain of genes.

#### 2.7.4 Bioinformatics for bacterial metatranscriptome analysis of tissue samples

Raw reads were trimmed to remove adapters with Cutadapt 1.18 [24] and then filtered by quality (minimum quality mean 25) and length (minimum length 50) using PRINSEQ [55]. After that, sequences which aligned to the reference human genome (GRCh38.p9) or to the ribosomal database SILVA 132 [26] were detected with bowtie2 (version 2.4.2) [56] (with parameter: –very‐sensitive) and discarded. Remaining reads were mapped to the Unified Human Gastrointestinal Genome (UHGG) database [57], using bowtie2 (version 2.4.2) [56] (with parameter: –very‐sensitive) and samtools (version 1.12) [58] to convert from SAM to sorted BAM. According to the alignments and genes coordinates, we counted (r package GenomicAlignments 1.16.0) [59] the number of hits of each gene in order to build an abundances matrix of genes. An abundances matrix of genomes was also built counting the number of hits in all the contigs of the same genome. A Kyoto Encyclopedia of Genes and Genomes (KEGG, version 2016) [60] annotation was added for each gene. First, the genes were translated into amino acids by means of EMBOSS transeq (version 6.6.0) [61], with translation table 11 (bacterial) and frame 1. Next, each peptide was mapped against the KEGG database with HMMERsearch (version 3.3.2) [62], using a maximum e‐value of 1‐e06. Finally, for each gene, the KEGG annotation corresponding to the best (highest domain score) alignment was selected, with the aid of a custom r script (version 3.6.0) [31].

After the quality filter and the removal of host and ribosomal sequences, a mean of 5.4 × 10^5^ (SD 1 × 10^5^) was obtained for tumor and non‐neoplastic tissue. Among them, 83.25% were annotated to the UHGG database. The minimum number of annotated reads in a sample, 3.95 × 10^5^, was used to calculate rarefaction curves and diversity indexes the Vegan library of r [63]. In order to compare gene expression between samples, the number of hits to a gene was normalized by its length (Kbp) and the size (in Megabp) of the annotated dataset (Reads *per* Kilobase *per* Megabasepair, or RPKM) as previously reported [64]. To identify potentially relevant changes in the level of gene expression between the non‐neoplastic colon and the adenocarcinoma tissue, non‐neoplastic colon tissue from the same patient was used as reference. Fold‐change of gene expression was calculated on a logarithmic scale base 2 (log2FC) [65]. No statistical tests were performed since only one sample per condition was compared. Genes of interest were reannotated with Phyre2 using normal mode on 25 of August 2022 [66].

### 2.8 Ethics statement

To satisfy any ethical or legal consideration, the study was carried out adhering to the standards of good clinical practice and current research regulations included in Law of Biomedical Research 14/2007, in accordance with the principles derived from the latest version of the Declaration of Helsinki and of the Convention on Human Rights and Biomedicine (the Oviedo Convention). Compliance with the protection of personal data of all those involved in the RGPD – UE 2016/679, LOPDGDD 3/2018 Ley 41/2002 and its implementing regulations, Royal Decree 1720/2007, were enforced. This study, that belongs to the project PI20/00413 (ISCIII, Spain), has been approved by the autonomic Research Ethical Committee of Galicia (CEIm‐G 2018/609) and the Spanish Agency for Medicines and Healthcare Products (AEMPS) for the use of human samples from CRC patients of the University Hospital Complex of A Coruña (CHUAC, A Coruña, Galicia, Spain). Signed informed Biobank consents and sample storage were managed by the Biobank of the University Hospital of A Coruña, UNE‐EN ISO 9001‐2015 certified, which ensured the traceability and quality of samples for research use. Clinical data presented in this work were obtained from the repository of Servizo Galego de Saúde (SERGAS) by medical personnel of HUAC. We have the consent of all the CRC patients included in this project for the publication of the obtained results in scientific articles.

## 3 Results

### 3.1 Over‐abundance of *P. micra* in the gut of CRC patients

**Fig. 1:**
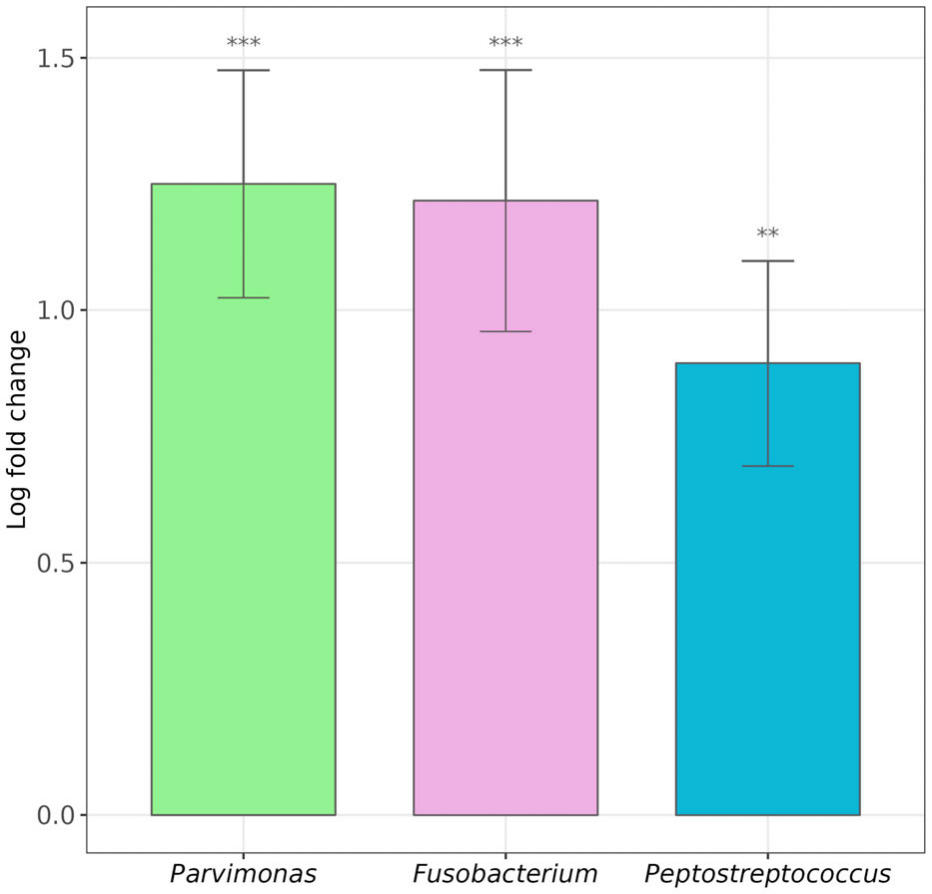
DAA in stool samples between CRC and healthy subjects (CRC group: *n* = 98; Healthy control group: *n* = 30) using ANCOM‐BC at genus level, with a prevalence cut of 0.1 and adjusting the *P*‐values by the Holm‐Bonferroni method. The baseline reference is the healthy control group, meaning a positive Log Fold Change (LFC) indicates a higher abundance of the organism in the non‐reference group, the CRC patients. LFC and its standard error is shown for those taxa with an adjusted *P*‐value under 0.05 (***: <0.001; **: <0.01).

The microbiome analysis made using 16S rRNA metabarcoding procedures in samples obtained from a cohort of 93 CRC patients and 30 healthy controls (Raw Data can be seen at the NCBI SRA database under the accession codes PRJNA911189 and PRJNA893853) revealed that *Parvimonas*, *Fusobacterium*, and *Peptostreptococcus* were significantly more abundant in fecal samples of CRC patients than in feces of non‐CRC individuals (Fig. 1 and Table S1 in the supporting information section). The distribution of the abundance of *P. micra* in CRC patients depending on age and sex has been analyzed (Fig. S1). No significant correlation between the abundance of *P. micra* and sex or age was found. Given the few studies conducted in *Parvimonas* and CRC, we decided to focus on the origin, presence, and activity of this bacterium within CRC patients.

### 3.2 Isolation of *P. micra* strains and genomic comparison

Pairs of gingival crevicular fluid and adenocarcinoma tissues from 20 randomized patients from the 93 patients cohort were used for culture procedures. A total of 11 *P. micra* isolates were obtained from five patients: named 79, 89, 94, 102, and 114 (Table 1), all of them diagnosed with colon adenocarcinoma stage IIA (T3N0). *P. micra* strains isolated from gingival fluid or adenocarcinoma were named using the suffix “‐G” or “‐AC”, respectively, as listed in Table 1. An ending number was added to code the different isolates. For example, PM79KC‐G‐1 corresponded to isolate number 1 from gingival fluid. Patients were named as P79, P89, P84, P102 and P114.

**Table 1:** *Parvimonas* genomes used in this work. Asterisk (*) indicates that S3374 is a *P. parva* strain, used to root phylogenetic trees performed in this work.

Strain	NCBI accession no.	References	Source
PM79KC‐G‐1	CP101407	This study	Gingival crevicular fluid (Patient 79^a^)
PM79KC‐AC‐1	JANDZT000000000	This study	Adenocarcinoma (Patient 79^a^)
PM79KC‐AC‐2	CP101408	This study	Adenocarcinoma (Patient 79^a^)
PM79KC‐AC‐3	JANDZU000000000	This study	Adenocarcinoma (Patient 79^a^)
PM79KC‐AC‐4	JANDZV000000000	This study	Adenocarcinoma (Patient 79^a^)
PM89KC‐G‐1	CP101409	This study	Gingival crevicular fluid (Patient 89^b^)
PM89KC‐G‐2	CP101410	This study	Gingival crevicular fluid (Patient 89^b^)
PM89KC‐AC‐1	CP101411	This study	Adenocarcinoma (Patient 89^b^)
PM94KC‐G‐1	JANDZW000000000	This study	Gingival crevicular fluid (Patient 94^c^)
PM102KC‐G‐1	CP101412	This study	Gingival crevicular fluid (Patient 102^d^)
PM114KC‐AC‐1	CP101413	This study	Adenocarcinoma (Patient 114^e^)
KCOM 1037	CP031971.1	Korean Collection for Oral Microbiology	Postoperative maxillary cyst
NCTC11808	LR134472.1	Wellcome Sanger Institute, UK	Unknown
KCOM 1535	CP009761.1	Korean Collection for Oral Microbiology	Periapical abscess
ATCC 33270	ABEE02	Genome Sequencing Center, USA	GI tract
FDAARGOS 569	RKIS01	USA Food and Drug Administration	Purulent pleurisy
MGYG‐HGUT‐01301	CABKNC01	The European Bioinformatics Institute, UK	Human gut
13–07‐26	BHYQ01	Sunstar Inc., US	Lung abscess
A293	AXUQ01	University of Malaya	Abdominal abscess
EYE 66	JAHXOL01	C. for Disease Control and Prevention, China	Ocular surface
EYE 30	JAHXOH01	C. for Disease Control and Prevention, China	Ocular surface
EYE 29	JAHXOG01	C. for Disease Control and Prevention, China	Ocular surface
EYE 25	JAHXOC01	C. for Disease Control and Prevention, China	Ocular surface
S3374*	JACVDA01	University Medicine Rostock, Germany	Type strain of *P. parva*

^a^ Patient 79 (P79): 63 years old female.

^b^ Patient 89 (P89): 58 years old female.

^c^ Patient 94 (P94): 66 years old male.

^d^ Patient 102 (P102): 84 years old male.

^e^ Patient 114 (P114): 67 years old male.

Complete genomes from all those isolates were compared using other nine *Parvimonas* genomes from the NCBI genome database (Table 1). Average Nucleotide Identity (ANI) comparisons revealed the highest homology between genomes obtained from three strains isolated from patient 89 (P89): PM89KC‐G‐1 and ‐2 and PM89KC‐AC‐1 (Fig. S2 at the supporting information section), showing an identity of 99.23% between PM89KC‐G‐1 and PM89KC‐AC‐1 and 99.25% between PM89KC‐G‐2 and PM89KC‐AC‐1. In patient 79 (P79), identities between PM79KC‐G isolate 1 and PM79‐AC isolates 1–4 were 96.84%, 96.89%, 96.99%, and 97.02%, respectively. In both cases, the genomes of the isolates of each patient (P89 and P79) showed a greater degree of identity between the gingival and the tumor pairs than when compared with the reference genomes at the NCBI database (Fig. S2 in the supporting information section). Genomic guanine and cytosine (G + C) content percentage obtained for genomes of *P. micra* isolates was ~ 29%. Since the isolates from P89 samples showed the highest genomic identity, the corresponding genomes were deeply analyzed in order to support the hypothesis of a common origin of the *P. micra* isolates found in the gingival and in the tumor environments of P89.

### 3.3 Oral origin of *P. micra* isolates found in tumor samples of patient 89

**Fig. 2:**
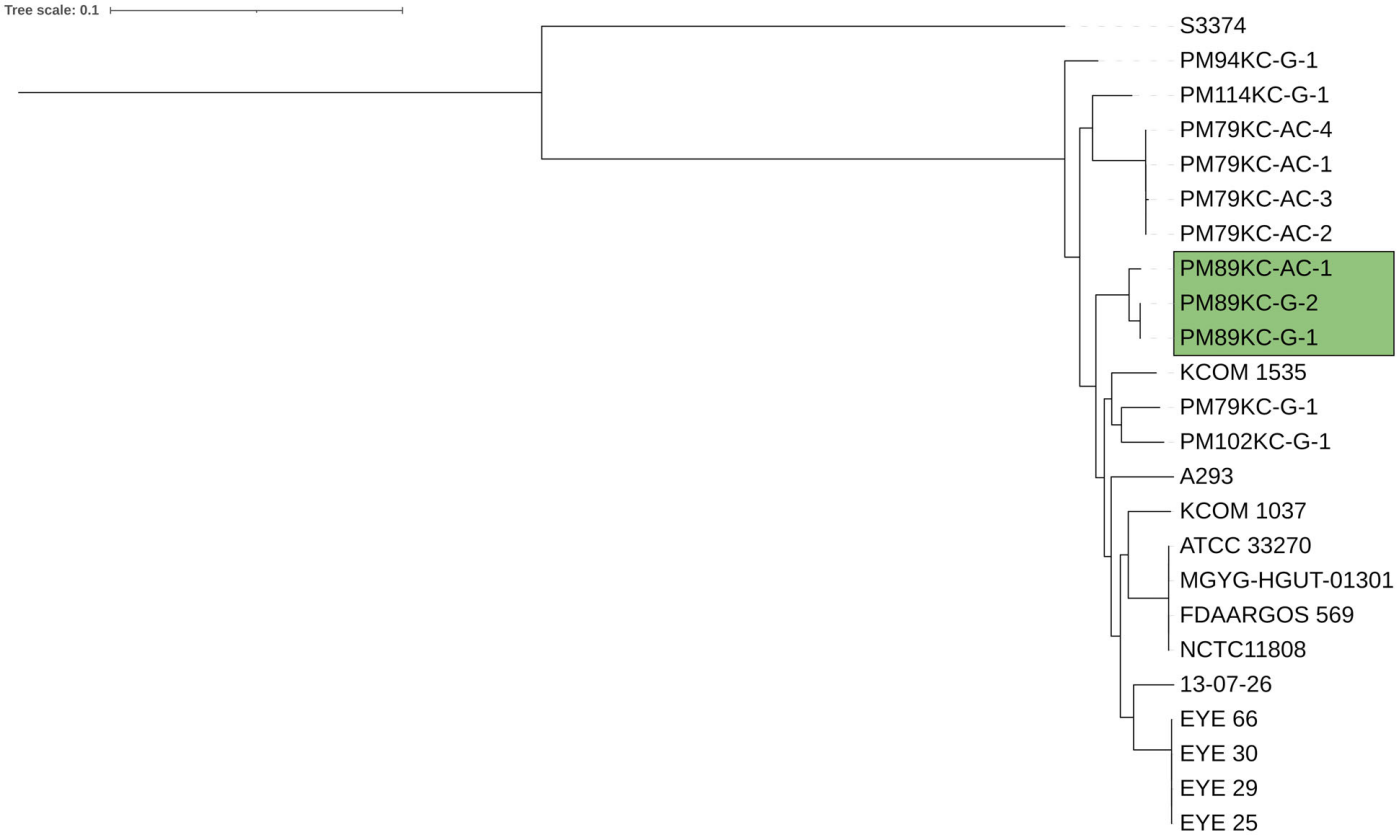
Phylogenetic tree of *Parvimonas* strains. Eleven *P. micra* isolates obtained in this study (Table 1) and twelve *P. micra* genomes obtained from the NCBI database were used. *P. parva* reference genome obtained from the NCBI database was used to root the tree. Isolates from P89 are highlighted in a box.

**Fig. 3:**
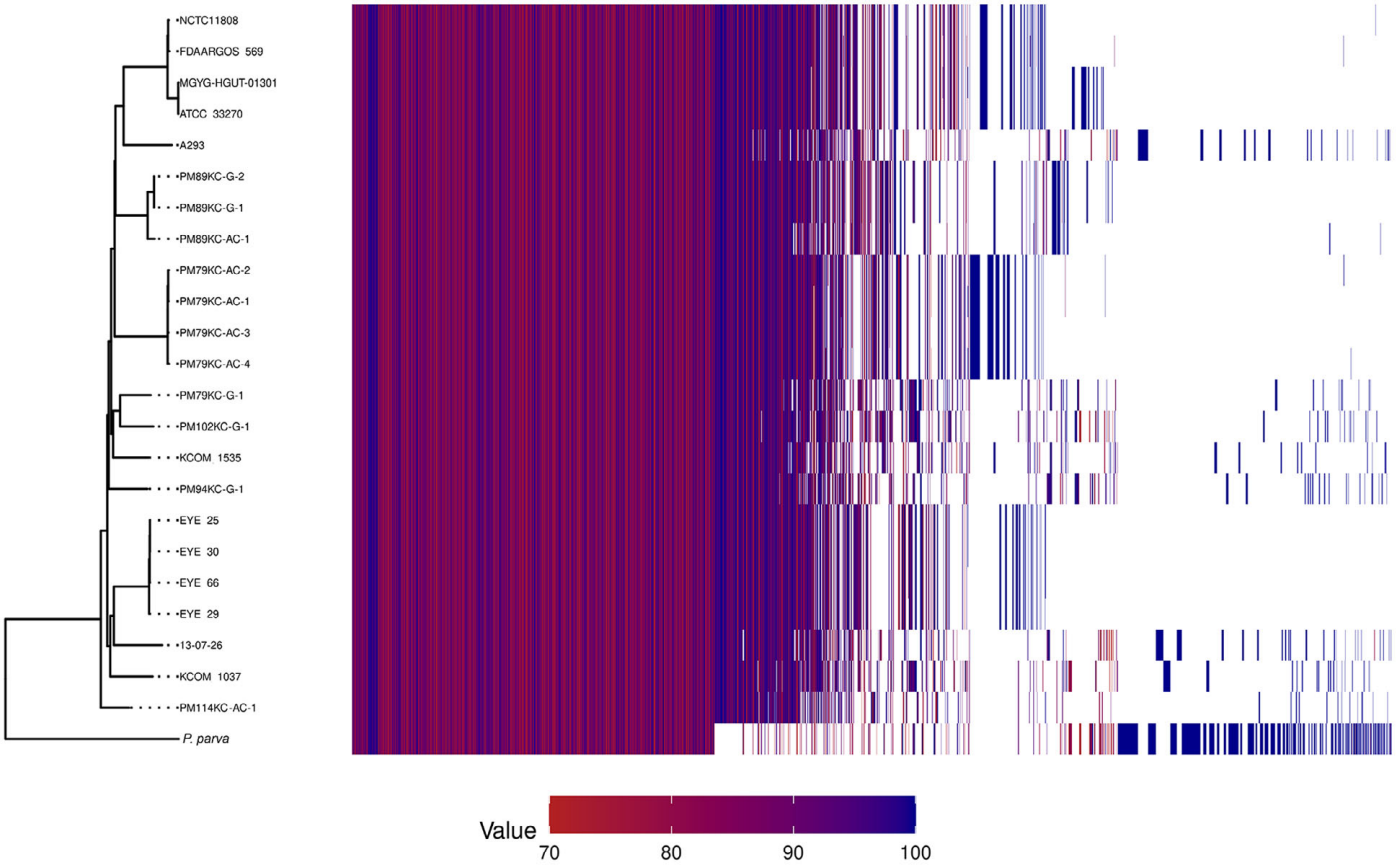
Pangenome clustering of *P. micra* isolates obtained in the present study. Gene clusters are shown as vertical lines, which can be shared among isolates. The tree on the left side is created based on the presence/absence of these clusters. Different colors represent the percentage identity threshold at which the sequences cluster in each gene family (red color represents low identity, blue color high identity and white color represents absence of the gene cluster). Other *P. micra* genomes from the NCBI database were used for comparison. *P. parva* reference genome was used to root the tree.

Based on a whole genome alignment phylogenetic tree (Fig. 2), it can be concluded that isolates from P89 belong to a very well‐differentiated group from other *P. micra* isolates obtained in this study and from the rest of *P. micra* genomes. Furthermore, pangenome clustering revealed differences in the genes shared between strains, including differences between gingival and adenocarcinoma isolates of P89 (Fig. 3). Due to these findings, an in‐depth genome comparison of *Parvimonas* genomes from P89 was conducted. A total of 2120 non‐synonymous mutations were found (Table S2 in the supporting information section), including 1298 single nucleotide polymorphisms, 745 complex mutations, 40 deletions, and 37 insertions, between oral PM89KC‐G isolates 1 and 2, which were virtually identical, and the tumor PM89KC‐AC‐1 isolate. From all these mutations, a total of 1603 genes were affected. When studying presence and absence of genes between oral and tumor isolates from P89, some differences were detected (Tables 2 and 3). The biggest difference corresponded to a fragment of 25 728 bp containing a group of 23 genes found in both oral isolates and absent in the tumor strain (Table 2). Additionally, an identical transposase element repeated at several different genome locations was present in PM89KC‐AC‐1 and absent in the oral isolates PM89KC‐G 1 and 2 (Table 3).

**Table 2:** Missing genes in *P. micra* PM89KC‐AC‐1 isolate when compared to gingival PM89KC‐G 1/2 isolates. Asterisks (*) indicates a group of 23 genes (25 728 bp) belonging to cluster 24 present in the oral isolates (PM89KC‐G 1 and 2) and absent in the tumor isolate (PM89KC‐AC‐1). All listed genes were 100% identical between the two gingival isolates.

Product	Average length (bp)	Cluster	Locus tag	
			PM89KC_G_1	PM89KC_G_2
TetR/AcrR family transcriptional regulator*	573	24	NM219_06315	NM220_06315
ABC transporter ATP‐binding protein/permease*	1740	24	NM219_06310	NM220_06310
ABC transporter ATP‐binding protein/permease*	1710	24	NM219_06305	NM220_06305
ATP‐binding cassette domain‐containing protein*	1518	24	NM219_06300	NM220_06300
Energy‐coupling factor transporter transmembrane protein EcfT*	705	24	NM219_06295	NM220_06295
MptD family putative ECF transporter S component*	585	24	NM219_06290	NM220_06290
Sigma‐70 family RNA polymerase sigma factor*	411	24	NM219_06285	NM220_06285
Hypothetical protein*	162	24	NM219_06280	NM220_06280
Hypothetical protein*	567	24	NM219_06270	NM220_06270
Exonuclease domain‐containing protein*	1872	24	NM219_06265	NM220_06265
Hypothetical protein*	1935	24	NM219_06260	NM220_06260
Hypothetical protein*	885	24	NM219_06255	NM220_06255
Hypothetical protein*	618	24	NM219_06250	NM220_06250
Hypothetical protein*	1113	24	NM219_06245	NM220_06245
Hypothetical protein*	387	24	NM219_06240	NM220_06240
InlB B‐repeat‐containing protein*	1809	24	NM219_06235	NM220_06235
Putative ABC transporter permease*	717	24	NM219_06230	NM220_06230
Branched‐chain amino acid transport system II carrier protein*	1287	24	NM219_06225	NM220_06225
O‐acetylhomoserine aminocarboxypropyltransferase/cysteine synthase*	1290	24	NM219_06220	NM220_06220
DKNYY domain‐containing protein*	1479	24	NM219_06215	NM220_06215
LPXTG cell wall anchor domain‐containing protein*	2193	24	NM219_06210	NM220_06210
MBL fold metallo‐hydrolase*	810	24	NM219_06205	NM220_06205
Aldehyde dehydrogenase*	1362	24	NM219_06200	NM220_06200
Hypothetical protein	324	25	NM219_00070	NM220_00070
IS200/IS605 family transposase	459	25	NM219_00075	NM220_00075
Hypothetical protein	1104	25	NM219_00080	NM220_00080
DUF1307 domain‐containing protein	486	26	NM219_00915	NM220_00915
DUF1307 domain‐containing protein	477	26	NM219_00920	NM220_00920
DUF2087 domain‐containing protein	270	27	NM219_01675	NM220_01675
GNAT family N‐acetyltransferase	480	27	NM219_01680	NM220_01680
ABC transporter permease	1263	28	NM219_06715	NM220_06715
Hypothetical protein	636	29	NM219_03355	NM220_03355
Hypothetical protein	372	30	NM219_00160	NM220_00160
Hypothetical protein	183	31	NM219_03780	NM220_03780
ABC transporter ATP‐binding protein/permease	1611	32	NM219_01745	NM220_01745
Hypothetical protein	1638	33	NM219_06615	NM220_06615
Hypothetical protein	198	34	NM219_00320	NM220_00320
Hypothetical protein	432	35	NM219_00645	NM220_00645
Hypothetical protein	147	36	NM219_03720	NM220_03720
CPBP family intramembrane metalloprotease	678	37	NM219_06015	NM220_06015
GntR family transcriptional regulator	150	38	NM219_02485	NM220_02485
Hypothetical protein	180	39	NM219_07000	NM220_07000
Hypothetical protein	384	40	NM219_06370	NM220_06370

**Table 3:** Gained genes in the adenocarcinoma PM89KC‐AC‐1 isolate when compared to the PM89KC‐G 1 and 2 gingival isolates. Asterisks (*) tag an identical transposase element repeated at several different genomic locations present in the tumor isolate (PM89KC‐AC‐1) and absent in the gingival isolates (PM89KC‐G 1 and 2.

Consensus product	Average length (bp)	Cluster	Locus tag
Recombinase family protein	621	41	NM221_06205
SpaA isopeptide‐forming pilin‐related protein	2271	41	NM221_06200
Pseudouridine‐5′‐phosphate glycosidase	546	42	NM221_06515
ABC transporter ATP‐binding protein/permease	1560	42	NM221_06510
EXLDI protein	369	43	NM221_01675
Transposon‐encoded TnpW family protein	231	43	NM221_01680
Restriction endonuclease subunit S	1242	44	NM221_00825
Hypothetical protein	435	45	NM221_00630
Rhodanese‐like domain‐containing protein	1083	46	NM221_01690
Hypothetical protein	1017	47	NM221_05350
IS630 family transposase*	1194	48	NM221_00050; NM221_00875; NM221_01125; NM221_01455; NM221_02510; NM221_06115; NM221_07165; NM221_07365
ABC transporter ATP‐binding protein/permease	1617	49	NM221_06020
Hypothetical protein	360	50	NM221_06500
Hypothetical protein	2496	51	NM221_01205
ParB/RepB/Spo0J family partition protein	606	52	NM221_07790
Relaxase/mobilization nuclease domain‐containing protein	1332	53	NM221_06215

**Fig. 4:**
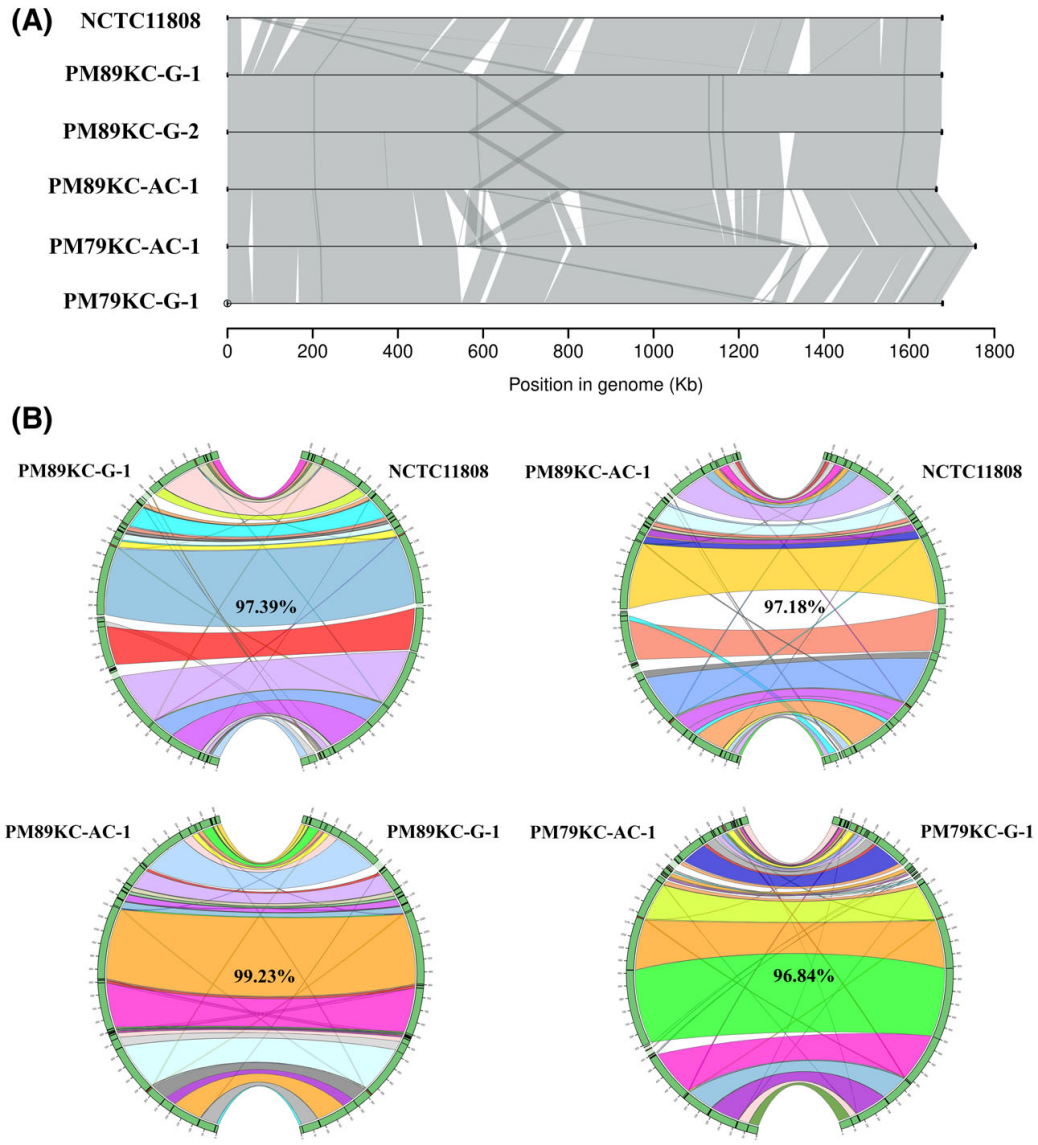
Physical co‐localization of genetic loci for closed genomes through synteny analysis. (A) Detailed pairwise synteny analysis showing genomic rearrangements and loss or gain of genes between isolates. A reference *P. micra* genome (NCTC11808) was used to compare synteny of each paired gingival and adenocarcinoma *P. micra* genomes from CRC patients 79 (P79) and 89 (P89). A white space represents loss or gain of genes, while gray represents synteny and darker gray indicates movement or duplications. A position can link to more than one zone, corresponding to the same position in the other genome and a duplication at another location in the same genome, as can be seen between PM89KC‐G‐2 and PM89KC‐AC‐1 in the cross‐shaped structure flanking positions 600 and 800 Kb in the genome. (B) Large‐scale pairwise synteny analysis showing big differences between genomes in a circos plot. Self‐hits have been removed to facilitate visualization. Some small hits, corresponding to repetitive or very small areas of the genome, have been allowed in order to show other small rearrangements.

Synteny analysis also revealed loss and gain of genes, but more interestingly, a specific cross‐shaped structure in PM89KC isolates (Fig. 4A and Fig. S3 in the supporting information section), composed of a repeat region of 30 genes, corresponded to a shared region in two very similar prophages, where manual inspection revealed multiple genes involved in genomic mobility and recombination but none involving capsid or tail virus formation (Table S3 in the supporting information). Moreover, if KCOM 1037 strain was taken as a reference, the left prophage has been inserted into a CRISPR array of a type III‐B CRISPR‐Cas system in all P89 isolates (Fig. S3 in the supporting information section), separating the Cas proteins (CRISPR associated proteins) from the CRISPR array. Additionally, the tumor isolate has an extra CRISPR array and more spacers when compared to the subgingival isolates (Table S4 in the supporting information).

This “genomic cross” was composed of a repeat region of 30 genes (pairwise identity of ~ 80%) flanking the ~ 600 Kbp and ~ 800 Kbp positions (Table S3), present in all P89 isolates. These repeats corresponded to a shared region in two very similar prophages, where manual inspection revealed multiple genes involved in genomic mobility and recombination (recombinase, replication initiator protein, DNA binding protein, conjugal transfer protein, topoisomerase, helicase, relaxases, relaxosome proteins, helix‐turn‐helix transcriptional regulator and sigma 70 family RNA polymerase sigma factor), but none involving capsid or tail virus formation. These prophages have also been detected in other *P. micra* isolates in different positions, although usually not duplicated. When the whole left prophage of PM89KC‐AC‐1 (~ 42 Kbp, position 564183…607520) was compared against OVD and the GPD (minimum identity ≥ 50%), significant hits (e‐value of 0 and bitscore over 3000) were found with a median identity of 89.226 ± 4.65% in OVD. The top hits belonged to a 42 456 bp unclassified prophage (median identity of 89.77 ± 2.35%). The search did not return hits in the GPD. Additionally, putative virulence factors were detected in the prophages, including an ABC transporter ATP‐binding protein and a Type IV secretion system (including associated proteins PcfB and PrgI). Other relevant proteins found were a NlpC/P60 family protein in the left prophage and a CHAP domain containing protein in the right prophage. These prophages shared a large proportion of genes, but the left one had several extra proteins (Table S3). If KCOM 1037 strain was taken as a reference, the left prophage has been inserted into a CRISPR array of a type III‐B CRISPR‐Cas system in all P89 isolates (Fig. S3), separating the Cas proteins (CRISPR‐associated proteins) from the CRISPR array. However, the PM89KC‐G and PM89KC‐AC‐1 isolates differed in the number and length of CRISPR arrays on both sides of the prophage. Isolate PM89KC‐G‐1 presented two CRISPR arrays after the prophage. The first one (G‐A), of 298 bp, is located at position 598786, and the second one (G‐B), of 423 bp, at position 601696. Isolate PM89KC‐AC‐1 presented these two arrays after the prophage: the first one (AC‐B), of 2213 bp, located at position 607555, and second one (AC‐C), of 423 bp, at position 612379, and an extra array (AC‐A, position 563270, 749 bp) before the prophage (Fig. S3). Arrays G‐A and G‐B from PM89KC‐G‐1 corresponded to arrays AC‐B and AC‐C in PM89KC‐AC‐1. The repeats in all these arrays shared the same core structure (Table S3). All spacers inside array G‐B from PM89KC‐G‐1 were exactly identical to the ones in array AC‐C in PM89KC‐AC‐1 and the last 3 spacers from AC‐B are the ones in G‐A. Furthermore, when searching for these spacers in other *P. micra* isolates, none contained the exact same ones, and were only shared in P89. If the subgingival isolates were taken as reference origin, the adenocarcinoma isolate could have increased the size of array G‐A from 3 spacers to 32 and gained the extra array AC‐A with 11 spacers (total net gain of 40 spacers). The first two spacers of AC‐A on the left of the prophage for PM89KC‐AC‐1 were also detected before the prophage in PM89KC‐G (G‐Res), further supporting the common origin of these isolates. However, when comparing spacers from EYE_30 and PM79KC‐G‐1, many were identical, which indicated that sharing spacers across isolates is not uncommon and cannot be used as definitive proof. When the new spacers of PM89KC‐AC‐1 were searched against OVD and GPD, a couple in array AC‐B matched unclassified phages in genus Coprococcus and Tyzzerella (both from family Lachnospiraceae) in the GPD. The rest matched oral phages in Parvimonas, Streptococcus, and Fusobacterium in OVD or intestinal phages in Parvimonas in GPD. Finally, a similar insertion and duplication of this prophage has been seen in other *P. micra* such as PM102KC‐G‐1, but in a different CRISPR‐Cas system (Type CAS‐II‐A/CAS‐III‐A) with different spacers and repeated sequences (Table S4).

Potential virulence factors were analyzed in gingival and adenocarcinoma isolates of P89 (Tables S5 and S6 in the supporting information section). Most virulence factors were shared between PM89KC strains including multiple iron scavenging and transport proteins, type III, IV and VII secretion systems, colibactin toxins, neutrophil activating proteins, tissue adhesins, peptidases, and biofilm regulators. It is important to note that after the liver metastasis diagnosis for P89, our team collected during the laparoscopy surgery, metastasis and non‐metastasis liver samples in order to culture both specimens and we performed satisfactorily an in‐depth 16S rRNA gene metabarcoding analysis. However, efforts to isolate *P. micra* from liver samples were unsuccessful, probably due to the fastidious nature of this pathobiont or to the antibiotic pre‐surgery treatment.

The above results suggest a common origin of the adenocarcinoma and the gingival *P. micra* isolates of P89.

### 3.4 Clinical background of patient 89

**Fig. 5:**
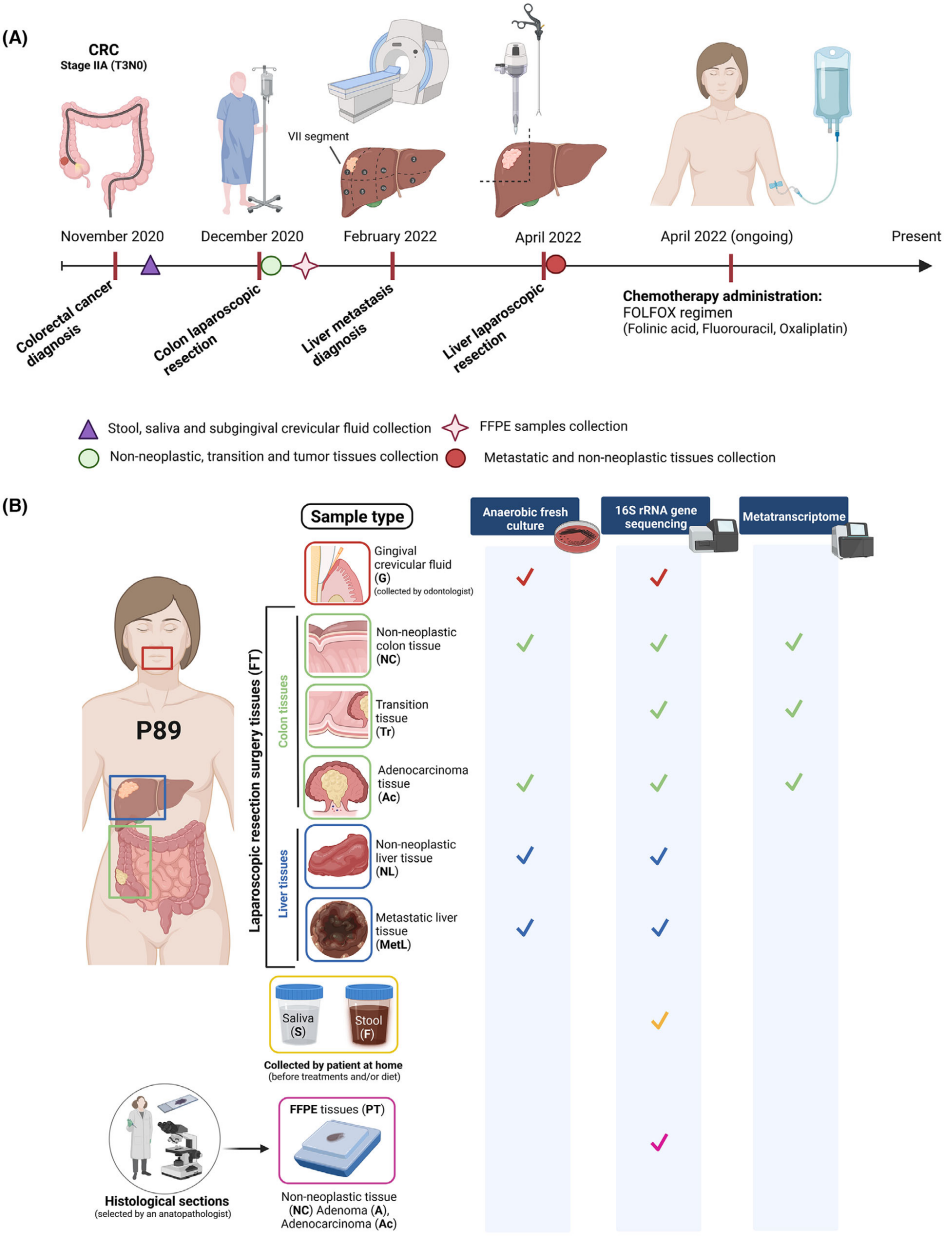
Overview of patient 89 (P89): clinical progression and different approaches performed for the different‐nature samples. (A) Timeline depicting the month and year at which P89 was diagnosed, underwent different surgeries or started treatment. The pickup time of the different‐nature samples analyzed in this work are also shown. (B) Diagram showing the nine different samples that were analyzed to define the microbiome composition of P89 patient and follow *P. micra* trace throughout a CRC patient. Both images (A and B) were created with BioRender (biorender.com).

**Fig. 6:**
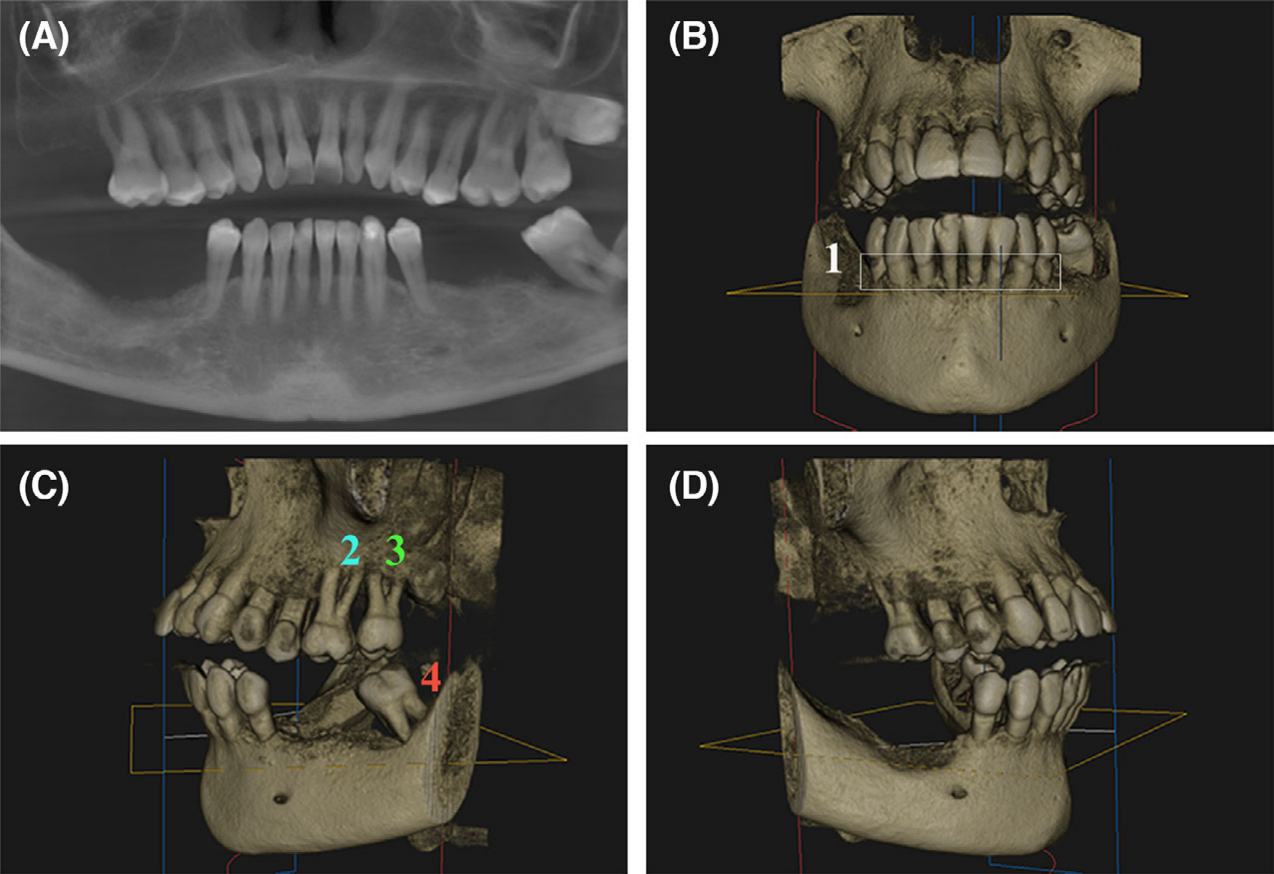
CBCT of CRC patient 89 (P89). (A) Panoramic x‐ray reconstruction obtained from the CBCT. (B) Frontal view 3D image from the CBCT. Number 1 shows a generalized horizontal bone loss affecting the lower anterior teeth with a 50% of bone loss. (C) Left lateral view 3D image from the CBCT image. Numbers 2, 3, 4 indicate areas with high grade furcation involvement. (D) Right lateral view 3D image from the CBCT image.

At this point, we decided to explore the patient 89 in depth. The patient 89 (P89) was a 58‐year‐old woman, diagnosed with CRC in November 2020 in CHUAC (Spain). This woman underwent a colon resection by laparoscopy in December 2020 (Fig. 5A). Primary tumor, an intestinal ulcerated adenocarcinoma, was located on right colon (cecum) and classified as stage IIA (T3N0). For this CRC patient, adjuvant chemotherapy treatment was not administered before resection. No vascular and/or perineural invasion was detected. Furthermore, three tubular low grade dysplasia adenomas were located throughout the right colon section. The histopathological study revealed an intestinal adenocarcinoma with low grade tumor budding. Primary tumor presented a driver mutation in *KRAS* gene (G12X). No alterations in expression of DNA mismatch repair proteins (coded by *MLH1*, *MSH2*, *MSH6* and *PMS2* genes) or mutations in *NRAS*, *BRAF* genes were detected. No microsatellite instability was found. Moreover, in February 2022, this patient was diagnosed with liver metastasis (VII segment) and surgical resection was performed in the same hospital. Molecular analyses in metastasis biopsy did not detect any mutations in *KRAS*, *NRAS*, or *BRAF* genes or microsatellite instability. The expression of mismatch repair proteins was also preserved. No lymph node affectation was detected in this patient even before any neoplastic lesion in the liver was noticed. All these clinical data were obtained from the Pathological Anatomy and General and Digestive Surgery Services of CHUAC (Spain). Figure 5 shows a graphic summary of all different‐nature samples from patient 89 used in this study as well as the different approaches developed for each kind of sample. An intraoral and extraoral examination of the patient was performed. Clinical measurements of probing depth, clinical attachment level, and bleeding on probing were recorded at six points around each tooth. Miller's mobility index was recorded for each tooth. A Silness Loe score of 0.3 for bleeding on probing and a CPO index for dental caries (tooth cavities) of 7 were obtained, indicating that the patient had a high degree of tooth decay and tooth loss. The Cone Beam Computed Tomography (CBCT) from the patient's upper and lower jaw (Fig. 6) revealed an advanced degree of bone loss, the furcation involvement of teeth numbers 16, 26, 27 and 38, and the absence of teeth numbers 18, 17, 28, 35, 36, 37, 46, 47 and 48. Figure 6 shows the generalized horizontal bone loss and the high‐grade furcation involvement detected in the oral cavity of P89 after oral exploration. Based on these data, a diagnosis of periodontitis in stage IVB was made, according to the 2017 World Workshop on the Classification of Periodontal and Peri‐Implant Diseases and Conditions [65].

### 3.5 Microbiome of patient 89

Further, we decided to explore the microbiome of the CRC P89. For this, we performed a deep analysis of the bacterial 16S rDNA in feces, saliva, subgingival fluid and non‐neoplastic, transition and adenocarcinoma tissues as well as in metastatic and non‐neoplastic liver regions (Fig. 5).

**Fig. 7:**
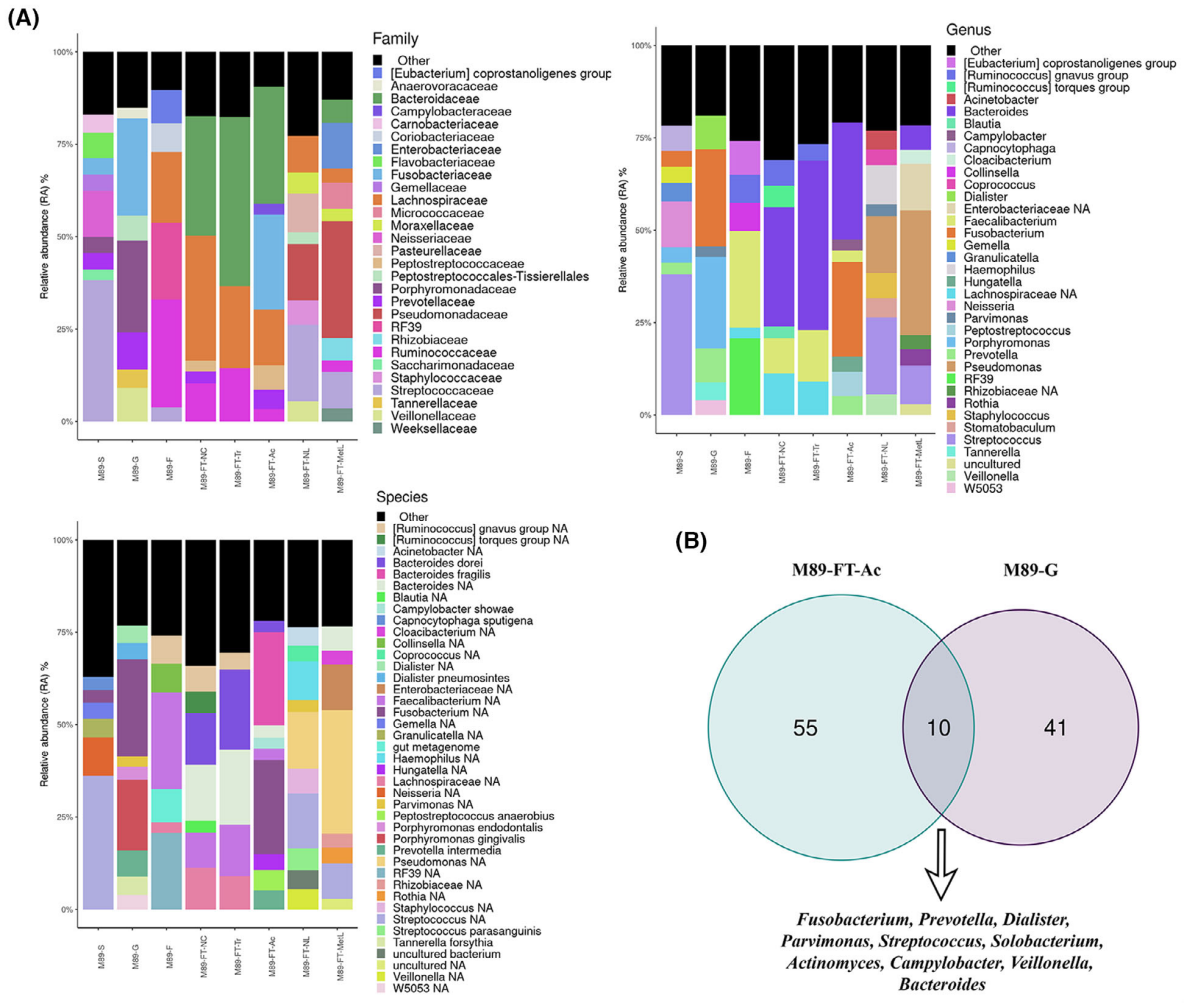
Microbiome composition of CRC patient 89 (P89) obtained by 16S rRNA Illumina sequencing. (A) Barplots at family‐level (upper left), genera‐level (upper right) and species‐level (bottom left) showing the different phylotypes across samples from P89. Only microorganisms with a RA over 3% are listed in all graphics, all bacteria detected with RA lower than 3% are grouped into the category “Others”, colored in black. (B) Venn's diagram indicates the number of different genera found in adenocarcinoma (M89‐FT‐Ac) and subgingival crevicular fluid sample (M89‐G) as well as the number of shared bacteria between both samples (listed below). Samples analyzed were saliva (M89‐S), gingival crevicular fluid (M89‐G), feces (M89‐F), non‐neoplastic colon tissue (M89‐FT‐NC), colon transition tissue (M89‐FT‐Tr), adenocarcinoma (M89‐FT‐Ac), non‐neoplastic liver tissue (M89‐FT‐NL), and metastatic visceral lesion in liver (M89‐FT‐MetL).

The microbiome analysis of feces (M89‐F) revealed that *Faecalibacterium* (26.16%), *Enterococcaceae* bacterium RF39 (20.73%), *Eubacterium coprostanoligenes* group (9.02%), *Collinsella* (7.71%) *Ruminococcus gnavus* group (7.61%), *Lachnospiraceae* (2.86%), and *Bacteroides* (2.47%) were the most abundant bacteria (Fig. 7).

Additionally, the taxonomic assignment of the saliva sample (M89‐S) sequences showed that the most common genera were *Streptococcus* (38.04%), *Neisseria* (12.36%), *Capnocytophaga* (6.83%) and *Granulicatella* (4.97%) followed by *Gemella* (4.40%), *Fusobacterium* (4.33%) and *Porphyromonas* (4.24%). When gingival crevicular fluid sample (M89‐G) was analyzed, the genus *Fusobacterium* (26.30%), *Porphyromonas* (24.73%), *Prevotella* (9.15%), *Dialister* (9.05%), *Tannerella* (4.94%), *Parvimonas* (2.83%) and a member of the *Peptostreptococcaceae family* (*Peptostreptococcaceae* bacterium oral taxon 113 str. W5053 at 3.93%) were found as the most abundant bacteria (Fig. 7). Two bacteria species pertaining to the red complex group, *P. gingivalis* and *Tannerella forsythia*, were detected in both M89‐S and M89‐G samples, as well as other important periodontal pathogen species such as *Porphyromonas endodontalis*, *Prevotella intermedia*, *Dialister pneumosintes*, *Eubacterium nodatum* or *Mogibacterium timidum* (Fig. 7).

Fresh tissue adenocarcinoma sample (M89‐FT‐Ac) collected during the laparoscopy surgery was also analyzed. The tumor microbiome, mostly shaped by anaerobic bacteria, was composed of five major genera: *Bacteroides* (31.69%), *Fusobacterium* (25.62%), *Peptostreptococcus* (6.49%), *Prevotella* (5.17%) and *Hungatella* (4.16%) (Fig. 7). In addition, transition tissue (M89‐FT–Tr) and non‐neoplastic colon mucosa tissue (M89‐FT‐NC) were collected. The analysis of the PM89‐FT–Tr sample revealed that its microbiome was mainly composed by *Bacteroides* (45.80%), *Faecalibacterium* (13.92%), *Lachnospiraceae* (9.03%), *Ruminococcus gnavus* group (4.51%), *Ruminococcus torques* group (2.02%), *Barnesiella* (1.91%), *Faecalitalea* (1.64%), *Peptostreptococcus* (1.49%), and *Prevotella* (1.45%). Besides, the M89‐FT‐NC microbiome was composed by similar bacteria but with a significantly different abundance when compared to transition and adenocarcinoma tissues (Fig. 7). For example, the bacteria *B. fragilis*, over‐represented in Ac sample (25.17%), showed low abundance in normal colon tissue sample (1.66%) and also in M89‐FT‐Tr tissue (1.72%). This tendency was also observed for other microorganisms such as *Peptostreptococcus anaerobious*, which was over‐represented in adenocarcinoma (5.58%) and low abundant in transition (0.70%) and normal (0.82%) colon tissues. *P. intermedia* was the third most abundant species identified in adenocarcinoma (5.17%) but it was less frequent in transition (1.45%) and in normal (1.19%) mucosa tissues. This happened also for *C. showae* and *Streptococcus agalactiae*, which showed higher abundance in M89‐FT‐Ac sample (2.96% and 0.99%, respectively), while their presence in M89‐FT‐Tr tissue (0.08% and 0.11%, respectively) or in M89‐FT‐NC tissue (0.05% and 0.09%, respectively) was very low. In contrast, the microorganism *B. dorei* appeared with higher RA in M89‐FT‐Tr tissue (21.73%) or in M89‐FT‐NC tissue (14.00%) compared to M89‐FT‐Ac (3.06%) (Fig. 7).

The microbiome analysis performed in the liver sample revealed that *Streptococcus* (20.91%), *Pseudomonas* (15.46%), *Haemophilus* (10.61%), *Staphylococcus* (6.75%), and *Veillonella* (5.54%) were the main genera found in the non‐neoplastic adjacent tissue (M89‐FT‐NL) while in the metastatic region (M89‐FT‐MetL), *Pseudomonas* (33.74%), *Streptococcus* (10.51%), *Bacteroides* (6.63%) and *Rothia* (4.34%) were the most represented genera.

It is important to note that periodontal pathogens genera, such as *Parvimonas*, *Prevotella* and *Eubacterium*, were detected, in some cases with low abundance, in liver samples of P89.

*Parvimonas* was found in colon in adenocarcinoma, non‐neoplastic and transition tissues (1.24%, 0.65%, and 0.27%, respectively) being enriched in cancerous tissue. *Parvimonas* was also present in feces (1.00%), saliva (1.34%), and subgingival crevicular sample (2.83%). In the liver sample, *Parvimonas* was detected with a RA of 3.18%. *P. micra* was grown after culturing gingival and colorectal adenocarcinoma but no *P. micra* colonies were obtained from the liver tissue.

As commented before, other typical oral pathogens were detected in all types of samples, being the most abundant *Fusobacterium*, *Prevotella*, *Campylobacter*, and *Dialister*. Figure 7B shows bacteria shared between gingival and adenocarcinoma samples.

**Fig. 8:**
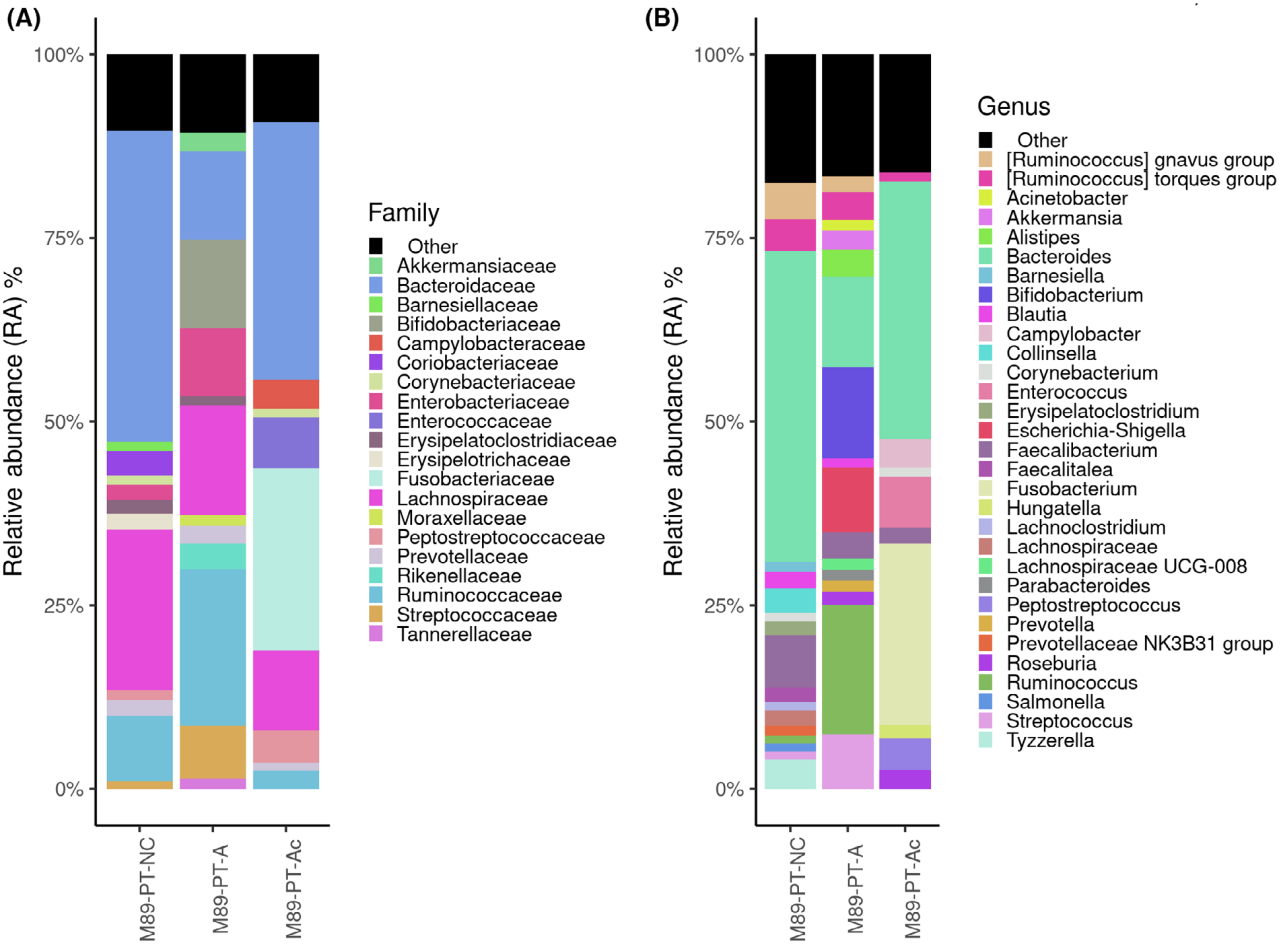
Microbiome composition of FFPE samples of patient 89 (P89) obtained by 16S rRNA Illumina sequencing. (A) Barplots at family‐level and (B) barplots at genera‐level. Only microorganisms with a RA over 3% are listed in both graphics. Bacteria detected with RA lower than 3% are grouped into the category “Others”, colored in black. FFPE samples analyzed were non‐neoplastic colon mucosa (M89‐PT‐NC), adenoma tissue (M89‐PT‐A) and adenocarcinoma tissue (M89‐PT‐Ac).

Moreover, FFPE samples from tissue samples of P89 revealed a similar bacterial composition (Fig. 8). Typical oral microbes were detected in those FFPE samples, as well as in non‐paraffin embedded samples, such as *Prevotella* (PT‐NC: 0.81%; PT‐A: 1.53%; PT‐Ac: 0.99%), *Fusobacterium* (PT‐NC: 0.32%; PT‐A: 0.61%; PT‐Ac: 24.73%), *Dialister* (PT‐NC: 0.05%; PT‐Ac: 0.03%), *Actinomyces* (PT‐NC: 0.14%; PT‐A: 0.23%; PT‐Ac: 0.25%), *Gemella* (PT‐NC: 0.13%; PT‐A: 0.64%; PT‐Ac: 0.27%) and *Parvimonas* (PT‐NC: 0.019%; PT‐A: 0.09%; PT‐Ac: 0.010%).

### 3.6 Metatranscriptomics of tumor tissues

In order to evaluate bacterial activity in the tumor tissue of P89 where *Parvimonas* was isolated, a metatranscriptomic analysis was performed from colon tissues (Fig. 2). In particular, the adenocarcinoma tissue (T89‐FT‐Ac), as well as non‐neoplastic colon mucosa tissue (T89‐FT‐NC) and transition tissue (interface between non‐neoplastic and adenocarcinoma region, T89‐FT‐Tr) were analyzed.

**Fig. 9:**
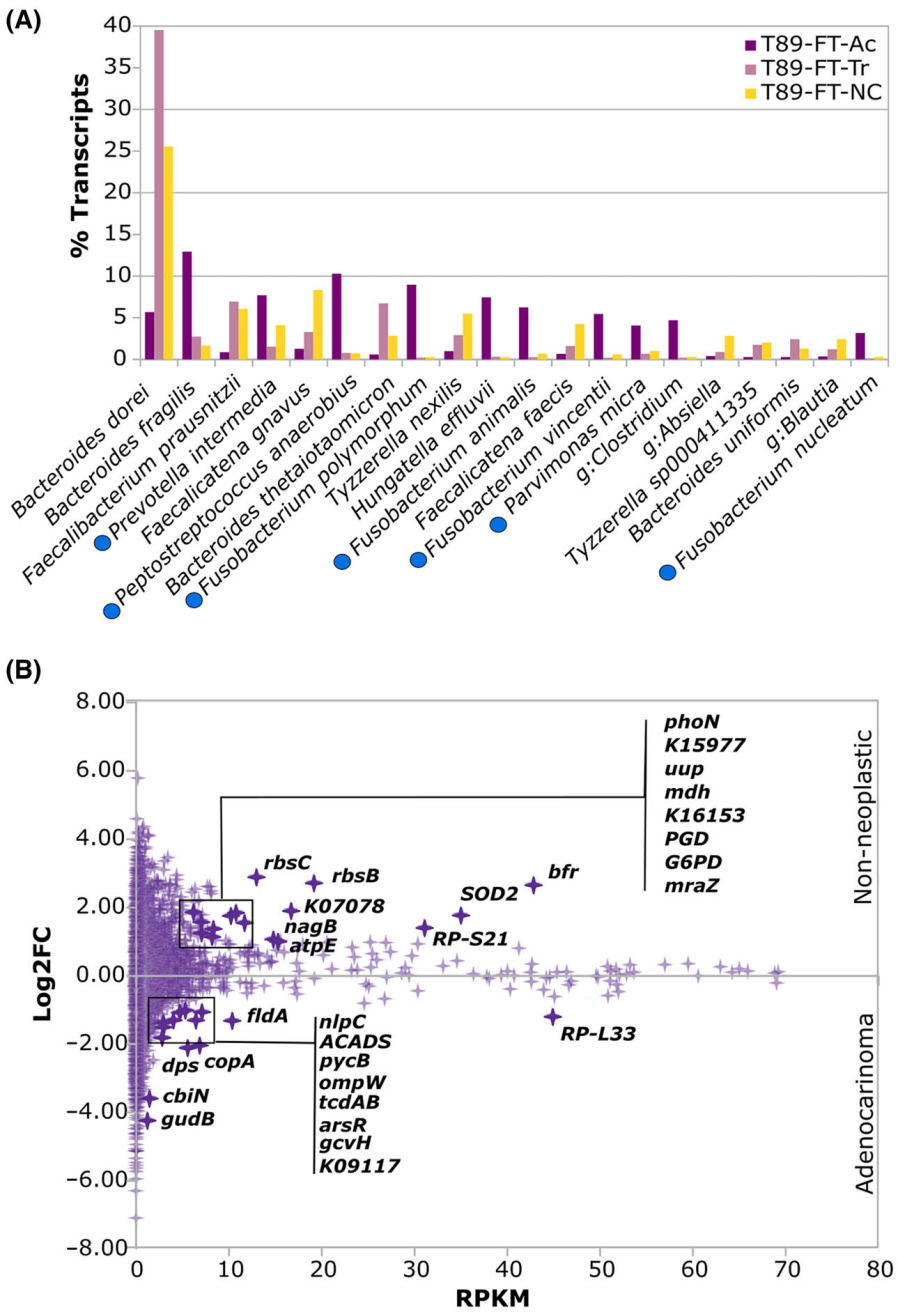
Metatranscriptome of CRC patient 89 (P89) obtained by RNAseq of tissues. (A) Percentage of transcripts assigned to bacteria at species level: T89‐FT‐Ac (adenocarcinoma tissue), T89‐FT‐Tr (transition tissue), T89‐FT‐NC (non‐neoplastic colon tissue). Blue dots indicate oral bacteria. (B) Active genes from the microbial community in non‐neoplastic tissue (T89‐FT‐NC) vs adenocarcinoma (T89‐FT‐Ac). Data show the abundance in non‐neoplastic (RPKM: Number of reads normalized by length of the gene (Kb) and size of the dataset (Mb)) and log2Fold change (non‐neoplastic/adenocarcinoma) for each gene. Marked genes have log2FC >1 and mean RPKM > 5.

**Fig. 10:**
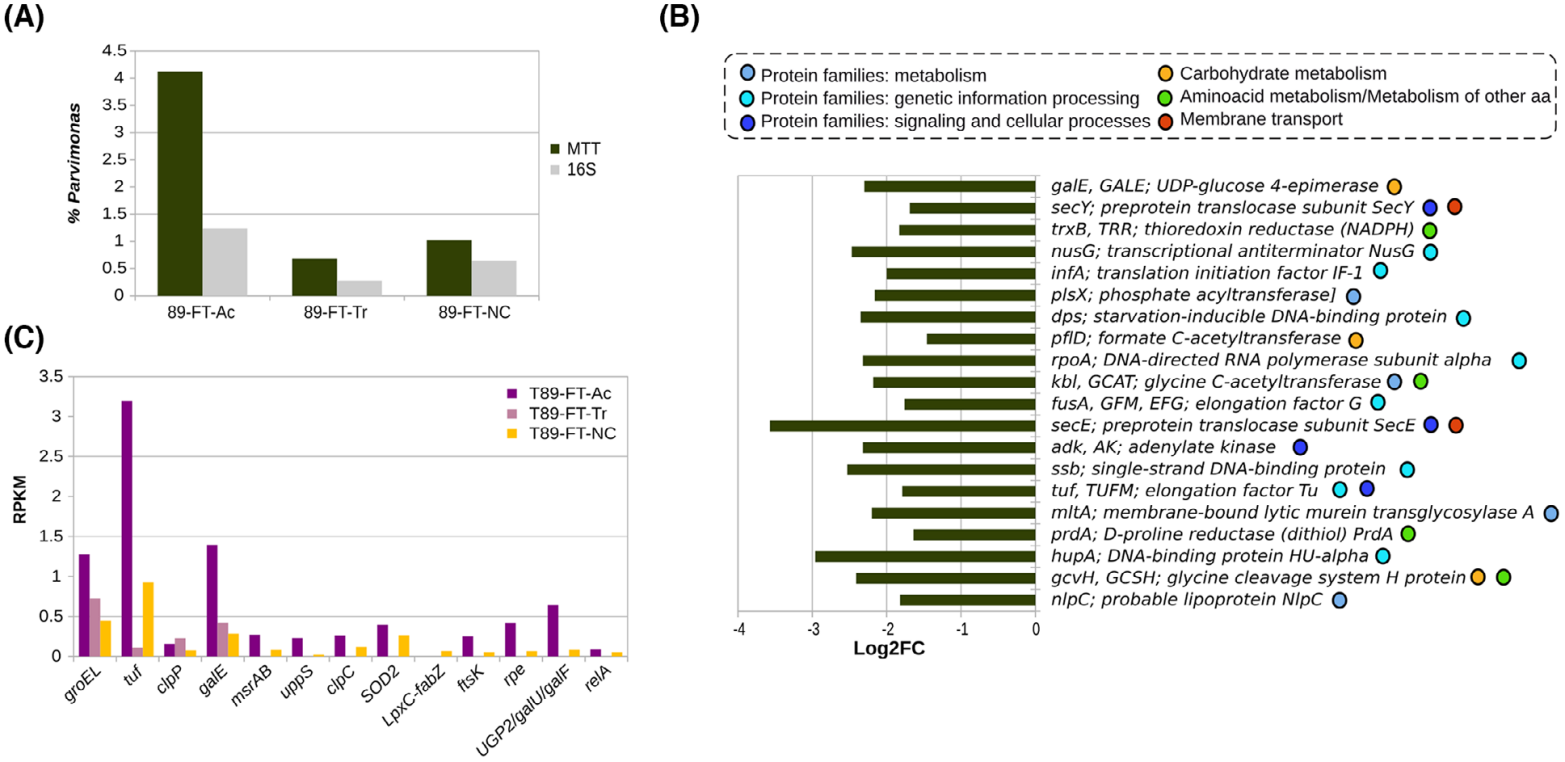
*Parvimonas micra* activity in CRC patient 89 (P89). (A) RA of *P. micra* as indicated by 16S rRNA gene sequencing (M89‐FT) and by the corresponding percentage of transcripts in the metatranscriptome (MTT, T89‐FT) of adenocarcinoma samples. (B) Top 20 genes (excluding ribosome proteins) expressed by *P. micra* in adenocarcinoma. Data show the log2Fold change (non‐neoplastic/adenocarcinoma). Color dots indicate the KEGG subcategory of each gene. (C) *P. micra* expression of genes identified as potential virulence factors for adenocarcinoma (Ac), transition (Tr) and non‐neoplastic colon (NC) tissues.

At the species taxonomic level, a higher richness and diversity were observed in the non‐neoplastic tissue compared to the adenocarcinoma sample (Table S7 in the supporting information section). Microbial activity profile was different at the species level when these samples were compared (Fig. 9A). The active microbiota in the non‐neoplastic colon tissue (T89‐FT‐NC) and colon transition tissue (T89‐FT‐Tr) from P89 was dominated by *B. dorei* (FT‐NC: 25.55%, FT‐Tr: 39.53%, FT‐Ac: 5.68%), *Faecalibacterium prausnitzii* (FT‐NC: 6.06%, FT‐Tr: 6.94%, FT‐Ac: 0.87%), *Faecalicatena gnavus* (FT‐NC: 8.35%, FT‐Tr: 3.29%, FT‐Ac: 1.27%) and *Bacteroides thetaiotaomicron* (FT‐NC: 2.83%, FT‐Tr: 6.72%, FT‐Ac: 0.59%). In contrast, *B. fragilis* (FT‐N: 1.65%, FT‐Tr: 2.74%, FT‐Ac: 12.94%), *Peptostreptococcus anaerobius* (FT‐NC: 0.73%, FT‐Tr: 0.78%, FT‐Ac: 10.29%) *and Fusobacterium polymorphum* (FT‐N:0.3%, FT‐Tr: 0.23%, FT‐Ac: 8.96%) dominated the adenocarcinoma tissue. In addition, other oral associated species like *Fusobacterium* species, *P. intermedia* and *P. micra* also accounted for a higher percentage of activity in adenocarcinoma than in the non‐neoplastic tissue. *Parvimonas* was detected by 16S rRNA gene sequencing and also metatranscriptomic analysis in the three different tissues. Moreover, *Parvimonas*, represented a higher ratio of percentage of transcripts (MTT) vs the RA in 16S rRNA metabarcoding (16S) (MTT/16S: 1.58 in T89‐FT‐NC, 2.5 in T89‐FT‐Tr and 3.33 in T89‐FT‐Ac) in the adenocarcinoma tissue than in the other tissues (Fig. 10A).

Since no replicates were obtained, no statistical tests could be performed. Therefore, a gene was considered to be over‐expressed when it had a mean abundance (RPKM) higher than 5 and a difference in abundance (log2[foldchange]; log2FC) higher than 1. According to these criteria, gene expression profiles comparison revealed 16 genes that were over‐expressed in non‐neoplastic tissue and 14 genes in the adenocarcinoma (Fig. 9B).

Interestingly, among those genes over‐expressed in the adenocarcinoma we found some stress indicators such as *dps*; starvation‐inducible DNA‐binding protein or *arsR* transcriptional regulator (implicated in ion homeostasis, biofilm formation, primary and secondary metabolism, response to adverse condition, and virulence). In addition, increased expression of *ompW*, an outer membrane protein which acts as a receptor for colicin S4 (colicins are plasmid‐encoded toxic proteins produced by *Escherichia coli* strains), or the tcdAB, toxin A/B (pro‐inflammatory and cytotoxic, causing disruption of the actin cytoskeleton and impairment of tight junctions in human intestine).

Other genes related with metabolism like different metal transporters (*copA*, Cu + −exporting ATPase; *cbiN*, cobalt/nickel transport protein), enzymes involved in carbon metabolism (*ACADS*, butyryl‐CoA dehydrogenase; *pycB*, pyruvate carboxylase subunit B; *gcvH*, glycine cleavage system H protein), and a glutamate dehydrogenase (*gudB*), that allows the use of glutamate as a carbon source, were also more expressed in adenocarcinoma. Meanwhile, in non‐neoplastic tissue other several genes were over‐expressed coding for proteins involved in carbon metabolism (*mdh*, malate dehydrogenase; *PGD*, 6‐phosphogluconate dehydrogenase; *G6PD*, glucose‐6‐phosphate 1‐dehydrogenase), in amino‐sugar metabolism (*nagB*), as well as two subunits of ribose transporter (*rbsB*, *rbsC*), *atpE* a F‐type H^+^‐transporting ATPase (used by aerobic organisms for synthesizing ATP) and *SOD2* superoxide dismutase (which neutralizes toxic levels of reactive oxygen species).

Focusing on the transcriptional profile of *Parvimonas* of P89, a total of 808 different KEGG genes were assigned. The ones with higher expression were genes that codifies for several ribosome proteins, DNA replication proteins (*hupA*, *ssb*) transcription machinery (*rpoA*, *nusG*) and translation factors (*tuf*, *fusA*, *infA*) confirming that *Parvimonas* was transcriptionally active (Fig. 10B). Genes related to the metabolism of carbohydrates (*gcvH*, *pflD*, *galE*), amino acids (*prdA*, *kbl*, *trxB*) and proteins (*nlpC*, *mltA*, *plsX*) were also found. In fact, the most expressed gene by *P. micra* in adenocarcinoma was a probable lipoprotein (*nlpC*) that was also among the more overexpressed in this tissue globally. Further annotation of this gene with Pyre2 showed that only the last part of the protein (119 residues) was similar to the putative cell wall hydrolase (autolysin acd24020 catalytic domain, that belongs to the NlpC/P60 family) from *Clostridium difficile* (Table S8).

Regarding the potential virulence factors detected in the genomic analysis of isolates, we found transcripts of 13 out of 25 genes identified. All of them were more expressed in adenocarcinoma with the exception of *clpP* (more expressed in transition tissue) and *LpxC*‐*fabZ* (more expressed in non‐neoplastic tissue) (Fig. 10C).

## 4 Discussion

A previous and wider work done by our team using the cohort of 93 CRC patients and 30 healthy controls revealed that the abundance of several periodontal pathogens in the oral cavity as well as in colon samples was much higher in CRC patients than in healthy controls. In the present work, we focused on *P. micra*, which was significantly enriched in fecal samples from CRC patients compared to those of healthy individuals. In fact, culturomic approaches allowed us to isolate *P. micra* from gingival crevicular fluid and adenocarcinoma samples from CRC patients, and the corresponding bacterial genomes were fully sequenced. Even though culturomic procedures were done for a big set of CRC diagnosed patients, *P. micra* isolates were only successfully isolated from five CRC patients and not from all types of samples, probably due to the difficulties derived from sample collection during surgery or dental exploration and the fastidious nature of this microbe.

Whole genome analyses on gingival and adenocarcinoma P89 isolates evidenced that gingival‐adenocarcinoma pairs were > 99% identical at nucleotide level and had a high degree of synteny. Interestingly, two prophages were detected in all PM89KC isolates. These prophages have been also found in other *Parvimonas* isolates, located in different places across the genome, although usually not duplicated. Furthermore, the acquisition of prophages can also prevent invasion by other phages or give the microorganisms some advantages (higher virulence, major adhesion or biofilm formation ability) [67]. Additionally, the adenocarcinoma isolate has an extra CRISPR array and more spacers when compared to the subgingival isolates. Spacers contain pieces of phages and are used by the CRISPR‐Cas system as a defense mechanism against these organisms. Two of the new spacers in PM89KC‐AC‐1 showed high similarity to unidentified phages in the genera *Coprococcus* and *Tyzzerella* (both from the family *Lachnospiraceae*) in the GPD. There are 8 spacers with 100% identity between all P89 isolates, which would be congruent with a putative common origin. The high ANI values obtained, the high synteny observed and the presence of a same type III‐B CRISPR‐Cas system with some shared spacers, together with the same duplicated prophages in paired PM89KC isolates, strongly suggest that both gingival and adenocarcinoma isolates have a recent common ancestor, and that *P. micra* seems to be able to translocate from the periodontal pockets to other locations such as the gut. In the colon, a great abundance of different phages [68] could have induced the increase of CRISPR‐Cas defenses, enlarging the number of spacers observed in the PM89KC‐AC‐1 isolate. Additionally, it is possible that the duplication of the prophage could provide new advantages to *P. micra* in the gut, as several studies in other microbes have reported for effects in biofilm formation or defense against other viruses. For example, several virulence factors were detected in the PM89KC prophages including a Type IV secretion system and some of its associated proteins like PcfB and PrgI [69]. Other relevant proteins found were the NlpC/P60 family protein in the left prophage which contributes to cell wall remodeling, and a CHAP domain containing protein in the right phage which is related to virulence [70].

Therefore, our results support that *P. micra*, a strictly anaerobic bacterium commonly found in the oral cavity, and over‐abundant in patients with periodontal diseases [9, 11], could have migrated from the subgingival pocket to the colon. Similar results were also observed by using an animal model approach for the periodontal pathogen *F. nucleatum* [17].

Regarding P79, where *P. micra* was also isolated in gingival and tumor samples, paired isolates obtained showed a lower percentage of identity than isolates from P79. This could indicate that PM79KC‐AC and PM79KC‐G isolates obtained from P79 could be indeed different strains or that these isolates from P79 have a common origin but the translocation event took place longer time ago, leading to a higher sequence divergence.

When fastidious anaerobic bacteria, such as *Parvimonas* or other periodontal pathogens, arrive to the gut, they can settle at the base of villi or in intestinal crypts of Lieberkühn as well as in dysplastic structures of adenomas or adenocarcinomas where the oxygen pressure is low. Consequently, the original oral *P. micra* could undergo different genomic rearrangements, accumulate mutations and promote changes in gene expression to adapt to its new niche such as those observed in this study. One of the most apparent genomic changes include the deletion or inactivation of different genes, such as several transporters, as well as the increase of insertion elements, which are both typical features of bacterial adaptation to a new niche [71].

Periodontitis is a chronic inflammatory disease caused by a multispecies community of periodontal pathogens affecting dental supporting tissues. Although bacterial plaque is the primary etiologic factor, progression and clinical characteristics of these diseases are influenced by acquired, local, systemic, and genetic factors that can modify susceptibility to this poly‐microbial infection [72]. In the concrete case of P89, the oral health assessment performed in this work by a dentist allowed us to know that this patient presented a periodontal disease in stage IVB. In the preliminary interview, P89 reported several oral surgeries in recent years, as a consequence of her periodontal disorders. Previous studies have proposed 3 potential pathways for oral microbes to reach the gut niche, namely: (A) as a consequence of a transient bacteremia during dental procedures, such as tooth extraction, dental cleaning or different oral surgeries [73, 74]; (B) as a consequence of the direct contact between the subgingival biofilm and the blood vessels in the deep pockets of patients with periodontitis [17, 73, 75, 76, 77, 78]; or (C) through the oral‐gut axis during meals or during daily swallows of saliva [79, 80]. It is important to note that some oral microorganisms, such as *P. gingivalis* or *F. nucleatum*, can penetrate and invade eukaryotic cells (especially macrophages, dendritic or epithelial cells of the gums), using them as a “refuge” to avoid the effects of the host's immune system migrating without being perceived to other regions through the circulatory system or through the GI tract [81]. Therefore, *P. micra* PM89KC translocation from the oral cavity to the colon could have been facilitated during P89 oral surgeries or during bleeding caused through daily dental cleaning, due to her advanced stage of periodontal disease.

Multiple studies supported that a bad periodontal health was related to other systemic diseases but also to certain types of cancer [1, 4, 12, 17, 19]. It is increasingly being proven that the dissemination of pathobionts from the oral cavity to distal areas of the human body implies that these bacteria can exert harmful functions on human cells by colonizing these new ecological niches. In 2020, a study demonstrated in a mouse model that *F. nucleatum*, reached colorectal tumors coming from the oral cavity via intravenous route [17]. In addition, authors isolated different *F. nucleatum* strains from saliva and adenocarcinoma of CRC patients, demonstrating that fusobacteria found in carcinomas migrated from the oral cavity [17] as we have proposed for *P. micra* in the present study.

Several genomic features of *P. micra*, like the small genome size or the high A + T content, suggest that this species could have an intracellular lifestyle [58, 59]. Thus, *P. micra* could be able to migrate from the oral cavity to other localizations of the human body inside epithelial cells avoiding the human immune system as previously suggested [57]. Besides, it is interesting to note that nine genes related to cell membrane transport were lost by the adenocarcinoma isolate (PM89KC‐AC) in comparison to gingival isolates. This genomic reduction process has been, linked to bacteria changing their original ecological niche, supporting the hypothesis that *P. micra* could be intracellular [71, 82].

Furthermore, oral, gut, and carcinoma microbiota of P89 was analyzed by 16S rRNA sequencing using samples of different nature. The analysis of adenocarcinoma and oral samples performed in the current study showed a clear over‐representation of oral bacteria in CRC tissue, as reported in previous studies [19, 83]. Additionally, we analyzed and compared the transcriptomic profile of *P. micra* in the control, non‐neoplastic region vs the colon adenocarcinoma with the aim of identifying genes that could be related to cancer development and progression. Two lines of evidence support that the detected DNA correspond to active, viable oral pathogen bacteria. Firstly, bacterial isolates of *P. micra* were obtained from fresh tumor samples, and secondly, the metatranscriptomic analysis of the adenocarcinoma sample confirmed that these bacteria are not only transcriptionally active but also at higher levels in the RNA pool than on the DNA‐based analysis. Moreover, it was found that many of the bacteria which are more active in the tumor are oral pathogens. Taking the 16S rRNA metabarcoding and the metatranscriptomic analysis into account, we suggest that *P. micra* could arrive to the gut accompanied by other oral pathobionts, such as *Fusobacterium*, through the formation of polymicrobial aggregates. Micro‐communities of bacteria are naturally found in human saliva and are composed by both aerobic and anaerobic microbes [84]. These bacterial aggregates appear to be able to grow and form biofilms more efficiently than if they travel as individual sessile cells [84]. Metabolic interactions and co‐operation between different genera could increase survival of fastidious bacteria, such as *P. micra*, during these translocation processes [85]. This, together with the fact that oral bacteria were found as transcriptionally active in adenocarcinoma could explain why different subgingival pathogens were detected in adenoma and adenocarcinoma samples through 16S rRNA metabarcoding bioanalysis. It has to be born in mind that these oral microbes could promote a gut inflammatory effect, individually, as reported previously for *P. micra* [86] and for other bacteria [4, 87, 88] or synergistically when living in communities or aggregates [89].

Specifically, different adhesins of *F. nucleatum*, such as Fap2, FadA, RadD and CmpA, have shown to play an important role in bacterial aggregation abilities, both in the gingival and gut environment, as well as in their adhesion to the carcinoma tissue [90]. The most important protumorigenic mechanism of *F. nucleatum* is based on the FadA ability to increase the β‐catenin/WNT signaling pathway and the annexin A upregulation, which promotes cell proliferation [91, 92, 93]. *F. nucleatum* Fap2 adhesin can also impair immune host functions and activate epithelial and myeloid cells in the colon, reducing cytotoxicity and promoting a pro‐inflammatory status in the gut, respectively [94, 95, 96]. Another oral pathogen found to be quite active in the P89 tumor sample was *P. anaerobiu*s, which has also been linked to the development of CRC in recent years. This bacterium can interact with toll‐like receptors of colon cells, such as TLR‐2 and TLR‐4, modulating the activity of immune cells and increasing ROS production [97, 98]. It has also been described that the binding of *P. anaerobius* to tumor cells is due to the interaction with α2/β1 integrins that activates the PI3K/AKT cell signaling pathway, stimulating epithelial inflammation and hyperproliferation of the colon cells [97]. Besides, *P. intermedia*, an oral pathogen involved in the pathogenesis of periodontitis and detected in the tumor tissue of P89, seems to play a crucial role in CRC progression, lymph node affectation and distant metastasis. Additive pro‐tumoral effects were observed in CRC cell lines when *P. intermedia* was combined with *F. nucleatum* due to the capacity of these two bacteria to metabolize glucose into formate, a well‐known oncometabolite [99]. Moreover, *P. intermedia* produces 6‐phosphate isomerase, an autocrine motility component that stimulates tumoral invasion [99].

Focusing only on *P. micra* results, transcriptomic bioanalysis confirmed that the PM89KC strain was more active in the adenocarcinoma than in the non‐neoplastic distant tissue, supporting that this pathobiont is not only present in dysplastic tissues (revealed by 16S rRNA metabarcoding analysis) but also viable (cultured at the laboratory) and active (demonstrated by metatranscriptomic analysis), so its potential role in cancer development should be further explored. Remarkably, the gene most expressed by *P. micra* in the adenocarcinoma was a probable lipoprotein *nlpC* that was also among the more overexpressed in the carcinoma tissue globally. NlpC/P60 domains are bacterial peptidoglycan hydrolases that cleave noncanonical peptide linkages and contribute to cell wall remodeling [100], and its potential role in the adenocarcinoma tissue should be further explored. In addition, another highly expressed gene from *Parvimonas* was *mltA* (membrane‐bound lytic murein transglycosylase A), which also degrades murein. This suggests that *Parvimonas* could be remodeling its peptidoglycan to facilitate cell growth, or it could be also a strategy of pathogenesis to resist human degradative enzymes or release of cytotoxic muropeptides.

Among the potential virulence effects of *P. micra*, its proteolytic potential could be relevant. Previous studies reported that endogenous proteolytic activity of *P. micra* facilitates bacteria dissemination into periodontal tissues but also to blood vessels [101]. Furthermore, this pathobiont can regulate and activate the proteolytic activity of other key oral pathogens (belonging to the red and orange complexes). For example, an *in vitro* study showed that *P. micra* stimulates the biosynthesis of proteolytic gingipains, enhances the growth of and coaggregates with the well‐known pathogen *P. gingivalis* [11]. Moreover, *P. micra* showed synergic biofilm formation and coaggregation with *F. nucleatum*, another periodontal pathogen associated with CRC [9]. In fact, several studies suggested that these two pathobionts found in CRC tumors could be strongly associated and correlated with survival prognosis of patients [13, 14, 102]. An *in vivo* research work showed that *P. micra* has pathogenic synergy with *P. intermedia* and *Prevotella nigrescens*, showing higher transmissibility of infection, and also enhanced *Prevotella* growth and oral abscesses aggravation [103]. Furthermore, another study demonstrated that *P. micra* coaggregates with another well‐characterized oral pathogen: *Treponema denticola* [104].

The over‐abundance of *P. micra* was linked in the last years with colorectal carcinomas showing a high infiltration degree of immune cells such as CD8+ cytotoxic T, CD4+ T helper and NK cells lymphocytes [15]. *P. micra* was also shown to shoot up carcinogenesis *in vitro* over colon cell lines such as NCM460, HT29 and Caco2, and *in vivo*, in ApcMin/+ and germ‐free mice, increasing the expression of pro‐inflammatory cytokines and promoting the proliferation of colon cells [15]. Experiments performed by Zhao et al. [15] demonstrated that *P. micra* modified the immune response of the host, increasing the biosynthesis of pro‐inflammatory interleukins (IL‐17, IL‐22 and IL‐23a) and the response of the lymphocytes Th‐17. Bergsten et al. [16] proved that *P. micra* causes hypermethylations in the promoters of genes related to the cytoskeleton, such as *SCIN* and *DIAPH3*, in tumor suppressor genes, such as *TSPAN13*, *HACE1*, *SEMA3F* and *SASH1*, and in epithelial‐mesenchymal transition genes such *FBXO32*, in colon cells. A recent study also demonstrated that these periodontal bacteria can also promote CRC development increasing the expression of a specific microRNA (miR‐218‐5p) in cells and exosomes, inhibiting the expression of *PTPRR* and enhancing the RAS/ERK/C‐FOS signaling pathway, which leads to colon cells overgrowth [105]. It is important to note that Zhao et al. [15] concluded that *P. micra* could act as a poor survival biomarker in CRC patients, after the analysis of the association of its abundance in fecal samples with clinical data. Overall data indicate that *P. micra* could promote carcinogenesis processes through different pathways: a) increasing the secretion of pro‐inflammatory and protumoral molecules, b) promoting lymphocytes and leukocytes recruitment in the carcinoma microenvironment and c) causing aberrant DNA hypermethylation in the colorectal cells and modifying the gene expression patterns.

Besides, in the present study, we detected the presence of *Parvimonas* DNA, among other periodontal pathogens in liver tissues of P89. A recent study also reported the presence of typical gut microbes in liver samples of CRC patients that underwent liver metastasis, suggesting that a pre‐metastatic niche may be constructed by gut bacteria, allowing tumoral cells to develop secondary tumors in the liver [87]. While it is true that *Parvimonas* was not detected by 16S rRNA metabarcoding in neoplastic liver tissues, *Parvimonas* among other periodontal pathogens were present in the non‐neoplastic region of the liver tissue. There, *P. micra* and other pathobionts could promote liver inflammation by disrupting the normal function of the NOD2 signaling pathway [10]. However, it has to be highlighted that the methodology applied has serious limitations for small samples. Therefore the presence of *P. micra* in the metastatic liver tissue cannot be totally discarded. Additionally, it was previously reported that periodontal pathogens could arrive from the colon to the liver via circulatory system (by the portal vein) [87, 106, 107, 108, 109].

In general, the RA of Parvimonas is low in most samples studied. It has been previously described that some pathogens, commonly called *keystone* bacteria, can promote harmful effects in the host even if they are in low abundance within the community. For example, *P. gingivalis*, despite being a minority member of the community induces changes in the RAs of other oral bacteria, promoting chronic oral inflammation [110]. Yachida et al. [111] reported that *P. micra* is involved in the carcinogenesis course despite its negligible RA when compared with other overabundant bacteria detected in colorectal tumors such as Bacteroides. Accordingly, it can be hypothesized that *P. micra* is capable of colonizing the dysbiotic colon at early stages helping to create an adequate tumor microenvironment and promoting the colonization of other opportunistic bacteria.

There are several factors that can affect the composition of the gut and oral microbiota and, therefore, the detection of periodontal pathogens such as *P. micra* in the samples. These factors are age, sex, diet, lifestyle, and drugs, among others. Lifestyle, drug consumption, or diets have been used as inclusion/exclusion criteria for recruiting patients. Overall analysis revealed that the abundance of *P. micra* did not depend on age or sex. Considering that *P. micra* was associated with CRC [7, 12, 112] and taking into account our results, we propose that *P. micra* may promote pro‐tumoral colon inflammation and/or adenocarcinoma development in susceptible patients. The tumors appear to be a stressful environment (as shown by the over‐expression of stress response genes in the tumor metatranscriptome) where bacteria produce toxins among different virulence factors. The potential role of these anaerobic bacteria to generate an inflammatory microenvironment and in the initiation or progression of the tumor should be investigated in‐depth. More *in vitro* and *in vivo* studies are needed to elucidate the cell mechanisms of *P. micra* in cancer development, since its pathogenicity remains unclear. The specificity in the detection of *P. micra* in fecal samples from our cohort was 96.67%, demonstrating the scarce presence of this bacterium in the intestine of healthy individuals. For this reason, we consider that *P. micra* could be considered as a CRC biomarker detected in non‐invasive samples such as saliva or feces. Other authors [113, 114] reported a specificity in the detection of *F. nucleatum* and *Lachnoclostridium* sp of 76% and 78.5%, respectively. However, these authors used PCR strategies instead of NGS sequencing techniques, so our results cannot be compared with theirs.

Most of the CRC common symptoms are nonspecific, appearing when the tumor is at advanced stages. Later CRC detection decreases the survival rate and increases the morbidity of patients. In Spain, intensive CRC screening programs are carried out in 50–69 years old people, with the aim of early detecting asymptomatic colorectal carcinomas by monitoring the presence of blood occult in feces (FOBT). Particularly, in Galicia (NW Spain), the CRC screening program incorporates a colonoscopy for all people with positive results in FOBT. Colonoscopy may find CRC signs, however, it is an invasive test that causes discomfort to the patients, being unnecessary in most cases. Therefore, new non‐invasive and early biomarkers of CRC are needed. Since gut dysbiosis occurs at early stages of the adenoma‐carcinoma sequence, we propose that the complementation of FOBT with a bacteriome test could increase the number of diagnoses of CRC at early stages. This bacteriome test should include the detection of *P. micra*, that showed a specificity of 96.67%, but also the detection of other well‐known periodontal pathogens such as *F. nucleatum*, in fecal samples.

## 5 Conclusions

The main finding of the present work suggests that *P. micra* is able to translocate, possibly aggregated with other oral pathobionts, such as *Fusobacterium*, from the subgingival sulcus of the oral cavity to the colon, possibly via the circulatory system or the oral‐gut axis. In this new niche, *P. micra* has to adapt to survive, undergoing genomic and transcriptomic rearrangements as well as new expression patterns. The findings of the present study support the relation between periodontal pathogens and the development of CRC. This may be of great importance and demonstrates that proper oral health maintenance and an early detection of periodontal diseases could reduce the risk of CRC. Finally, we suggest that *P. micra* can be an interesting CRC biomarker.

## Conflict of interest

The authors declare no conflict of interest.

## Author contributions

JAV, MP, AM, SL, and GB conceived and designed the study. MP, JAV, and AM supervised the study. KC‐P, JAV, and MP obtained fecal and saliva samples. MP managed the informed consent from patients. JFN obtained fresh tissue samples from surgery. LSE, BO‐A, and ÁC collected and studied FFPE samples. KC‐P, NT‐T, MN‐A, JAV, and SR‐F processed samples and performed 16S rRNA metabarcoding and whole genome sequencing. EB, MC‐D, and AM performed transcriptomic analysis. PA‐M, EM‐DA, II‐C, KC‐P, JAV, and SL made bioinformatics analysis related to DNA sequencing. KC‐P, PA‐M, EB, and EM‐DA designed and created tables, Figs 1, 2, 3, 4, 5, 6, 7, 8, 9, additional files and graphical abstract of the manuscript. SP‐L obtained subgingival samples and made dental checkups for patients. IG‐R, NM‐L and LAA (Deceased) followed up oncological patients. KC‐P, JAV, MP, MC‐D, AM, EB, PA‐M, ÁC, BO‐A, SP‐L, EM‐DA, IG‐R, NM‐L and SL wrote the main manuscript text. All authors reviewed the manuscript.

### Peer review

The peer review history for this article is available at https://www.webofscience.com/api/gateway/wos/peer‐review/10.1002/1878‐0261.13506.

## Supporting information


**Fig. S1.** Abundance of *Parvimonas* (above) and density distribution of samples (below) depending on sex, age and group of samples (CRC and non‐CRC).
**Fig. S2.** Heatmap showing Average Nucleotide Identity values as obtained by orthology (OrthoANI) between the *P. micra* genomes isolated in this study and others available at the NCBI database.
**Fig. S3.** Graphic scheme of CRISPR‐Cas sequences disposition in the genome of KCOM 1037 strain (used as reference) vs CRISPR‐Cas system and prophage sequences arrangement in the *P. micra* PM89KC isolates genomes.
**Table S1.** Differential abundance analysis (DAA) of *Parvimonas*, *Fusobacterium* and *Peptrostreptococcus* in stool samples between CRC and healthy subjects (98 CRC patients and 30 healthy controls) using ANCOM‐BC at genus level, with a prevalence cut of 0.1 and adjusting the *P*‐values by the Holm‐Bonferroni method.
**Table S2**. Loci affected by non‐synonymous mutations detected in the adenocarcinoma *P. micra* PM89KC‐AC‐1 strain, using the gingival PM89KC‐G‐1/2 strains as reference.
**Table S3.** Comparison of genes identified in the cross‐shaped structure found in the *P. micra* PM89KC‐AC‐1 isolate, composed of two prophages.
**Table S4.** CRISPR‐Cas systems found on *P. micra* analyzed genomes, with their CRISPR arrays, spacer counts and consensus repeats. The consensus repeats have been switched to match orientation in all genomes. In small CRISPR arrays (i.e. PM89KC‐G‐1 A). For isolates with very high identity (EYE group or PM89KC‐AC isolates 1–4) only one of the isolates was analyzed.
**Table S5.** Virulence factors present in *P. micra* strain PM89KC‐AC‐1, using DIAMOND against the Virulence Factor Database (version 2021‐10‐04).
**Table S6.** Virulence factors present in *P. micra* PM89KC‐G‐1 isolate, using DIAMOND against the Virulence Factor Database (version 2021‐10‐04).
**Table S7.** Diversity and richness in metatranscriptome analysis at species level.
**Table S8.** Identification and re‐annotation of top 20 most expressed genes by *P. micra* in adenocarcinoma tissue in PM89KC‐AC‐1.

## Data Availability

Genomes from each *P. micra* isolate sequenced in this study were deposited in the National Center for Biotechnology Information (NCBI) Sequence Read Archive (SRA). GenBank Assembly accession codes for each *P. micra* isolate are shown in Table 1 (Bioproject code: PRJNA11189). All data obtained from 16S rRNA gene sequencing and from transcriptomic analyses are also available at NCBI SRA database under the accession codes PRJNA911189 and PRJNA893853, respectively. All genomic and microbiomic scripts are available in GitHub (https://github.com/Pablo‐Aja‐Macaya/parvimonas‐travel‐study).
