# Supplementary material for: Parvimonas micra can translocate from the subgingival sulcus of the human oral cavity to colorectal adenocarcinoma
Source: Mol Oncol. 2023 Sep 13;18(5):1143–73. doi: 10.1002/1878-0261.13506 (PMC11076991; doi:10.1002/1878-0261.13506)
Supplement: Supplementary file 1 — Fig. S1. Abundance of Parvimonas (above) and density distribution of samples (below) depending on sex, age and group of samples (CRC and non‐CRC). Fig. S2. Heatmap showing Average Nucleotide Identity values as obtained by orthology (OrthoANI) between the P. micra genomes isolated in this study and others available at the NCBI database. Fig. S3. Graphic scheme of CRISPR‐Cas sequences disposition in the genome of KCOM 1037 strain (used as reference) vs CRISPR‐Cas system and prophage sequences arrangement in the P. micra PM89KC isolates genomes. Table S1. Differential abundance analysis (DAA) of Parvimonas, Fusobacterium and Peptrostreptococcus in stool samples between CRC and healthy subjects (98 CRC patients and 30 healthy controls) using ANCOM‐BC at genus level, with a prevalence cut of 0.1 and adjusting the P‐values by the Holm‐Bonferroni method. Table S2. Loci affected by non‐synonymous mutations detected in the adenocarcinoma P. micra PM89KC‐AC‐1 strain, using the gingival PM89KC‐G‐1/2 strains as reference. Table S3. Comparison of genes identified in the cross‐shaped structure found in the P. micra PM89KC‐AC‐1 isolate, composed of two prophages. Table S4. CRISPR‐Cas systems found on P. micra analyzed genomes, with their CRISPR arrays, spacer counts and consensus repeats. The consensus repeats have been switched to match orientation in all genomes. In small CRISPR arrays (i.e. PM89KC‐G‐1 A). For isolates with very high identity (EYE group or PM89KC‐AC isolates 1–4) only one of the isolates was analyzed. Table S5. Virulence factors present in P. micra strain PM89KC‐AC‐1, using DIAMOND against the Virulence Factor Database (version 2021‐10‐04). Table S6. Virulence factors present in P. micra PM89KC‐G‐1 isolate, using DIAMOND against the Virulence Factor Database (version 2021‐10‐04). Table S7. Diversity and richness in metatranscriptome analysis at species level. Table S8. Identification and re‐annotation of top 20 most expressed genes by P. micra in adenocarcinoma [file MOL2-18-1143-s001.docx]

**SUPPORTING INFORMATION**

**SUPPLEMENTARY FIGURES**

**
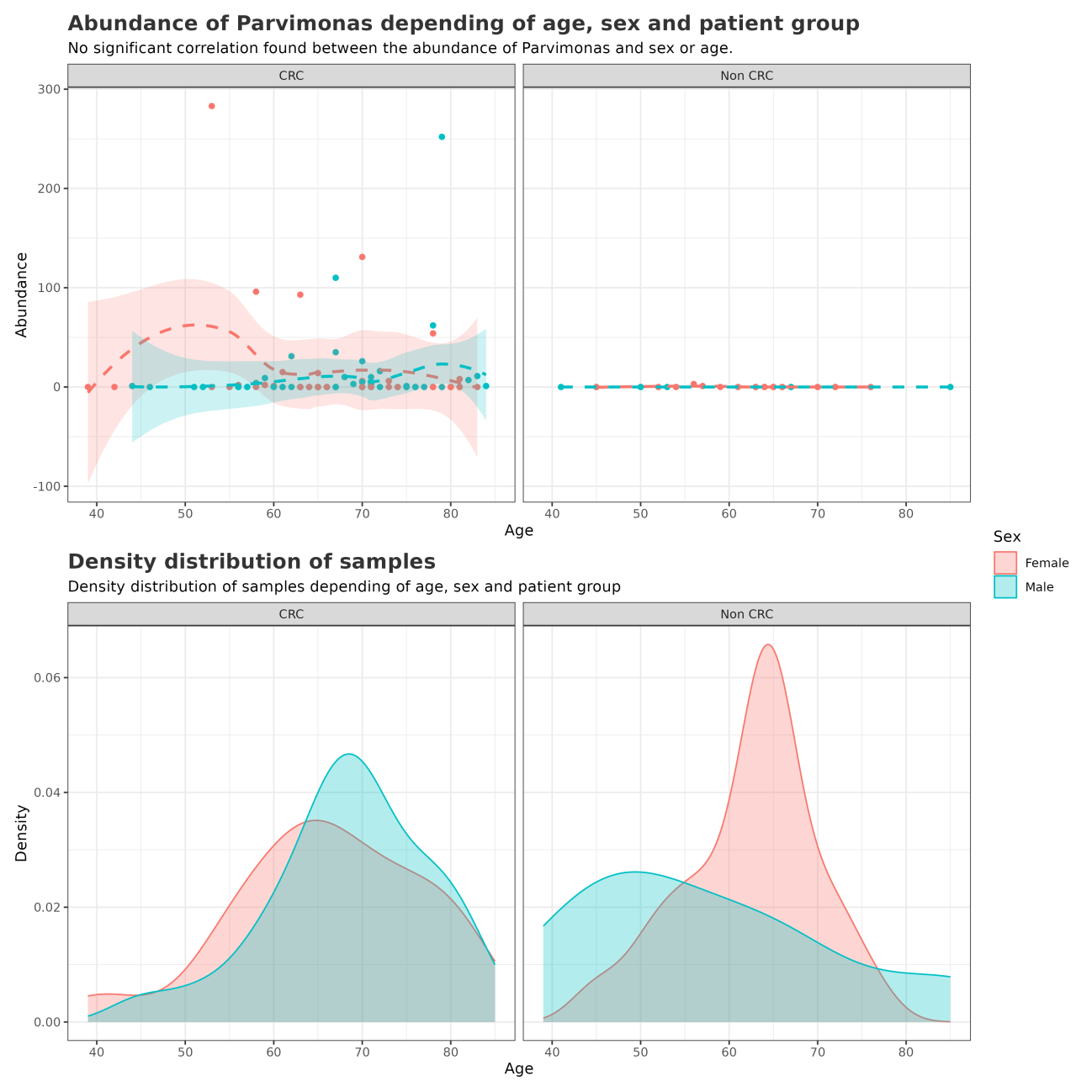
**

**Figure S1.** Abundance of Parvimonas (above) and density distribution of samples (below) depending on sex, age and group of samples (CRC and non-CRC). Samples were rarified to 10,000 counts and a loess model was fit to see the distribution of the abundance across group, age and sex. A Kruskal-Wallis rank sum test was performed on the CRC patients, resulting in no significant differences in Parvimonas abundance when grouping by sex and age group (p=0.84) or just by sex (p=0.886). A significant difference was found when grouping by CRC and non-CRC (p<0.001).


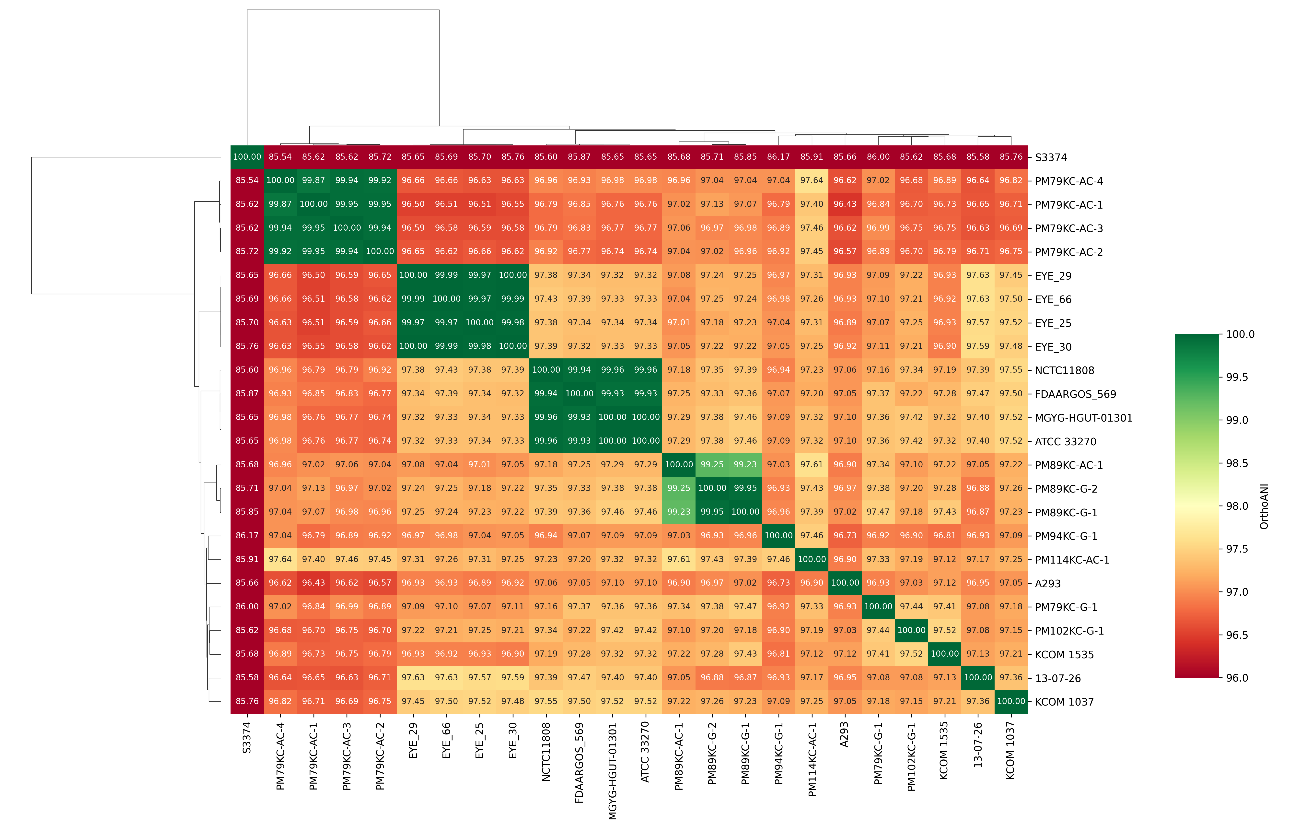


**Figure S2**. Heatmap showing Average Nucleotide Identity values as obtained by orthology (OrthoANI) between the *P. micra* genomes isolated in this study and others available at the NCBI database. *P. parva* (strain S3374) was used to root the comparison. The color scale (from green to red) was used to indicate the higher or lowest proximity between strains (green = closest species, red = farthest species).


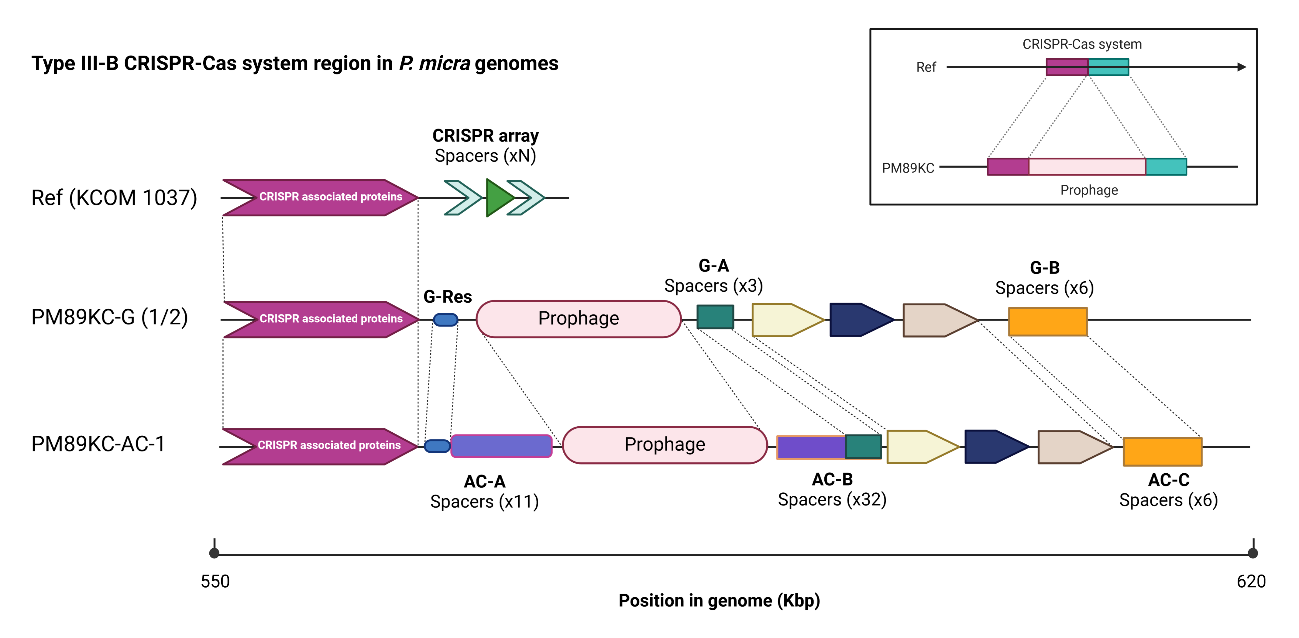


**Figure S3.** Graphic scheme of CRISPR-Cas sequences disposition in the genome of KCOM 1037 strain (used as reference) *vs* CRISPR-Cas system and prophage sequences arrangement in the *P. micra* PM89KC isolates genomes. Vertical dashed lines indicate physical co-localization of sequences between genomes (synteny). The image was created using BioRender (biorender.com).

**SUPPLEMENTARY TABLES**

**Table S1.** Differential abundance analysis (DAA) of *Parvimonas*, *Fusobacterium* and *Peptrostreptococcus* in stool samples between CRC and healthy subjects (98 CRC patients and 30 healthy controls) using ANCOM-BC at genus level, with a prevalence cut of 0.1 and adjusting the p-values by the Holm-Bonferroni method.

| **Genus** | **Log Fold Change** | **Differentially abundant** | | **Standard Error** | **Adjusted p-value** | **Significance** |
| --- | --- | --- | --- | --- | --- | --- |
| ***Parvimonas*** | 1.2498198 | | yes | 0.2255375 | 5.88 x 10^-6^ | *** |
| ***Fusobacterium*** | 1.2166415 | | yes | 0.2591823 | 5.22 x 10^-4^ | *** |
| ***Peptostreptococcus*** | 0.8944498 | | yes | 0.2027972 | 1.99 x 10^-3^ | ** |

*The more number of asterisks, the more statistically significant.

**Table S2**. Loci affected by non-synonymous mutations detected in the adenocarcinoma *P. micra* PM89KC-AC-1 strain, using the gingival PM89KC-G-1/2 strains as reference.

| **Locus tag** | **Gene** | **Product** | **Mutations** |
| --- | --- | --- | --- |
| NM219_00065 |  | DUF4026 domain-containing protein | 29 |
| NM219_00185 |  | Cna B-type domain-containing protein | 26 |
| NM219_01365 |  | Insulinase family protein | 21 |
| NM219_02545 |  | Lipoate--protein ligase | 21 |
| NM219_00335 |  | ATP-binding cassette domain-containing protein | 21 |
| NM219_07555 |  | GNAT family N-acetyltransferase | 20 |
| NM219_06555 | *ileS* | Isoleucine--tRNA ligase | 19 |
| NM219_06580 |  | Hypothetical protein | 19 |
| NM219_02520 |  | ABC transporter substrate-binding protein | 18 |
| NM219_06010 |  | CPBP family intramembrane metalloprotease | 18 |
| NM219_02540 | *lpdA* | Dihydrolipoyl dehydrogenase | 17 |
| NM219_06565 |  | Hypothetical protein | 17 |
| NM219_06705 |  | Hypothetical protein | 17 |
| NM219_06065 |  | ABC transporter ATP-binding protein/permease | 15 |
| NM219_06655 |  | MATE family efflux transporter | 15 |
| NM219_00660 |  | NADPH dehydrogenase | 15 |
| NM219_01685 |  | MFS transporter | 15 |
| NM219_01655 | *fmt* | Methionyl-tRNA formyltransferase | 14 |
| NM219_01280 |  | SufS family cysteine desulfurase | 14 |
| NM219_01860 |  | SdpI family protein | 13 |
| NM219_01245 |  | Type II secretion system F family protein | 12 |
| NM219_06570 |  | ABC transporter ATP-binding protein | 12 |
| NM219_06060 |  | ABC transporter ATP-binding protein/permease | 12 |
| NM219_01155 |  | TIGR01212 family radical SAM protein | 12 |
| NM219_01315 |  | ABC transporter ATP-binding protein/permease | 12 |
| NM219_00670 | *gcvPA* | Aminomethyl-transferring glycine dehydrogenase subunit GcvPA | 12 |
| NM219_06035 |  | ATP-binding cassette domain-containing protein | 12 |
| NM219_06005 |  | CPBP family intramembrane metalloprotease | 12 |
| NM219_01230 | *glmS* | Glutamine--fructose-6-phosphate transaminase (isomerizing) | 12 |
| NM219_01225 |  | ASCH domain-containing protein | 11 |
| NM219_00740 | *fabG* | 3-oxoacyl-ACP reductase FabG | 11 |
| NM219_00730 |  | Hypothetical protein | 11 |
| NM219_07775 |  | VWA domain-containing protein | 11 |
| NM219_06020 |  | CPBP family intramembrane metalloprotease | 11 |
| NM219_05990 |  | CPBP family intramembrane metalloprotease | 11 |
| NM219_00325 |  | Cna B-type domain-containing protein | 11 |
| NM219_00355 |  | Hypothetical protein | 11 |
| NM219_00525 |  | ABC transporter ATP-binding protein/permease | 11 |
| NM219_01215 |  | RluA family pseudouridine synthase | 10 |
| NM219_00295 |  | Dihydroorotase | 10 |
| NM219_00755 | *pepF* | Oligoendopeptidase F | 10 |
| NM219_07890 |  | Hypothetical protein | 10 |
| NM219_00165 |  | ABC transporter permease | 10 |
| NM219_01660 | *rsmB* | 16S rRNA (cytosine(967)-C(5))-methyltransferase RsmB | 10 |
| NM219_07795 |  | Sigma-70 family RNA polymerase sigma factor | 10 |
| NM219_02515 |  | Asparaginase | 10 |
| NM219_01945 |  | NUDIX hydrolase | 10 |
| NM219_06990 |  | Hypothetical protein | 10 |
| NM219_06000 |  | CPBP family intramembrane metalloprotease | 10 |
| NM219_01795 | *folK* | 2-amino-4-hydroxy-6- hydroxymethyldihydropteridine diphosphokinase | 10 |
| NM219_06660 | *topA* | Type I DNA topoisomerase | 10 |
| NM219_06670 |  | Flavin reductase family protein | 10 |
| NM219_01930 |  | TlyA family RNA methyltransferase | 10 |
| NM219_01150 |  | Peroxiredoxin | 9 |
| NM219_05955 |  | RNA-binding transcriptional accessory protein | 9 |
| NM219_01690 |  | TIGR01212 family radical SAM protein | 9 |
| NM219_07785 |  | NAD(P)H-dependent glycerol-3-phosphate dehydrogenase | 9 |
| NM219_02550 |  | GNAT family N-acetyltransferase | 9 |
| NM219_01680 |  | GNAT family N-acetyltransferase | 9 |
| NM219_07210 |  | Hypothetical protein | 9 |
| NM219_01940 | *recN* | DNA repair protein RecN | 9 |
| NM219_07805 | *rsmG* | 16S rRNA (guanine(527)-N(7))-methyltransferase RsmG | 8 |
| NM219_01730 |  | DNA alkylation repair protein | 8 |
| NM219_01165 | *sdaAA* | L-serine ammonia-lyase, iron-sulfur-dependent, subunit alpha | 8 |
| NM219_01160 | *sdaAB* | L-serine ammonia-lyase, iron-sulfur-dependent subunit beta | 8 |
| NM219_00540 |  | N-acetylmuramoyl-L-alanine amidase family protein | 8 |
| NM219_06720 |  | ABC transporter permease | 8 |
| NM219_05985 |  | CPBP family intramembrane metalloprotease | 8 |
| NM219_00835 |  | type I restriction-modification system subunit M | 8 |
| NM219_00825 |  | FtsX-like permease family protein | 8 |
| NM219_00175 | *brnQ* | branched-chain amino acid transport system II carrier protein | 8 |
| NM219_05980 |  | M20 family metallopeptidase | 8 |
| NM219_00720 |  | Undecaprenyl-diphosphate phosphatase | 8 |
| NM219_01890 |  | Pyridoxamine 5'-phosphate oxidase family protein | 7 |
| NM219_01790 | *folP* | Dihydropteroate synthase | 7 |
| NM219_01755 |  | Recombination regulator RecX | 7 |
| NM219_03200 |  | 1-acyl-sn-glycerol-3-phosphate acyltransferase | 7 |
| NM219_01880 |  | Histidine kinase | 7 |
| NM219_00300 | *pyrF* | Orotidine-5'-phosphate decarboxylase | 7 |
| NM219_06125 |  | Molybdopterin molybdotransferase MoeA | 7 |
| NM219_06025 |  | Peroxide stress protein YaaA | 7 |
| NM219_06030 |  | ABC transporter ATP-binding protein/permease | 7 |
| NM219_06070 |  | Proline--tRNA ligase | 7 |
| NM219_06550 |  | Arginine deiminase | 7 |
| NM219_06695 |  | ABC transporter ATP-binding protein/permease | 7 |
| NM219_00650 |  | Hypothetical protein | 7 |
| NM219_06610 | *ppdK* | Pyruvate, phosphate dikinase | 7 |
| NM219_00680 |  | ATP-NAD kinase family protein | 6 |
| NM219_07515 |  | C39 family peptidase | 6 |
| NM219_07175 | *dnaB* | Replicative DNA helicase | 6 |
| NM219_01820 |  | Glucosaminidase domain-containing protein | 6 |
| NM219_07895 |  | Isochorismatase family protein | 6 |
| NM219_05875 |  | RNA methyltransferase | 6 |
| NM219_06650 |  | Cysteine hydrolase | 6 |
| NM219_01295 |  | DNA-directed RNA polymerase subunit beta | 6 |
| NM219_06635 |  | Winged helix-turn-helix transcriptional regulator | 6 |
| NM219_06560 |  | Glutathione peroxidase | 6 |
| NM219_02555 | *typA* | Translational GTPase TypA | 6 |
| NM219_03190 |  | NAD(P)/FAD-dependent oxidoreductase | 6 |
| NM219_01125 |  | V-type ATP synthase subunit A | 6 |
| NM219_02505 |  | Glycine/betaine/sarcosine/D-proline family reductase Selenoprotein B | 5 |
| NM219_06980 | *thrS* | Threonine tRNA ligase | 5 |
| NM219_07535 |  | Aminotransferase class V-fold PLP-dependent enzyme | 5 |
| NM219_07800 | *mnmG* | tRNA uridine-5-carboxymethylaminomethyl(34) synthesis enzyme MnmG | 5 |
| NM219_01750 | *prfB* | Peptide chain release factor 2 | 5 |
| NM219_07505 |  | MBL fold metallo-hydrolase | 5 |
| NM219_07560 |  | Nuclear transport factor 2 family protein | 5 |
| NM219_01850 | *queA* | tRNA preQ1(34) S-adenosylmethionine ribosyltransferase-Isomerase QueA | 5 |
| NM219_06810 | *arcC* | Carbamate kinase | 5 |
| NM219_01955 |  | Segregation/condensation protein A | 5 |
| NM219_06725 |  | Iron-containing alcohol dehydrogenase | 5 |
| NM219_07605 |  | Hypothetical protein | 5 |
| NM219_07790 | *rpiB* | Ribose 5-phosphate isomerase B | 5 |
| NM219_06685 |  | Hypothetical protein | 5 |
| NM219_05995 |  | CPBP family intramembrane metalloprotease | 5 |
| NM219_00025 | *gyrB* | DNA topoisomerase (ATP-hydrolyzing) subunit B | 5 |
| NM219_00285 | *pyrB* | Aspartate carbamoyltransferase | 5 |
| NM219_00735 | *raiA* | Ribosome-associated translation inhibitor RaiA | 5 |
| NM219_00310 |  | Dihydroorotate dehydrogenase | 5 |
| NM219_01345 |  | ABC transporter ATP-binding protein/permease | 5 |
| NM219_00160 |  | Hypothetical protein | 5 |
| NM219_01240 |  | GspE/PulE family protein | 5 |
| NM219_01305 |  | Hypothetical protein | 5 |
| NM219_00550 |  | Double-cubane-cluster-containing anaerobic reductase | 5 |
| NM219_01480 |  | DNA/RNA non-specific endonuclease | 5 |
| NM219_00535 |  | ECF transporter S component | 5 |
| NM219_00190 |  | Hypothetical protein | 5 |
| NM219_00560 |  | Hypothetical protein | 5 |
| NM219_02565 |  | ATP-dependent RecD-like DNA helicase | 4 |
| NM219_07510 |  | Acyltransferase family protein | 4 |
| NM219_00750 | *brnQ* | Branched-chain amino acid transport system II carrier protein | 4 |
| NM219_00805 | *radA* | DNA repair protein RadA | 4 |
| NM219_01250 |  | Cysteine hydrolase | 4 |
| NM219_00545 |  | 2-hydroxyacyl-CoA dehydratase family protein | 4 |
| NM219_01275 |  | SufD family Fe-S cluster assembly protein | 4 |
| NM219_02525 |  | Thiamine pyrophosphate-dependent dehydrogenase E1 component subunit alpha | 4 |
| NM219_07550 | *pcp* | Pyroglutamyl-peptidase I | 4 |
| NM219_01895 |  | Dicarboxylate/amino acid:cation symporter | 4 |
| NM219_00710 |  | Hypothetical protein | 4 |
| NM219_01885 |  | Response regulator transcription factor | 4 |
| NM219_00305 |  | Dihydroorotate dehydrogenase electron transfer subunit | 4 |
| NM219_01300 | *rpoC* | DNA-directed RNA polymerase subunit beta' | 4 |
| NM219_06630 |  | Pseudouridine-5'-phosphate glycosidase | 4 |
| NM219_01765 |  | Hypothetical protein | 4 |
| NM219_05490 |  | TIGR01906 family membrane protein | 4 |
| NM219_01670 |  | Class I SAM-dependent methyltransferase | 4 |
| NM219_01735 |  | AraC family transcriptional regulator | 4 |
| NM219_07220 |  | Alpha/beta hydrolase | 4 |
| NM219_06040 |  | Cation diffusion facilitator family transporter | 4 |
| NM219_06665 |  | GNAT family N-acetyltransferase | 4 |
| NM219_07165 |  | DnaD domain protein | 4 |
| NM219_01805 | *deoC* | Deoxyribose-phosphate aldolase | 4 |
| NM219_00320 |  | Hypothetical protein | 4 |
| NM219_06085 |  | ATP-grasp domain-containing protein | 4 |
| NM219_00780 |  | Serpin family protein | 4 |
| NM219_00785 | *dnaX* | DNA polymerase III subunit gamma/tau | 4 |
| NM219_01175 | *rsxC* | Electron transport complex subunit RsxC | 3 |
| NM219_00795 |  | M20 family metallopeptidase | 3 |
| NM219_06190 |  | Thioester-forming surface-anchored protein | 3 |
| NM219_01235 |  | HAD family hydrolase | 3 |
| NM219_03210 |  | MATE family efflux transporter | 3 |
| NM219_01210 |  | TetR/AcrR family transcriptional regulator | 3 |
| NM219_07520 |  | DUF3343 domain-containing protein | 3 |
| NM219_05505 |  | LicD family protein | 3 |
| NM219_07500 | *rlmH* | 23S rRNA (pseudouridine(1915)-N(3))-methyltransferase RlmH | 3 |
| NM219_05945 |  | TDT family transporter | 3 |
| NM219_00800 |  | NADP-dependent malic enzyme | 3 |
| NM219_01455 | *cls* | Cardiolipin synthase | 3 |
| NM219_06675 | *thyA* | Thymidylate synthase | 3 |
| NM219_00625 |  | SAP domain-containing protein | 3 |
| NM219_00665 | *gcvPB* | Aminomethyl-transferring glycine dehydrogenase subunit GcvPB | 3 |
| NM219_07570 |  | SPASM domain-containing protein | 3 |
| NM219_01785 | *pepD* | Beta-Ala-His dipeptidase | 3 |
| NM219_00140 | *thiI* | tRNA 4-thiouridine(8) synthase ThiI | 3 |
| NM219_07725 |  | Cell surface protein | 3 |
| NM219_06625 |  | HAD family hydrolase | 3 |
| NM219_00570 |  | PIN/TRAM domain-containing protein | 3 |
| NM219_00580 |  | Ribonuclease H-like domain-containing protein | 3 |
| NM219_01925 |  | Polyprenyl synthetase family protein | 3 |
| NM219_01350 |  | D-alanine--D-alanine ligase | 3 |
| NM219_06590 |  | CPBP family intramembrane metalloprotease | 3 |
| NM219_07650 |  | M3 family oligoendopeptidase | 3 |
| NM219_07905 | *fni* | Type 2 isopentenyl-diphosphate Delta-isomerase | 3 |
| NM219_00725 |  | Hypothetical protein | 3 |
| NM219_00765 |  | AzlC family ABC transporter permease | 3 |
| NM219_06055 |  | YbaN family protein | 2 |
| NM219_00565 |  | Hypothetical protein | 2 |
| NM219_06080 |  | Amino acid racemase | 2 |
| NM219_06800 |  | DUF2974 domain-containing protein | 2 |
| NM219_07180 | *rplI* | 50S ribosomal protein L9 | 2 |
| NM219_06120 | *mobB* | Molybdopterin-guanine dinucleotide biosynthesis protein B | 2 |
| NM219_06700 |  | LacI family transcriptional regulator | 2 |
| NM219_06320 | *mobC* | Plasmid mobilization relaxosome protein MobC | 2 |
| NM219_07160 |  | ATP-binding protein | 2 |
| NM219_06150 |  | ECF transporter S component | 2 |
| NM219_06645 |  | Histidine triad nucleotide-binding protein | 2 |
| NM219_00690 |  | Hypothetical protein | 2 |
| NM219_06155 |  | Veg family protein | 2 |
| NM219_06605 |  | Kinase/pyrophosphorylase | 2 |
| NM219_06165 |  | Hypothetical protein | 2 |
| NM219_06595 |  | CPBP family intramembrane metalloprotease | 2 |
| NM219_06985 | *hisS* | Histidine--tRNA ligase | 2 |
| NM219_06185 |  | Pro-sigmaK processing inhibitor BofA family protein | 2 |
| NM219_06585 |  | GntR family transcriptional regulator | 2 |
| NM219_07910 |  | MFS transporter | 2 |
| NM219_07490 |  | Polysaccharide biosynthesis protein | 2 |
| NM219_02500 |  | Glycine/sarcosine/betaine reductase component B subunit | 2 |
| NM219_07780 |  | Hypothetical protein | 2 |
| NM219_03015 |  | Type III pantothenate kinase | 2 |
| NM219_01270 | *sufB* | Fe-S cluster assembly protein SufB | 2 |
| NM219_00030 | *gyrA* | DNA gyrase subunit A | 2 |
| NM219_07575 |  | MFS transporter | 2 |
| NM219_01825 |  | DMT family transporter | 2 |
| NM219_01835 | *ruvC* | Crossover junction endodeoxyribonuclease RuvC | 2 |
| NM219_07635 |  | DUF1700 domain-containing protein | 2 |
| NM219_00315 | *pyrE* | Orotate phosphoribosyltransferase | 2 |
| NM219_01960 | *scpB* | SMC-Scp complex subunit ScpB | 2 |
| NM219_01340 |  | Hypothetical protein | 2 |
| NM219_01920 | *xseB* | Exodeoxyribonuclease VII small subunit | 2 |
| NM219_01855 | *tgt* | tRNA guanosine(34) transglycosylase Tgt | 2 |
| NM219_00180 |  | Crp/Fnr family transcriptional regulator | 2 |
| NM219_01355 |  | UDP-N-acetylmuramoyl-tripeptide--D-alanyl-D- alanine Ligase | 2 |
| NM219_01865 |  | ABC transporter permease | 2 |
| NM219_07545 |  | DUF979 domain-containing protein | 2 |
| NM219_05445 | *murG* | Undecaprenyldiphospho-muramoylpentapeptide beta-N-Acetylglucosaminyltransferase | 2 |
| NM219_07440 |  | Carbohydrate-binding domain-containing protein | 2 |
| NM219_01145 |  | HAD family hydrolase | 2 |
| NM219_01725 |  | Nitroreductase family protein | 2 |
| NM219_01190 |  | Electron transport complex subunit E | 2 |
| NM219_07430 |  | PLP-dependent aminotransferase family protein | 2 |
| NM219_05965 |  | PucR family transcriptional regulator ligand-binding domain-containing protein | 2 |
| NM219_05960 | *lpdA* | Dihydrolipoyl dehydrogenase | 2 |
| NM219_01360 |  | M20/M25/M40 family metallo-hydrolase | 2 |
| NM219_05950 |  | DUF169 domain-containing protein | 2 |
| NM219_06755 |  | ABC transporter permease | 2 |
| NM219_05915 |  | RNA methyltransferase | 2 |
| NM219_07875 |  | GIY-YIG nuclease family protein | 2 |
| NM219_00150 |  | Aspartate carbamoyltransferase | 1 |
| NM219_00170 |  | ATP-binding cassette domain-containing protein | 1 |
| NM219_06805 |  | DMT family transporter | 1 |
| NM219_06815 | *argF* | Ornithine carbamoyltransferase | 1 |
| NM219_00145 |  | Hypothetical protein | 1 |
| NM219_06835 |  | tRNA-Phe | 1 |
| NM219_07880 |  | MmcQ/YjbR family DNA-binding protein | 1 |
| NM219_00040 |  | Hypothetical protein | 1 |
| NM219_06780 | *rpsG* | 30S ribosomal protein S7 | 1 |
| NM219_00045 |  | SigB/SigF/SigG family RNA polymerase sigma factor | 1 |
| NM219_07185 |  | DHH family phosphoesterase | 1 |
| NM219_07715 |  | SHIRT domain-containing protein | 1 |
| NM219_06905 |  | tRNA-Glu | 1 |
| NM219_00330 | *purD* | Phosphoribosylamine--glycine ligase | 1 |
| NM219_07275 |  | Ig-like domain-containing protein | 1 |
| NM219_07350 |  | Hypothetical protein | 1 |
| NM219_00530 | *yjeM* | Glutamate/gamma-aminobutyrate family transporter YjeM | 1 |
| NM219_07170 |  | GNAT family N-acetyltransferase | 1 |
| NM219_07495 |  | tRNA 2-thiocytidine(32) synthetase TtcA | 1 |
| NM219_07530 | *yedE* | YedE family putative selenium transporter | 1 |
| NM219_06975 |  | CPBP family intramembrane metalloprotease | 1 |
| NM219_06965 |  | Dicarboxylate/amino acid:cation symporter | 1 |
| NM219_06955 |  | Cupin domain-containing protein | 1 |
| NM219_07600 | *glpK* | Glycerol kinase GlpK | 1 |
| NM219_06940 |  | YjjI family glycine radical enzyme | 1 |
| NM219_07190 |  | YybS family protein | 1 |
| NM219_07610 |  | ATP-binding cassette domain-containing protein | 1 |
| NM219_06920 | *tsaB* | tRNA (adenosine(37)-N6)-threonylcarbamoyltransferase complex dimerization subunit type 1 TsaB | 1 |
| NM219_06760 | *gpmA* | 2,3-diphosphoglycerate-dependent phosphoglycerate mutase | 1 |
| NM219_01715 |  | mechanosensitive ion channel family protein | 1 |
| NM219_06750 |  | ABC transporter permease | 1 |
| NM219_05455 | *murD* | UDP-N-acetylmuramoyl-L-alanine--D-glutamate ligase | 1 |
| NM219_05360 |  | LXG domain-containing protein | 1 |
| NM219_05265 |  | V-type ATP synthase subunit E | 1 |
| NM219_04995 |  | Peptidylprolyl isomerase | 1 |
| NM219_03990 |  | VanW family protein | 1 |
| NM219_03975 |  | Tetratricopeptide repeat protein | 1 |
| NM219_03205 |  | Bifunctional 4-hydroxy-3-methylbut-2-enyl diphosphate reductase/30S ribosomal protein S1 | 1 |
| NM219_03010 |  | Hypothetical protein | 1 |
| NM219_01255 |  | DUF1576 domain-containing protein | 1 |
| NM219_02530 |  | Alpha-ketoacid dehydrogenase subunit beta | 1 |
| NM219_01285 |  | SUF system NifU family Fe-S cluster assembly protein | 1 |
| NM219_02510 |  | Sodium-dependent transporter | 1 |
| NM219_02485 |  | GntR family transcriptional regulator | 1 |
| NM219_02480 |  | Hypothetical protein | 1 |
| NM219_01965 |  | rRNA pseudouridine synthase | 1 |
| NM219_01950 |  | Site-2 protease family protein | 1 |
| NM219_01915 | *xseA* | Exodeoxyribonuclease VII large subunit | 1 |
| NM219_01870 |  | ABC transporter permease | 1 |
| NM219_01445 |  | Epoxyqueuosine reductase QueH | 1 |
| NM219_01840 | *ruvA* | Holliday junction branch migration protein RuvA | 1 |
| NM219_01460 | *truA* | tRNA pseudouridine(38-40) synthase TruA | 1 |
| NM219_01810 | *msrB* | Peptide-methionine (R)-S-oxide reductase MsrB | 1 |
| NM219_01800 | *deoD* | Purine-nucleoside phosphorylase | 1 |
| NM219_01475 |  | Hypothetical protein | 1 |
| NM219_01780 | *pepT* | Peptidase T | 1 |
| NM219_01775 |  | DnaJ domain-containing protein | 1 |
| NM219_01760 |  | GIY-YIG nuclease family protein | 1 |
| NM219_01490 |  | Hypothetical protein | 1 |
| NM219_01665 |  | VOC family protein | 1 |
| NM219_01740 |  | Hypothetical protein | 1 |
| NM219_05440 |  | FtsQ-type POTRA domain-containing protein | 1 |
| NM219_05495 |  | YebC/PmpR family DNA-binding transcriptional regulator | 1 |
| NM219_06735 |  | M18 family aminopeptidase | 1 |
| NM219_05865 |  | Hypothetical protein | 1 |
| NM219_00555 |  | DUF3343 domain-containing protein | 1 |
| NM219_06710 |  | ABC transporter ATP-binding protein | 1 |
| NM219_06690 |  | ABC transporter ATP-binding protein/permease | 1 |
| NM219_00575 |  | CarD family transcriptional regulator | 1 |
| NM219_06680 |  | Type B 50S ribosomal protein L31 | 1 |
| NM219_00675 | *gcvT* | Glycine cleavage system aminomethyltransferase GcvT | 1 |
| NM219_06600 |  | L,D-transpeptidase/peptidoglycan binding protein | 1 |
| NM219_00715 |  | QueT transporter family protein | 1 |
| NM219_00760 |  | AzlD domain-containing protein | 1 |
| NM219_06505 |  | Type IV secretory system conjugative DNA transfer family protein | 1 |
| NM219_06430 |  | Hypothetical protein | 1 |
| NM219_06415 |  | ABC transporter ATP-binding protein/permease | 1 |
| NM219_06375 |  | Alpha/beta hydrolase | 1 |
| NM219_06180 | *tmk* | dTMP kinase | 1 |
| NM219_01720 |  | DUF951 domain-containing protein | 1 |
| NM219_06145 |  | Class I SAM-dependent RNA methyltransferase | 1 |
| NM219_06140 |  | Serine/threonine-protein phosphatase | 1 |
| NM219_00775 |  | YbaB/EbfC family nucleoid-associated protein | 1 |
| NM219_06105 |  | tRNA-Gly | 1 |
| NM219_06090 | *asnB* | Asparagine synthase (glutamine-hydrolyzing) | 1 |
| NM219_00840 |  | Restriction endonuclease subunit S | 1 |
| NM219_01030 |  | ACP S-malonyltransferase | 1 |
| NM219_01130 |  | V-type ATP synthase subunit B | 1 |
| NM219_01135 |  | V-type ATP synthase subunit D | 1 |
| NM219_01180 |  | RnfABCDGE type electron transport complex subunit D | 1 |
| NM219_01185 |  | RnfABCDGE type electron transport complex subunit G | 1 |
| NM219_05970 |  | DUF5058 family protein | 1 |
| NM219_01195 | *rsxA* | Electron transport complex subunit RsxA | 1 |
| NM219_05925 |  | TrkH family potassium uptake protein | 1 |
| NM219_06160 |  | LPXTG cell wall anchor domain-containing protein | 1 |

**Table S3.** Comparison of genes identified in the cross-shaped structure found in the *P. micra* PM89KC-AC-1 isolate, composed of two prophages.

| **Left side of cross (position ~600kb)** | | **Right side of cross (position ~800kb)** | | **Identity (%)** | **Alignment length** | **Mismatch** | **Evalue** | **Bitscore** |
| --- | --- | --- | --- | --- | --- | --- | --- | --- |
| **Locus tag** | **Product** | **Locus tag** | **Product** |  |  |  |  |  |
| NM221_02680 | CD1845 family protein | NM221_03570 | CD1845 family protein | 73.4 | 94 | 24 | 3.76 e-38 | 115 |
| NM221_02685 | Replication initiator protein A | NM221_03575 | Replication initiator protein A | 60.8 | 260 | 95 | 6.17 e-98 | 280 |
| NM221_02690 | ATP-binding protein | NM221_03580 | ATP-binding protein | 83.4 | 283 | 47 | 6.26 e-179 | 487 |
| NM221_02695 | PcfB family protein | NM221_03585 | PcfB family protein | 83.9 | 161 | 26 | 9.81 e-84 | 236 |
| NM221_02700 | BRO family protein | - |  |  |  |  |  |  |
| NM221_02705 | Type IV secretory system conjugative DNA transfer family protein | NM221_03590 | Type IV secretory system conjugative DNA transfer family protein | 94.9 | 593 | 30 | 0 | 1117 |
| NM221_02710 | Hypothetical protein | - |  |  |  |  |  |  |
| - |  | NM221_03595 | DUF6037 family protein |  |  |  |  |  |
| - |  | NM221_03600 | Nucleotide-binding protein |  |  |  |  |  |
| NM221_02715 | Single-stranded DNA-binding protein | NM221_03605 | Single-stranded DNA-binding protein | 89.3 | 103 | 11 | 2.65 e-65 | 185 |
| NM221_02720 | Maff2 family protein | NM221_03610 | Maff2 family protein | 93 | 71 | 5 | 4.2 e-43 | 126 |
| NM221_02725 | CD0415/CD1112 family protein | NM221_03615 | CD0415/CD1112 family protein | 89.9 | 287 | 29 | 2.54 e-180 | 491 |
| NM221_02730 | Hypothetical protein | NM221_03620 | Conjugal transfer protein | 30.2 | 86 | 60 | 2.8 e-11 | 47.4 |
| NM221_02735 | PrgI family protein | NM221_03625 | PrgI family protein | 81.1 | 127 | 24 | 2.32 e-69 | 197 |
| NM221_02740 | ATP-binding protein | NM221_03630 | ATP-binding protein | 89.7 | 816 | 77 | 0 | 1427 |
| NM221_02745 | NlpC/P60 family protein | NM221_03635 | CHAP domain-containing protein | 50.1 | 729 | 287 | 4.3 e-206 | 588 |
| NM221_02750 | Conjugal transfer protein | NM221_03640 | Conjugal transfer protein | 83.3 | 78 | 13 | 1.6 e-39 | 118 |
| NM221_02755 | DUF4366 domain-containing protein | NM221_03645 | DUF4366 domain-containing protein | 67.3 | 165 | 40 | 8.07 e-57 | 182 |
| NM221_02760 | DNA topoisomerase 3 | NM221_03650 | DNA topoisomerase 3 | 86.5 | 563 | 76 | 0 | 965 |
| - |  | NM221_03655 | AbrB/MazE/SpoVT family DNA-binding domain-containing protein |  |  |  |  |  |
| - |  | NM221_03660 | Helix-turn-helix domain-containing protein |  |  |  |  |  |
| NM221_02765 | DEAD/DEAH box helicase family protein | NM221_03665 | DEAD/DEAH box helicase family protein | 83.1 | 3044 | 376 | 0 | 4836 |
| NM221_02770 | Single-stranded DNA-binding protein | - |  |  |  |  |  |  |
| - |  | NM221_03670 | DNA-binding protein |  |  |  |  |  |
| - |  | NM221_03675 | Helix-turn-helix transcriptional regulator |  |  |  |  |  |
| - |  | NM221_03680 | Hypothetical protein |  |  |  |  |  |
| NM221_02775 | Relaxase/mobilization nuclease domain-containingprotein | NM221_03685 | Relaxase/mobilization nuclease domain-containingprotein | 88.7 | 443 | 50 | 1.8 e-286 | 772 |
| NM221_02780 | MobC family plasmid mobilization relaxosome protein | NM221_03690 | MobC family plasmid mobilization relaxosome protein | 93.1 | 116 | 8 | 2.49 e-76 | 214 |
| - |  | - |  |  |  |  |  |  |
| - |  | NM221_03700 | Hypothetical protein |  |  |  |  |  |
| - |  | NM221_03705 | Hypothetical protein |  |  |  |  |  |
| NM221_02785 | Subtilosin A family bacteriocin | - |  |  |  |  |  |  |
| NM221_02790 | Radical SAM protein | - |  |  |  |  |  |  |
| NM221_02795 | Hypothetical protein | - |  |  |  |  |  |  |
| NM221_02800 | ABC transporter ATP-binding protein | NM221_03710 | ABC transporter ATP-binding protein | 21.1 | 228 | 140 | 2.09 e-07 | 42 |
| NM221_02805 | Hypothetical protein | - |  |  |  |  |  |  |
| NM221_02810 | Helix-turn-helix transcriptional regulator | NM221_03695 (MOVED) | Helix-turn-helix transcriptional regulator | 34.5 | 58 | 38 |  |  |
| NM221_02815 | Subtilosin A family bacteriocin | - |  |  |  |  |  |  |
| NM221_02820 | Radical SAM protein | - |  |  |  |  |  |  |
| NM221_02825 | Hypothetical protein | - |  |  |  |  |  |  |
| NM221_02830 | Hypothetical protein | - |  |  |  |  |  |  |
| NM221_02835 | Hypothetical protein | - |  |  |  |  |  |  |
| NM221_02840 | Hypothetical protein | - |  |  |  |  |  |  |
| NM221_02845 | Sigma 70 family RNA polymerase sigma factor | NM221_03715 | Sigma-70 family RNA polymerase sigma factor | 97.1 | 136 | 4 | 2.5 e-90 | 251 |
| NM221_02850 | Hypothetical protein | - |  |  |  |  |  |  |
| NM221_02855 | Recombinase family protein | - |  |  |  |  |  |  |
| NM221_02860 | Recombinase family protein | - |  |  |  |  |  |  |
| NM221_02865 | Recombinase family protein | NM221_03720 | Recombinase family protein | 27.1 | 343 | 216 | 8.24 e-24 | 96.7 |
| NM221_02870 | ORF6N domain-containing protein | - |  |  |  |  |  |  |

**Table S4.** CRISPR-Cas systems found on *P. micra* analyzed genomes, with their CRISPR arrays, spacer counts and consensus repeats. The consensus repeats have been switched to match orientation in all genomes. In small CRISPR arrays (i.e. PM89KC-G-1 A). For isolates with very high identity (EYE group or PM89KC-AC isolates 1-4) only one of the isolates was analyzed.

| ***P. micra* strain** | **CRISPR-Cas system type** | **CRISPR arrays** | **Spacer count** | **Orientation** | **Consensus repeat** |
| --- | --- | --- | --- | --- | --- |
| PM89KC-G-1 | CAS-III-B | A | 3 | 3 | GTTTAAATAGAAACATACTGTAATGTAAAT |
|  |  | B | 6 | 6 | GTTTAAATAGAAACATACTGTAATGTAAAT |
| PM89KC-Ac-1 | CAS-III-B | A | 11 | 11 | GTTTAAATAGAAACATACTGTAATGTAAAT |
|  |  | B | 32 | 32 | GTTTAAATAGAAACATACTGTAATGTAAAT |
|  |  | C | 6 | 6 | GTTTAAATAGAAACATACTGTAATGTAAAT |
| KCOM 1037 | CAS-III-B | A | 12 | 12 | GTTTAAATAGAAACATACTGTAATGTAAAT |
|  |  | B | 10 | 10 | GTTTAAATAGAAACATACTGTAATGTAAAT |
| NCTC11808 | CAS-III-A | A | 18 | 18 | GTTTAAATAGAAACATACTGTAATGTAAAT |
|  |  | B | 11 | 11 | GTTTAAATAGAAACATACTGTAATGTAAAT |
| PM114KC-Ac-1 | CAS-III-A | A | 25 | 25 | GTTTAAATAGAAACATACTGTAATGTAAAT |
| ATCC 33270 | CAS-III-A | A | 21 | 21 | GTTTAAATAGAAACATACTGTAATGTAAAT |
| FDAARGOS 569 | CAS-III-A | A | 21 | 21 | GTTTAAATAGAAACATACTGTAATGTAAAT |
|  |  | B | 11 | 11 | GTTTAAATAGAAACATACTGTAATGTAAAT |
| MGYG-HGUT-01301 | CAS-III-A | A | 21 | 21 | GTTTAAATAGAAACATACTGTAATGTAAAT |
|  |  | B | 11 | 11 | GTTTAAATAGAAACATACTGTAATGTAAAT |
| KCOM 1535 | CAS-II-A | **-** | 0 | 0 | - |
|  | CAS-III-A | A | 3 | 3 | GTTTAAATAGAAACATACTGTAATGTAAAT |
|  |  | B | 19 | 19 | GTTTAAATAGAAACATACTGTAATGTAAAT |
| EYE_30 | CAS-II-C | A | 14 | 14 | GTTTGAGAGTAATGTAATCTACATAGGTACTAAGAC |
|  |  | B | 12 | 12 | GTTTGAGAGTAATGTAATCTACATAGGTACTAAGAC |
|  | CAS-III-B | C | 14 | 14 | GTTTAAATAGAAACATACTGTAATGTAAAT |
|  |  | D | 41 | 41 | GTTTAAATAGAAACATACTGTAATGTAAAT |
| PM79KC-G-1 | CAS-II-A | A | 15 | 15 | GTTTGAGAGTAATGTAATCTACATAGGTACTAAGAC |
|  |  | B | 3 | 3 | GTTTGAGAGTAATGTAATCTACATAGGTACTAAGAC |
|  | CAS-III-B | C | 13 | 13 | GTTTAAATAGAAACATACTGTAATGTAAAT |
|  |  | D | 34 | 34 | GTTTAAATAGAAACATACTGTAATGTAAAT |
| PM102KC-G-1 | CAS-II-A | A | 9 | 9 | GTTTGAGAGTAATGTAATCTACATAGGTACTAAGAC |
|  | CAS-III-A | **-** | 0 | 0 | **-** |
| PM79KC-Ac-2 | CAS-I-E | A | 3 | 3 | GTACTCCCCGCGCAAGCAGGGGTGATCC |
|  |  | B | 16 | 16 | GTACTCCCCGCGCAAGCAGGGGTGATCC |
| A293 | CAS-I-B | A | 10 | 10 | GTTTAAATAGAAACATACTGTAATGTAAAT |
|  | CAS-III-B |  |  |  |  |
| PM94KC-G-1 | CAS-I-B | A | 9 | 9 | GTTTAAATAGAAACATACTGTAATGTAAAT |
|  | CAS-III-B | B | 31 | 31 | GTTTAAATAGAGACATACTGTAATGTAAAT |
| 13/07/2026 | CAS-I-B | - | 0 | 0 | - |
|  | CAS-III-B |  |  |  | - |

**Table S5.** Virulence factors present in *P. micra* strain PM89KC-AC-1, using DIAMOND against the Virulence Factor Database (version 2021-10-04).

| **Query locus tag** | **Query description** | **Gene** | **Subject description** | **Percentage of identical matches** | **Alignment length/Gene length** | **Mismatch** | **Number of gap openings** | **E-value** | **Bit score** |
| --- | --- | --- | --- | --- | --- | --- | --- | --- | --- |
| NM221_02925 | Chaperonin GroEL | *groEL* | Chaperonin GroEL | 73.8 | 542/542 | 139 | 2 | 5.31 e-255 | 743.0 |
| NM221_06660 | Elongation factor Tu | *tufA* | Elongation factor Tu | 70.7 | 396/394 | 113 | 3 | 2.15 e-193 | 578.0 |
| NM221_04890 | ATP-dependent Clp endopeptidase proteolytic subunit ClpP | *clpP* | ATP-dependent Clp endopeptidase proteolytic subunit ClpP | 64.6 | 192/198 | 68 | 0 | 4.66 e-72 | 254.0 |
| NM221_05640 | UDP-glucose 4-epimerase GalE | *galE* | UDP-glucose 4-epimerase GalE | 58.9 | 336/338 | 137 | 1 | 6.67 e-134 | 420.0 |
| NM221_01810 | Peptide-methionine (R)-S-oxide reductase MsrB | *msrA/BpilB* | Trifunctional thioredoxin/methionine sulfoxide reductase A/B protein | 57.6 | 330/522 | 126 | 4 | 3.97 e-115 | 382.0 |
| NM221_03250 | Isoprenyl transferase | *cpsA* | Undecaprenyl diphosphate synthase | 48.7 | 234/271 | 116 | 1 | 1.47 e-60 | 229.0 |
| NM221_05585 | WXG100 family type VII secretion target | *esxA* | Type VII secretion system secreted protein EsxA | 47.3 | 93/97 | 49 | 0 | 3.85 e-11 | 92.8 |
| NM221_00270 | Aspartate carbamoyltransferase | *pyrB* | Aspartate carbamoyltransferase | 47.2 | 303/306 | 153 | 5 | 1.37 e-66 | 249.0 |
| NM221_03445 | AAA family ATPase | *clpC* | Endopeptidase Clp ATP-binding chain C | 46.9 | 778/820 | 340 | 6 | 2.98 e-212 | 660.0 |
| NM221_02370 | Superoxide dismutase | *sodB* | Superoxide dismutase | 46.9 | 194/196 | 96 | 1 | 1.50 e-46 | 190.0 |
| NM221_01030 | 3-hydroxyacyl-ACP dehydratase FabZ | *fabZ* | (3R)-hydroxymyristoyl ACP dehydratase | 46.1 | 128/173 | 67 | 2 | 4.04 e-31 | 107.0 |
| NM221_05560 | Type VII secretion protein EssC | *essC* | Type VII secretion system protein EssC, FtsK/SpoIIIE family ATPase | 46.0 | 1324/1479 | 699 | 9 | 0.0 | 1169.0 |
| NM221_01060 | RdgB/HAM1 family non-canonical purine NTP pyrophosphatase | *orfM* | Deoxyribonucleotide triphosphate pyrophosphatase | 45.6 | 193/195 | 100 | 3 | 1.18 e-42 | 139.0 |
| NM221_01020 | 3-oxoacyl-[acyl-carrier-protein] reductase | *cylG* | 3-ketoacyl-ACP-reductase CylG | 45.5 | 244/240 | 123 | 4 | 4.30 e-50 | 199.0 |
| NM221_04545 | ATP-dependent Clp protease ATP-binding subunit | *clpC* | Endopeptidase Clp ATP-binding chain C | 45.3 | 811/820 | 426 | 6 | 5.53 e-209 | 647.0 |
| NM221_05490 | YebC/PmpR family DNA-binding transcriptional regulator | *CBU_1566* | Coxiella Dot/Icm type IVB secretion system translocated effector | 44.5 | 245/244 | 133 | 3 | 3.34 e-50 | 202.0 |
| NM221_00265 | Polysaccharide biosynthesis protein | *cap8D* | Capsular polysaccharide synthesis enzyme Cap8D | 44.1 | 508/607 | 277 | 7 | 5.21 e-121 | 407.0 |
| NM221_02220 | Ribulose-phosphate 3-epimerase | *rpe* | Ribulose-phosphate 3-epimerase | 43.0 | 200/222 | 113 | 1 | 2.60 e-42 | 181.0 |
| NM221_03160 | ABC transporter ATP-binding protein | *hitC* | Iron(III) ABC transporter, ATP-binding protein | 42.6 | 242/351 | 127 | 4 | 3.14 e-35 | 170.0 |
| NM221_03395 | Sugar transferase | *bplG* | Probable sugar transferase | 42.6 | 197/197 | 108 | 2 | 5.82 e-42 | 139.0 |
| NM221_00260 | UTP-glucose-1-phosphate uridylyltransferase | *hasC* | UTP-glucose-1-phosphate uridylyltransferase | 42.5 | 294/295 | 155 | 6 | 1.79 e-58 | 227.0 |
| NM221_00945 | ABC transporter ATP-binding protein | *hitC* | Iron(III) ABC transporter, ATP-binding protein | 42.4 | 236/351 | 136 | 0 | 7.95 e-47 | 199.0 |
| NM221_01235 | GspE/PulE family protein | *exeE* | General secretory pathway protein E | 40.8 | 375/501 | 219 | 3 | 9.89 e-99 | 300.0 |
| NM221_04175 | Bifunctional (p)ppGpp synthetase/guanosine-3',5'-bis(diphosphate) 3'-pyrophosphohydrolase | *relA* | Probable GTP pyrophosphokinase RelA (ATP:GTP 3'-pyrophosphotransferase) (PPGPP synthetase I) ((P)PPGPP synthetase) (GTP diphosphokinase) | 40.4 | 727/790 | 418 | 6 | 5.61 e-179 | 566.0 |
| NM221_04985 | Peptidylprolyl isomerase | *lirB* | Dot/Icm type IV secretion system effector LirB | 40.4 | 193/188 | 65 | 5 | 6.41 e-16 | 108.0 |
| NM221_04330 | Phosphoribosylformylglycinamidine cyclo-ligase | *purM* | Phosphoribosylaminoimidazole synthetase | 39.7 | 345/347 | 200 | 5 | 7.75 e-70 | 257.0 |
| NM221_00725 | 3-oxoacyl-ACP reductase FabG | *cylG* | 3-ketoacyl-ACP-reductase CylG | 39.5 | 238/240 | 136 | 4 | 4.49 e-54 | 171.0 |
| NM221_00915 | ABC transporter ATP-binding protein | *mntA* | ABC transporter ATP-binding protein MntA | 38.9 | 226/251 | 134 | 1 | 3.27 e-51 | 164.0 |
| NM221_01535 | Glycosyltransferase family 2 protein | *FTT_0797* | Glycosyltransferase family 2 protein | 38.9 | 216/319 | 119 | 2 | 1.52 e-45 | 154.0 |
| NM221_00595 | Lipoate--protein ligase | *lplA1* | Lipoate protein ligase | 38.3 | 329/331 | 195 | 5 | 1.47 e-55 | 222.0 |
| NM221_04280 | Helix-turn-helix domain-containing protein | *cylR2* | Cytolysin regulator R2 | 37.9 | 58/66 | 36 | 0 | 8.00 e-08 | 42.0 |
| NM221_00170 | Cna B-type domain-containing protein | *cna* | Collagen adhesin precursor | 37.7 | 793/1183 | 394 | 7 | 9.48 e-158 | 550.0 |
| NM221_06300 | ABC transporter ATP-binding protein/permease | *msbA* | Lipid transporter ATP-binding/permease | 37.2 | 406/587 | 228 | 10 | 4.70 e-55 | 233.0 |
| NM221_05005 | Signal peptidase II | *lspA* | Signal peptidase II | 37.2 | 148/154 | 91 | 2 | 2.53 e-08 | 89.4 |
| NM221_04085 | Pantetheine-phosphate adenylyltransferase | *kdtB* | Lipopolysaccharide core biosynthesis protein | 37.1 | 151/157 | 94 | 1 | 4.10 e-13 | 102.0 |
| NM221_07345 | Response regulator transcription factor | *phoP* | Possible two component system response transcriptional positive regulator PhoP | 36.9 | 225/247 | 133 | 4 | 5.78 e-27 | 140.0 |
| NM221_01565 | Energy-coupling factor transporter ATPase | *cylA* | ABC (ATP-binding cassette) transporter CylA | 36.7 | 210/309 | 120 | 6 | 1.76 e-13 | 110.0 |
| NM221_02810 | Helix-turn-helix transcriptional regulator | *cylR2* | Cytolysin regulator R2 | 36.7 | 60/66 | 38 | 0 | 8.15 e+05 | 46.6 |
| NM221_04370 | Response regulator transcription factor | *bfmR* | Biofilm-controlling response regulator | 36.6 | 224/238 | 139 | 2 | 2.68 e-44 | 145.0 |
| NM221_01380 | ABC transporter ATP-binding protein | *hitC* | Iron(III) ABC transporter, ATP-binding protein | 36.6 | 216/351 | 120 | 5 | 7.87 e-41 | 140.0 |
| NM221_03935 | Response regulator transcription factor | *bfmR* | Biofilm-controlling response regulator | 36.5 | 181/238 | 112 | 2 | 8.53 e-28 | 103.0 |
| NM221_01575 | NFACT family protein | *fbpA* | Fibronectin-binding protein FbpA | 36.2 | 578/591 | 354 | 10 | 6.02 e-92 | 330.0 |
| NM221_02130 | ABC transporter ATP-binding protein | *hitC* | Iron(III) ABC transporter. ATP-binding protein | 36.2 | 221/351 | 129 | 4 | 8.75 e-33 | 119.0 |
| NM221_00975 | Acyl carrier protein | *acpXL* | Acyl carrier protein | 36.2 | 69/78 | 44 | 0 | 1.13 e-09 | 47.0 |
| NM221_03845 | Phosphopantetheine-binding protein | *acpXL* | Acyl carrier protein | 36.2 | 47/78 | 30 | 0 | 1.73 e-05 | 36.2 |
| NM221_02255 | D-alanine-poly(phosphoribitol) ligase subunit DltC | *clbE* | Colibactin biosynthesis aminomalonyl-acyl carrier protein ClbE | 36.0 | 50/82 | 32 | 0 | 5.57 e+11 | 35.0 |
| NM221_02315 | ABC transporter ATP-binding protein | *hitC* | Iron(III) ABC transporter, ATP-binding protein | 35.9 | 309/351 | 190 | 3 | 6.61 e-58 | 188.0 |
| NM221_02610 | Phospho-sugar mutase | *yhxB/manB* | Phosphomannomutase | 35.7 | 554/550 | 309 | 13 | 7.97 e-75 | 284.0 |
| NM221_02550 | Lipoate--protein ligase family protein | *lplA1* | Lipoate protein ligase | 35.7 | 291/331 | 177 | 5 | 1.01 e-52 | 173.0 |
| NM221_00910 | Zinc ABC transporter substrate-binding protein | *lpeA* | Lipoprotein promoting cell invasion | 35.5 | 282/310 | 167 | 6 | 3.46 e-48 | 161.0 |
| NM221_05300 | ABC transporter ATP-binding protein/permease | *msbA* | Lipid transporter ATP-binding/permease | 35.2 | 520/587 | 311 | 6 | 2.72 e-88 | 283.0 |
| NM221_06705 | Ornithine carbamoyltransferase | *pyrB* | Aspartate carbamoyltransferase | 34.9 | 186/306 | 118 | 3 | 1.01 e-03 | 85.1 |
| NM221_03105 | Bifunctional UDP-N-acetylglucosamine diphosphorylase/glucosamine-1-phosphate N-acetyltransferase GlmU | *glmU* | UDP-N-acetylglucosamine pyrophosphorylase/glucosamine-1-phosphate N-acetyltransferase | 34.8 | 454/455 | 276 | 9 | 1.08 e-73 | 236.0 |
| NM221_00110 | S8 family serine peptidase | *scpB* | Streptococcal C5a peptidase | 34.5 | 946/1150 | 515 | 21 | 7.60 e-126 | 462.0 |
| NM221_00310 | Cna B-type domain-containing protein | *cna* | Collagen adhesin precursor | 34.5 | 799/1183 | 429 | 35 | 8.85 e-82 | 328.0 |
| NM221_04415 | ABC transporter ATP-binding protein | *hitC* | Iron(III) ABC transporter, ATP-binding protein | 34.5 | 220/351 | 131 | 8 | 2.08 e-29 | 109.0 |
| NM221_01485 | ABC transporter ATP-binding protein | *cesC* | ABC transporter ATP-binding protein, CesC | 34.2 | 222/291 | 140 | 3 | 1.38 e-26 | 143.0 |
| NM221_01875 | ABC transporter ATP-binding protein | *cylA* | ABC (ATP-binding cassette) transporter CylA | 34.2 | 225/309 | 140 | 5 | 5.90 e-15 | 115.0 |
| NM221_03355 | Peptidylprolyl isomerase | *PEB4* | Major antigenic peptide PEB-cell binding factor | 34.0 | 253/273 | 152 | 8 | 5.26 e-28 | 105.0 |
| NM221_05735 | Iron ABC transporter permease | *shuU* | Permease of iron compound ABC transport system | 33.9 | 283/318 | 178 | 4 | 7.94 e-35 | 166.0 |
| NM221_04145 | Ribosome maturation factor RimP | *CBU_1434* | Coxiella Dot/Icm type IVB secretion system translocated effector | 33.9 | 124/153 | 81 | 1 | 3.91 e-17 | 70.9 |
| NM221_07775 | Response regulator transcription factor | *bfmR* | Biofilm-controlling response regulator | 33.8 | 222/238 | 142 | 4 | 2.53 e-34 | 120.0 |
| NM221_00330 | Glycine C-acetyltransferase | *wcbT* | Acyl-CoA transferase | 33.7 | 380/439 | 248 | 3 | 8.60 e-54 | 221.0 |
| NM221_00255 | DegT/DnrJ/EryC1/StrS family aminotransferase | *bplF* | Lipopolysaccharide biosynthesis protein | 33.6 | 411/410 | 234 | 9 | 1.41 e-54 | 224.0 |
| NM221_07820 | LCP family protein | *bpsX* | LytR family transcriptional regulator | 33.5 | 188/304 | 116 | 3 | 3.78 e-30 | 114.0 |
| NM221_07210 | ATP-binding cassette domain-containing protein | *cesC* | ABC transporter ATP-binding protein, CesC | 33.3 | 228/291 | 148 | 3 | 5.04 e-18 | 121.0 |
| NM221_07180 | Response regulator transcription factor | *bfmR* | Biofilm-controlling response regulator | 33.3 | 222/238 | 137 | 3 | 8.29 e-14 | 105.0 |
| NM221_02585 | Phosphonate ABC transporter ATP-binding protein | *fbpC* | Iron(III) ABC transporter, ATP-binding protein | 33.2 | 235/352 | 143 | 7 | 2.20 e-11 | 105.0 |
| NM221_01540 | LicD family protein | *licD* | Phosphocholine transferase | 33.1 | 278/265 | 158 | 10 | 2.23 e-33 | 119.0 |
| NM221_00190 | ATP-binding cassette domain-containing protein | *hitC* | Iron(III) ABC transporter, ATP-binding protein | 33.0 | 264/351 | 170 | 3 | 3.54 e-25 | 144.0 |
| NM221_02185 | Cell division ATP-binding protein FtsE | *hitC* | Iron(III) ABC transporter, ATP-binding protein | 33.0 | 218/351 | 141 | 5 | 1.51 e-17 | 120.0 |
| NM221_00155 | ATP-binding cassette domain-containing protein | *cylA* | ABC (ATP-binding cassette) transporter CylA | 33.0 | 197/309 | 123 | 4 | 3.77 e-29 | 110.0 |
| NM221_00585 | Metal ABC transporter ATP-binding protein | *fepC* | Iron-enterobactin transporter ATP-binding protein | 33.0 | 209/264 | 134 | 4 | 8.91 e-28 | 103.0 |
| NM221_06305 | ABC transporter ATP-binding protein/permease | *ybtP* | Yersiniabactin ABC transporter ATP-binding/permease protein YbtP | 32.9 | 493/600 | 326 | 2 | 7.99 e-71 | 276.0 |
| NM221_05245 | V-type ATP synthase subunit B | *yscN* | Type III secretion system ATPase YscN | 32.9 | 328/439 | 203 | 7 | 2.87 e-41 | 149.0 |
| NM221_07730 | AAA family ATPase | *AHA_1389* | CobQ/CobB/MinD/ParA family protein | 32.8 | 244/264 | 157 | 3 | 9.46 e-24 | 133.0 |
| NM221_02910 | Metal ABC transporter substrate-binding protein | *lmb* | Laminin-binding surface protein | 32.8 | 287/306 | 172 | 6 | 6.31 e-37 | 132.0 |
| NM221_05080 | 16S rRNA (uracil(1498)-N(3))-methyltransferase | *lpg2936* | Dot/Icm type IV secretion system effector | 32.7 | 217/244 | 137 | 4 | 1.81 e-28 | 105.0 |
| NM221_01560 | Energy-coupling factor transporter ATPase | *cylA* | ABC (ATP-binding cassette) transporter CylA | 32.6 | 215/309 | 138 | 4 | 1.74 e-26 | 102.0 |
| NM221_06070 | DNA starvation/stationary phase protection protein | *napA* | Neutrophil activating protein NapA | 32.6 | 141/144 | 95 | 0 | 1.58 e-25 | 92.0 |
| NM221_06085 | Asparagine synthase (glutamine-hydrolyzing) | *wbtH* | Asparagine synthase (glutamine-hydrolyzing) | 32.3 | 635/628 | 385 | 19 | 4.36 e-75 | 288.0 |
| NM221_00085 | ABC transporter ATP-binding protein/permease | *ybtP* | Yersiniabactin ABC transporter ATP-binding/permease protein YbtP | 32.3 | 508/600 | 329 | 9 | 8.87 e-78 | 254.0 |
| NM221_05565 | Type VII secretion protein EssB | *essB* | Type VII secretion system protein EssB, monotopic membrane protein | 32.1 | 343/444 | 228 | 3 | 2.19 e-33 | 170.0 |
| NM221_03835 | Aminotransferase class I/II-fold pyridoxal phosphate-dependent enzyme | *Cj1436c* | Aminotransferase | 32.0 | 356/390 | 212 | 12 | 1.59 e-32 | 171.0 |
| NM221_07805 | ABC transporter ATP-binding protein | *hitC* | Iron(III) ABC transporter, ATP-binding protein | 31.9 | 226/351 | 135 | 5 | 1.68 e-30 | 113.0 |
| NM221_00320 | ABC transporter ATP-binding protein/permease | *fbpC* | Iron(III) ABC transporter, ATP-binding protein | 31.9 | 226/352 | 144 | 5 | 1.91 e-10 | 109.0 |
| NM221_04265 | ABC transporter ATP-binding protein | *hitC* | Iron(III) ABC transporter, ATP-binding protein | 31.8 | 217/351 | 139 | 5 | 5.66 e-24 | 100.0 |
| NM221_05310 | ATP-binding cassette domain-containing protein | *hitC* | Iron(III) ABC transporter, ATP-binding protein | 31.5 | 222/351 | 141 | 7 | 1.29 e-24 | 96.7 |
| NM221_00090 | ABC transporter ATP-binding protein/permease | *ybtQ* | Yersiniabactin ABC transporter ATP-binding/permease protein YbtQ | 31.4 | 478/600 | 315 | 6 | 9.88 e-51 | 222.0 |
| NM221_01115 | V-type ATP synthase subunit B | *fliI* | Flagellum-specific ATP synthase | 31.3 | 323/439 | 213 | 4 | 1.56 e-25 | 150.0 |
| NM221_01480 | DNA/RNA non-specific endonuclease | *mf3* | Deoxyribonuclease | 31.1 | 177/268 | 96 | 5 | 8.95 e-20 | 82.8 |
| NM221_05400 | Signal peptidase I | *sipA* | Signal peptidase I | 31.1 | 122/183 | 78 | 3 | 3.79 e-14 | 64.7 |
| NM221_04865 | ATP-binding cassette domain-containing protein | *cesC* | ABC transporter ATP-binding protein, CesC | 30.8 | 240/291 | 152 | 6 | 2.72 e-29 | 110.0 |
| NM221_03080 | ABC transporter ATP-binding protein/permease | *ybtP* | Yersiniabactin ABC transporter ATP-binding/permease protein YbtP | 30.4 | 513/600 | 326 | 9 | 4.55 e-41 | 199.0 |
| NM221_06605 | ABC transporter ATP-binding protein | *cesC* | ABC transporter ATP-binding protein, CesC | 30.4 | 207/291 | 137 | 5 | 3.86 e-25 | 99.0 |
| NM221_01545 | LicD family protein | *licD* | Phosphocholine transferase | 30.3 | 274/265 | 170 | 9 | 7.01 e-16 | 113.0 |
| NM221_03415 | LicD family protein | *licD* | Phosphocholine transferase | 30.3 | 277/265 | 159 | 8 | 1.02 e-27 | 105.0 |
| NM221_03495 | Hypothetical protein | *ureG* | Urease accessory protein | 30.1 | 146/199 | 86 | 9 | 9.5 e-07 | 45.1 |

**Table S6.** Virulence factors present in *P. micra* PM89KC-G-1 isolate, using DIAMOND against the Virulence Factor Database (version 2021-10-04).

| **Query locus tag** | **Query description** | **Gene** | **Subject description** | **Percentage of identical matches** | **Alignment length/Gene length** | **Mismatch** | **Number of gap openings** | **E-value** | **Bit score** |
| --- | --- | --- | --- | --- | --- | --- | --- | --- | --- |
| NM219_02920 | Chaperonin GroEL | *groEL* | Chaperonin GroEL | 73.8 | 542/542 | 139 | 2 | 5.31 e-255 | 743.0 |
| NM219_06770 | Elongation factor Tu | *tufA* | Elongation factor Tu | 70.7 | 396/394 | 113 | 3 | 2.15 e-193 | 578.0 |
| NM219_04900 | ATP-dependent Clp endopeptidase proteolytic subunit ClpP | *clpP* | ATP-dependent Clp endopeptidase proteolytic subunit ClpP | 64.6 | 192/198 | 68 | 0 | 4.66 e-72 | 254.0 |
| NM219_05645 | UDP-glucose 4-epimerase GalE | *galE* | UDP-glucose 4-epimerase | 58.9 | 336/338 | 137 | 1 | 6.67 e-134 | 420.0 |
| NM219_01810 | Peptide-methionine (R)-S-oxide reductase MsrB | *msrA/BpilB* | Trifunctional thioredoxin/methionine sulfoxide reductase A/B protein | 57.6 | 330/522 | 126 | 4 | 1.13 e-114 | 381.0 |
| NM219_03245 | Isoprenyl transferase | *cpsA* | Undecaprenyl diphosphate synthase | 48.7 | 234/271 | 116 | 1 | 1.47 e-60 | 229.0 |
| NM219_05590 | WXG100 family type VII secretion target | *esxA* | Type VII secretion system secreted protein EsxA | 47.3 | 93/97 | 49 | 0 | 3.85 e-11 | 92.8 |
| NM219_03445 | AAA family ATPase | *clpC* | Endopeptidase Clp ATP-binding chain C | 46.9 | 778/820 | 340 | 6 | 2.98 e-212 | 660.0 |
| NM219_02370 | Superoxide dismutase | *sodB* | Superoxide dismutase | 46.9 | 194/196 | 96 | 1 | 1.50 e-46 | 190.0 |
| NM219_00285 | Aspartate carbamoyltransferase | *pyrB* | Aspartate carbamoyltransferase | 46.5 | 303/306 | 155 | 5 | 6.25 e-66 | 244.0 |
| NM219_01045 | 3-hydroxyacyl-ACP dehydratase FabZ | *fabZ* | (3R)-hydroxymyristoyl ACP dehydratase | 46.1 | 128/173 | 67 | 2 | 4.04 e-31 | 107.0 |
| NM219_05565 | Type VII secretion protein EssC | *essC* | Type VII secretion system protein EssC, FtsK/SpoIIIE family ATPase | 46.0 | 1324/1479 | 699 | 9 | 0.0 | 1169.0 |
| NM219_01075 | RdgB/HAM1 family non-canonical purine NTP pyrophosphatase | *orfM* | Deoxyribonucleotide triphosphate pyrophosphatase | 45.6 | 193/195 | 100 | 3 | 1.18 e-42 | 139.0 |
| NM219_01035 | 3-oxoacyl-[acyl-carrier-protein] reductase | *cylG* | 3-ketoacyl-ACP-reductase CylG | 45.5 | 244/240 | 123 | 4 | 4.30 e-50 | 199.0 |
| NM219_04555 | ATP-dependent Clp protease ATP-binding subunit | *clpC* | Endopeptidase Clp ATP-binding chain C | 45.3 | 811/820 | 426 | 6 | 5.53 e-209 | 647.0 |
| NM219_00280 | Polysaccharide biosynthesis protein | *cap8D* | Capsular polysaccharide synthesis enzyme Cap8D | 44.1 | 508/607 | 277 | 7 | 5.21 e-121 | 407.0 |
| NM219_05495 | YebC/PmpR family DNA-binding transcriptional regulator | *CBU_1566* | Coxiella Dot/Icm type IVB secretion system translocated effector | 44.1 | 245/244 | 134 | 3 | 9.49 e-51 | 201.0 |
| NM219_02220 | Ribulose-phosphate 3-epimerase | *rpe* | Ribulose-phosphate 3-epimerase | 43.0 | 200/222 | 113 | 1 | 2.60 e-42 | 181.0 |
| NM219_03155 | ABC transporter ATP-binding protein | *hitC* | Iron(III) ABC transporter, ATP-binding protein | 42.6 | 242/351 | 127 | 4 | 3.14 e-35 | 170.0 |
| NM219_03395 | Sugar transferase | *bplG* | Probable sugar transferase | 42.6 | 197/197 | 108 | 2 | 5.82 e-42 | 139.0 |
| NM219_00275 | UTP-glucose-1-phosphate uridylyltransferase | *hasC* | UTP-glucose-1-phosphate uridylyltransferase | 42.5 | 294/295 | 155 | 6 | 1.79 e-58 | 227.0 |
| NM219_00960 | ABC transporter ATP-binding protein | *hitC* | Iron(III) ABC transporter, ATP-binding protein | 42.4 | 236/351 | 136 | 0 | 7.95 e-47 | 199.0 |
| NM219_01240 | GspE/PulE family protein | *exeE* | General secretory pathway protein E | 41.1 | 375/501 | 218 | 3 | 1.12 e-84 | 305.0 |
| NM219_04185 | Bifunctional (p)ppGpp synthetase/guanosine-3',5'-bis(diphosphate) 3'-pyrophosphohydrolase | *relA* | Probable GTP pyrophosphokinase RelA (ATP:GTP 3'-pyrophosphotransferase) (PPGPP synthetase I) ((P)PPGPP synthetase) (GTP diphosphokinase) | 40.4 | 727/790 | 418 | 6 | 5.61 e-179 | 566.0 |
| NM219_04995 | Peptidylprolyl isomerase | *lirB* | Dot/Icm type IV secretion system effector LirB | 40.4 | 193/188 | 65 | 5 | 6.41 e-16 | 108.0 |
| NM219_00185 | Cna B-type domain-containing protein | *cna* | Collagen adhesin precursor | 39.8 | 734/1183 | 397 | 11 | 4.42 e-152 | 535.0 |
| NM219_04340 | phosphoribosylformylglycinamidine cyclo-ligase | *purM* | Phosphoribosylaminoimidazole synthetase | 39.7 | 345/347 | 200 | 5 | 7.75 e-70 | 257.0 |
| NM219_00740 | 3-oxoacyl-ACP reductase FabG | *cylG* | 3-ketoacyl-ACP-reductase CylG | 39.4 | 241/240 | 132 | 5 | 7.16 e-38 | 168.0 |
| NM219_00930 | ABC transporter ATP-binding protein | *mntA* | ABC transporter ATP-binding protein MntA | 38.9 | 226/251 | 134 | 1 | 3.27 e-51 | 164.0 |
| NM219_01535 | Glycosyltransferase family 2 protein | *FTT_0797* | Glycosyltransferase family 2 protein | 38.9 | 216/319 | 119 | 2 | 1.52 e-45 | 154.0 |
| NM219_00610 | Lipoate protein ligase | *lplA1* | Lipoate protein ligase | 38.3 | 329/331 | 195 | 5 | 1.47 e-55 | 222.0 |
| NM219_02805 | Helix-turn-helix domain-containing protein | *cylR2* | Cytolysin regulator R2 | 38.3 | 47/66 | 29 | 0 | 2.44 e+09 | 39.7 |
| NM219_04290 | Helix-turn-helix domain-containing protein | *cylR2* | Cytolysin regulator R2 | 37.9 | 58/66 | 36 | 0 | 8.00 e-08 | 42.0 |
| NM219_06415 | ABC transporter ATP-binding protein/permease | *msbA* | Lipid transporter ATP-binding/permease | 37.4 | 406/587 | 227 | 10 | 4.59 e-56 | 236.0 |
| NM219_05015 | Signal peptidase II | *lspA* | Signal peptidase II | 37.2 | 148/154 | 91 | 2 | 2.53 e-08 | 89.4 |
| NM219_04095 | Pantetheine-phosphate adenylyltransferase | *kdtB* | Lipopolysaccharide core biosynthesis protein | 37.1 | 151/157 | 94 | 1 | 4.10 e-13 | 102.0 |
| NM219_07450 | Response regulator transcription factor | *phoP* | Possible two component system response transcriptional positive regulator PhoP | 36.9 | 225/247 | 133 | 4 | 5.78 e-27 | 140.0 |
| NM219_01565 | Energy-coupling factor transporter ATPase | *cylA* | ABC (ATP-binding cassette) transporter CylA | 36.7 | 210/309 | 120 | 6 | 1.76 e-13 | 110.0 |
| NM219_04380 | Response regulator transcription factor | *bfmR* | Biofilm-controlling response regulator | 36.6 | 224/238 | 139 | 2 | 2.68 e-44 | 145.0 |
| NM219_01385 | ABC transporter ATP-binding protein | *hitC* | Iron(III) ABC transporter, ATP-binding protein | 36.6 | 216/351 | 120 | 5 | 7.87 e-41 | 140.0 |
| NM219_03945 | Response regulator transcription factor | *bfmR* | Biofilm-controlling response regulator | 36.5 | 181/238 | 112 | 2 | 8.53 e-28 | 103.0 |
| NM219_01575 | NFACT family protein | *fbpA* | Fibronectin-binding protein FbpA | 36.2 | 578/591 | 354 | 10 | 6.02 e-92 | 330.0 |
| NM219_02130 | ABC transporter ATP-binding protein | *hitC* | Iron(III) ABC transporter, ATP-binding protein | 36.2 | 221/351 | 129 | 4 | 8.75 e-33 | 119.0 |
| NM219_00990 | Acyl carrier protein | *acpXL* | Acyl carrier protein | 36.2 | 69/78 | 44 | 0 | 1.13 e-09 | 47.0 |
| NM219_03855 | Phosphopantetheine-binding protein | *acpXL* | Acyl carrier protein | 36.2 | 47/78 | 30 | 0 | 1.73 e-05 | 36.2 |
| NM219_02255 | D-alanine-poly(phosphoribitol) ligase subunit DltC | *clbE* | Colibactin biosynthesis aminomalonyl-acyl carrier protein ClbE | 36.0 | 50/82 | 32 | 0 | 5.57 e+11 | 35.0 |
| NM219_02315 | ABC transporter ATP-binding protein | *hitC* | Iron(III) ABC transporter, ATP-binding protein | 35.9 | 309/351 | 190 | 3 | 6.61 e-58 | 188.0 |
| NM219_02605 | Phospho-sugar mutase | *yhxB/manB* | Phosphomannomutase | 35.7 | 554/550 | 309 | 13 | 7.97 e-75 | 284.0 |
| NM219_05310 | ABC transporter ATP-binding protein/permease | *msbA* | Lipid transporter ATP-binding/permease | 35.2 | 520/587 | 311 | 6 | 2.72 e-88 | 283.0 |
| NM219_06815 | Ornithine carbamoyltransferase | *pyrB* | Aspartate carbamoyltransferase | 34.9 | 186/306 | 118 | 3 | 1.01 e-03 | 85.1 |
| NM219_03100 | Bifunctional UDP-N-acetylglucosamine diphosphorylase/glucosamine-1-phosphate N-acetyltransferase GlmU | *glmU* | UDP-N-acetylglucosamine pyrophosphorylase/glucosamine-1-phosphate N-acetyltransferase | 34.8 | 454/455 | 276 | 9 | 1.08 e-73 | 236.0 |
| NM219_02545 | Lipoate-protein ligase | *lplA1* | Lipoate protein ligase | 34.7 | 248/331 | 158 | 3 | 1.78 e-29 | 154.0 |
| NM219_00925 | Zinc ABC transporter substrate-binding protein | *lpeA* | Lipoprotein promoting cell invasion | 34.6 | 324/310 | 188 | 7 | 7.07 e-51 | 168.0 |
| NM219_00120 | S8 family serine peptidase | *scpB* | Streptococcal C5a peptidase | 34.5 | 946/1150 | 515 | 21 | 7.60 e-126 | 462.0 |
| NM219_04425 | ABC transporter ATP-binding protein | *hitC* | Iron(III) ABC transporter, ATP-binding protein | 34.5 | 220/351 | 131 | 8 | 2.08 e-29 | 109.0 |
| NM219_01485 | ABC transporter ATP-binding protein | *cesC* | ABC transporter ATP-binding protein, CesC | 34.2 | 222/291 | 140 | 3 | 1.38 e-26 | 143.0 |
| NM219_01875 | ABC transporter ATP-binding protein | *cylA* | ABC (ATP-binding cassette) transporter CylA | 34.2 | 225/309 | 140 | 5 | 5.90 e-15 | 115.0 |
| NM219_03350 | Peptidylprolyl isomerase | *PEB4* | Major antigenic peptide PEB-cell binding factor | 34.0 | 253/273 | 152 | 8 | 5.26 e-28 | 105.0 |
| NM219_05740 | Iron ABC transporter permease | *shuU* | Permease of iron compound ABC transport system | 33.9 | 283/318 | 178 | 4 | 7.94 e-35 | 166.0 |
| NM219_04155 | Ribosome maturation factor RimP | *CBU_1434* | Coxiella Dot/Icm type IVB secretion system translocated effector | 33.9 | 124/153 | 81 | 1 | 3.91 e-17 | 70.9 |
| NM219_07865 | Response regulator transcription factor | *bfmR* | Biofilm-controlling response regulator | 33.8 | 222/238 | 142 | 4 | 2.53 e-34 | 120.0 |
| NM219_00345 | Glycine C-acetyltransferase | *wcbT* | Acyl-CoA transferase | 33.7 | 380/439 | 248 | 3 | 8.60 e-54 | 221.0 |
| NM219_00270 | DegT/DnrJ/EryC1/StrS family aminotransferase | *bplF* | Lipopolysaccharide biosynthesis protein | 33.6 | 411/410 | 234 | 9 | 1.41 e-54 | 224.0 |
| NM219_07900 | LCP family protein | *bpsX* | LytR family transcriptional regulator | 33.5 | 188/304 | 116 | 3 | 3.78 e-30 | 114.0 |
| NM219_07315 | ATP-binding cassette domain-containing protein | *cesC* | ABC transporter ATP-binding protein, CesC | 33.3 | 228/291 | 148 | 3 | 5.04 e-18 | 121.0 |
| NM219_07285 | Response regulator transcription factor | *bfmR* | Biofilm-controlling response regulator | 33.3 | 222/238 | 137 | 3 | 8.29 e-14 | 105.0 |
| NM219_02580 | Phosphonate ABC transporter ATP-binding protein | *fbpC* | Iron(III) ABC transporter, ATP-binding protein | 33.2 | 235/352 | 143 | 7 | 2.20 e-11 | 105.0 |
| NM219_01540 | LicD family protein | *licD* | Phosphocholine transferase | 33.1 | 278/265 | 158 | 10 | 2.23 e-33 | 119.0 |
| NM219_06310 | ABC transporter ATP-binding protein/permease | *ybtQ* | Yersiniabactin ABC transporter ATP-binding/permease protein YbtQ | 33.0 | 488/600 | 319 | 6 | 9.48 e-56 | 236.0 |
| NM219_00205 | ATP-binding cassette domain-containing protein | *hitC* | Iron(III) ABC transporter, ATP-binding protein | 33.0 | 264/351 | 170 | 3 | 3.54 e-25 | 144.0 |
| NM219_02185 | Cell division ATP-binding protein FtsE | *hitC* | Iron(III) ABC transporter, ATP-binding protein | 33.0 | 218/351 | 141 | 5 | 1.51 e-17 | 120.0 |
| NM219_00170 | ATP-binding cassette domain-containing protein | *cylA* | ABC (ATP-binding cassette) transporter CylA | 33.0 | 197/309 | 123 | 4 | 3.77 e-29 | 110.0 |
| NM219_00600 | Metal ABC transporter ATP-binding protein | *fepC* | Iron-enterobactin transporter ATP-binding protein | 33.0 | 209/264 | 134 | 4 | 8.91 e-28 | 103.0 |
| NM219_06420 | ABC transporter ATP-binding protein/permease | *ybtP* | Yersiniabactin ABC transporter ATP-binding/permease protein YbtP | 32.9 | 493/600 | 326 | 2 | 7.99 e-71 | 276.0 |
| NM219_05255 | V-type ATP synthase subunit B | *yscN* | Type III secretion system ATPase YscN | 32.9 | 328/439 | 203 | 7 | 2.87 e-41 | 149.0 |
| NM219_07820 | AAA family ATPase | *AHA_1389* | CobQ/CobB/MinD/ParA family protein | 32.8 | 244/264 | 157 | 3 | 9.46 e-24 | 133.0 |
| NM219_02905 | Metal ABC transporter substrate-binding protein | *lmb* | Laminin-binding surface protein | 32.8 | 287/306 | 172 | 6 | 6.31 e-37 | 132.0 |
| NM219_06305 | ABC transporter ATP-binding protein/permease | *ybtQ* | Yersiniabactin ABC transporter ATP-binding/permease protein YbtQ | 32.7 | 489/600 | 308 | 6 | 3.94 e-70 | 234.0 |
| NM219_05090 | 16S rRNA (uracil(1498)-N(3))-methyltransferase | *lpg2936* | Dot/Icm type IV secretion system effector | 32.7 | 217/244 | 137 | 4 | 1.81 e-28 | 105.0 |
| NM219_01560 | Energy-coupling factor transporter ATPase | *cylA* | ABC (ATP-binding cassette) transporter CylA | 32.6 | 215/309 | 138 | 4 | 1.74 e-26 | 102.0 |
| NM219_06075 | DNA starvation/stationary phase protection protein | *napA* | Neutrophil activating protein NapA | 32.6 | 141/144 | 95 | 0 | 1.58 e-25 | 92.0 |
| NM219_00095 | ABC transporter ATP-binding protein/permease | *ybtP* | Yersiniabactin ABC transporter ATP-binding/permease protein YbtP | 32.3 | 508/600 | 329 | 9 | 8.87 e-78 | 254.0 |
| NM219_06090 | Asparagine synthase (glutamine-hydrolyzing) | *wbtH* | Asparagine synthase (glutamine-hydrolyzing) | 32.1 | 635/628 | 386 | 19 | 3.3 e-89 | 286.0 |
| NM219_05570 | Type VII secretion protein EssB | *essB* | Type VII secretion system protein EssB, monotopic membrane protein | 32.1 | 343/444 | 228 | 3 | 2.19 e-33 | 170.0 |
| NM219_03845 | Aminotransferase class I/II-fold pyridoxal phosphate-dependent enzyme | *Cj1436c* | Aminotransferase | 32.0 | 356/390 | 212 | 12 | 1.59 e-32 | 171.0 |
| NM219_07885 | ABC transporter ATP-binding protein | *hitC* | Iron(III) ABC transporter, ATP-binding protein | 31.9 | 226/351 | 135 | 5 | 1.68 e-30 | 113.0 |
| NM219_00335 | ATP-binding cassette domain-containing protein | *fbpC* | Iron(III) ABC transporter, ATP-binding protein | 31.9 | 226/352 | 144 | 5 | 1.05 e-10 | 110.0 |
| NM219_04275 | ABC transporter ATP-binding protein | *hitC* | Iron(III) ABC transporter, ATP-binding protein | 31.8 | 217/351 | 139 | 5 | 5.66 e-24 | 100.0 |
| NM219_01480 | DNA/RNA non-specific endonuclease | *mf3* | Deoxyribonuclease | 31.6 | 177/268 | 95 | 5 | 4.67 e-20 | 83.6 |
| NM219_05320 | ATP-binding cassette domain-containing protein | *hitC* | Iron(III) ABC transporter, ATP-binding protein | 31.5 | 222/351 | 141 | 7 | 1.29 e-24 | 96.7 |
| NM219_00100 | ABC transporter ATP-binding protein/permease | *ybtQ* | Yersiniabactin ABC transporter ATP-binding/permease protein YbtQ | 31.4 | 478/600 | 315 | 6 | 9.88 e-51 | 222.0 |
| NM219_06195 | Cna B-type domain-containing protein | *cna* | Collagen adhesin precursor | 31.3 | 1122/1183 | 630 | 48 | 5.48 e-90 | 360.0 |
| NM219_05405 | Signal peptidase I | *sipA* | Signal peptidase I | 31.1 | 122/183 | 78 | 3 | 3.79 e-14 | 64.7 |
| NM219_01130 | V-type ATP synthase subunit B | *fliI* | Flagellum-specific ATP synthase | 31.0 | 323/439 | 214 | 4 | 1.51 e-24 | 147.0 |
| NM219_04875 | ATP-binding cassette domain-containing protein | *cesC* | ABC transporter ATP-binding protein, CesC | 30.8 | 240/291 | 152 | 6 | 2.72 e-29 | 110.0 |
| NM219_00325 | Cna B-type domain-containing protein | *cna* | Collagen adhesin precursor | 30.7 | 1148/1183 | 646 | 55 | 8.78 e-86 | 342.0 |
| NM219_03075 | ABC transporter ATP-binding protein/permease | *ybtP* | Yersiniabactin ABC transporter ATP-binding/permease protein YbtP | 30.4 | 513/600 | 326 | 9 | 4.55 e-41 | 199.0 |
| NM219_06710 | ABC transporter ATP-binding protein | *cesC* | ABC transporter ATP-binding protein, CesC | 30.4 | 207/291 | 137 | 5 | 1.44 e-09 | 100.0 |
| NM219_01545 | LicD family protein | *licD* | Phosphocholine transferase | 30.3 | 274/265 | 170 | 9 | 7.01 e-16 | 113.0 |
| NM219_03415 | LicD family protein | *licD* | Phosphocholine transferase | 30.3 | 277/265 | 159 | 8 | 1.02 e-27 | 105.0 |
| NM219_01125 | V-type ATP synthase subunit A | *pscN* | Type III secretion system ATPase PscN | 30.2 | 344/440 | 216 | 9 | 4.75 e-30 | 120.0 |
| NM219_03495 | Hypothetical protein | *ureG* | Urease accessory protein | 30.1 | 146/199 | 86 | 9 | 9.5 e-07 | 45.1 |

**Table S7.** Diversity and richness in metatranscriptome analysis at species level.

| **Sample** | **Chao1** | **Shannon** |
| --- | --- | --- |
| T89-FT-N | 894.47 | 3.52 |
| T89-FT-AC | 750.38 | 3.35 |

**Table S8.** Identification and re-annotation of top 20 most expressed genes by *P. micra* in adenocarcinoma tissue in PM89KC-AC-1.

| **KEGG** | **KEGG**  **description** | **Gene ID (UHGG)** | **Present in PM89KC-AC-1 genome** | **Identity** | **Mismatch** | **Position in PM89KC-AC-1** | **CDS** | **Description (PM89KC-AC-1)** | | **Annotation by Phyre2** | **Confidence (%)** | **Coverage (%)** |
| --- | --- | --- | --- | --- | --- | --- | --- | --- | --- | --- | --- | --- |
| K13695 | nlpC; probable lipoprotein NlpC | GUT_GENOME095968_00764 | Yes | 99.46 | 7 | 1633480-1634781 | 1488 | C40 family peptidase | putative cell wall hydrolase phosphatase-associated | | 99.9 | 27 |
| K02437 | gcvH, GCSH; glycine cleavage system H protein | GUT_GENOME095968_01261 | Yes | 100 | 0 | 1192938-1193309 | 1090 | glycine cleavage system protein GcvH | glycine cleavage system h protein | | 100 | 95 |
| K05787 | hupA; DNA-binding protein HU-alpha | GUT_GENOME095968_00315 | Yes | 99.64 | 1 | 672175-672453 | 610 | HU family DNA-binding protein | dna-binding protein hu | | 99.9 | 97 |
| K10793 | prdA; D-proline reductase (dithiol) PrdA [EC:1.21.4.1] | GUT_GENOME095968_00519 \| GUT_GENOME095968_00229 | Yes\|Yes | 99.61 \| 99.53 | 5\|6 | 915093-916379 \| 521012-522298 | 825 \| 484 | glycine/sarcosine/ betaine reductase component B subunit (x2) | beta-subunit 2-hydroxyacyl-coa dehydratase (x2) | | 88.2 \| 89.7 | 13 \| 13 |
| K08304 | mltA; membrane-bound lytic murein transglycosylase A [EC:4.2.2.-] | GUT_GENOME095968_01259 | Yes | 99.54 | 7 | 1190170-1191687 | 1088 | 3D domain containing protein | cell wall binding protein cwp8 | | 100 | 56 |
| K02358 | tuf, TUFM; elongation factor Tu | GUT_GENOME095968_01434 | Yes | 99.92 | 1 | 1403136-1404329 | 1295 | elongation factor TU | elongation factor tu | | 100 | 99 |
| K03111 | ssb; single-strand DNA-binding protein | GUT_GENOME095968_00535\| GUT_GENOME095968_00942 | Yes\|No | 99.56 | 2 | 932206-932658 | 841 | Single-stranded DNA binding protein | single-stranded dna-binding protein \| single-strand binding protein | | 99.9 \| 99.9 | 69 \| 61 |
| K00939 | adk, AK; adenylate kinase [EC:2.7.4.3] | GUT_GENOME095968_00617 | Yes | 95.22 | 30 | 23798-1024445 | 925 | Adenylate kinase | adenylate kinse | | 100 | 98 |
| K03073 | secE; preprotein translocase subunit SecE | GUT_GENOME095968_00300 | Yes | 100 | 0 | 655439-655645 | 596 | protein translocase subunit SecE | preprotein translocase sece subunit | | 99.8 | 94 |
| K02355 | fusA, GFM, EFG; elongation factor G | GUT_GENOME095968_01144\| GUT_GENOME095968_01435 | Yes\|Yes | 99.22 \| 99.95 | 16\|1 | 259165-261225 \| 1404350-1406425 | 235 \| 1296 | elongation factor G (x2) | elongation factor g (x2) | | 100 \| 100 | 95 \| 95 |
| K00639 | kbl, GCAT; glycine C-acetyltransferase [EC:2.3.1.29] | GUT_GENOME095968_00913 | Yes | 99.49 | 6 | 88685-89869 | 66 | glycine c-acetyltransferase | 2-amino-3-ketobutyrate coenzyme a ligase | | 100 | 99 |
| K03040 | rpoA; DNA-directed RNA polymerase subunit alpha [EC:2.7.7.6] | GUT_GENOME095968_00612 | Yes | 100 | 0 | 1021330-1022271 | 919 | DNA-directed RNA polymerase subunit alpha | dna-directed rna polymerase subunit alpha | | 100 | 97 |
| K00656 | E2.3.1.54, pflD; formate C-acetyltransferase [EC:2.3.1.54] | GUT_GENOME095968_01464\| GUT_GENOME095968_01569 | Yes\|Yes | 97.20 \| 99.42 | 43\|13 | 1424097-1425632 \| 1529327-1531573 | 1313 \| 1418 | YjjI family glycine radical enzime \| formate C-acetil transferase | pfl2/glycerol dehydratase family glycyl radical enzyme\| PFL-like glycyl radical enzymes | | 100 \| 100 | 87 \| 99 |
| K04047 | dps; starvation-inducible DNA-binding protein | GUT_GENOME095968_01345 | Yes | 95.5 | 20 | 1287445-1287888 | 1181 | DNA starvation/ stationary phase protection protein | Ferritin-like | | 100 | 95 |
| K03621 | plsX; phosphate acyltransferase [EC:2.3.1.274] | GUT_GENOME095968_00443\| GUT_GENOME095968_00513 | Yes\|Yes | 99.1 \| 99.31 | 9\|8 | 835822-836817 \| 910352-911512 | 752 \| 821 | phosphate acyltransferase PlsX \| glycine reductase | Isocitrate/Isopropylmalate dehydrogenase-like (x2) | | 100 \| 100 | 95 \| 67 |
| K02518 | infA; translation initiation factor IF-1 | GUT_GENOME095968_00615 | Yes | 97.26 | 6 | 1023265-1023483 | 923 | translation initiation factor IF-1 | translation initiation factor if-1 | | 100 | 100 |
| K02601 | nusG; transcriptional antiterminator NusG | GUT_GENOME095968_00301 | Yes | 99.82 | 16\|1 | 655679-656218 | 597 | transcription termination/ antitermination protein NusG | transcription antitermination protein nusg | | 100 | 92 |
| K00384 | trxB, TRR; thioredoxin reductase (NADPH) [EC:1.8.1.9] | GUT_GENOME095968_00520 | Yes | 99.89 | 16\|1 | 916432-917361 | 826 | FAD-dependent oxidoreductase | thioredoxin reductase | | 100 | 97 |
| K03076 | secY; preprotein translocase subunit SecY | GUT_GENOME095968_00618 | Yes | 99.77 | 3 | 1024463-1025740 | 926 | protein translocase subunit SecY | preprotein translocase subunit secy | | 100 | 93 |
| K01784 | galE, GALE; UDP-glucose 4-epimerase [EC:5.1.3.2] | GUT_GENOME095968_00912\| GUT_GENOME095968_01266 | Yes\|Yes | 99.68 \| 96.56 | 3\|35 | 87716-88663 \| 1198242-1199258 | 65 \| 1095 | NAD-dependent epimerase/ dehydratase family \| UDP-glucose 4 epimerase GalE | l-threonine 3-dehydrogenase\| udp-glucose 4-epimerase | | 100 \| 100 | 99 \| 99 |
